# Beyond the Biosynthetic Gene Cluster Paradigm: Genome-Wide Coexpression Networks Connect Clustered and Unclustered Transcription Factors to Secondary Metabolic Pathways

**DOI:** 10.1128/Spectrum.00898-21

**Published:** 2021-09-15

**Authors:** Min Jin Kwon, Charlotte Steiniger, Timothy C. Cairns, Jennifer H. Wisecaver, Abigail L. Lind, Carsten Pohl, Carmen Regner, Antonis Rokas, Vera Meyer

**Affiliations:** a Chair of Applied and Molecular Microbiology, Institute of Biotechnology, Technische Universität Berlin, Berlin, Germany; b Department of Biochemistry, Center for Plant Biology, Purdue University, West Lafayette, Indiana, USA; c Department of Biological Sciences, Vanderbilt University, Nashville, Tennessee, USA; d Gladstone Institute for Data Science and Biotechnology, San Francisco, California, USA; e Department of Biomedical Informatics, Vanderbilt University School of Medicine, Nashville, Tennessee, USA; Universidade de Sao Paulo

**Keywords:** filamentous fungi, *Aspergillus niger*, secondary metabolite gene clusters, gene coexpression, correlation network, natural product, specialized metabolism, genetic network, gene regulation

## Abstract

Fungal secondary metabolites are widely used as therapeutics and are vital components of drug discovery programs. A major challenge hindering discovery of novel secondary metabolites is that the underlying pathways involved in their biosynthesis are transcriptionally silent under typical laboratory growth conditions, making it difficult to identify the transcriptional networks that they are embedded in. Furthermore, while the genes participating in secondary metabolic pathways are typically found in contiguous clusters on the genome, known as biosynthetic gene clusters (BGCs), this is not always the case, especially for global and pathway-specific regulators of pathways’ activities. To address these challenges, we used 283 genome-wide gene expression data sets of the ascomycete cell factory Aspergillus niger generated during growth under 155 different conditions to construct two gene coexpression networks based on Spearman’s correlation coefficients (SCCs) and on mutual rank-transformed Pearson’s correlation coefficients (MR-PCCs). By mining these networks, we predicted six transcription factors, named MjkA to MjkF, to regulate secondary metabolism in A. niger. Overexpression of each transcription factor using the Tet-On cassette modulated the production of multiple secondary metabolites. We found that the SCC and MR-PCC approaches complemented each other, enabling the delineation of putative global (SCC) and pathway-specific (MR-PCC) transcription factors. These results highlight the potential of coexpression network approaches to identify and activate fungal secondary metabolic pathways and their products. More broadly, we argue that drug discovery programs in fungi should move beyond the BGC paradigm and focus on understanding the global regulatory networks in which secondary metabolic pathways are embedded.

**IMPORTANCE** There is an urgent need for novel bioactive molecules in both agriculture and medicine. The genomes of fungi are thought to contain vast numbers of metabolic pathways involved in the biosynthesis of secondary metabolites with diverse bioactivities. Because these metabolites are biosynthesized only under specific conditions, the vast majority of the fungal pharmacopeia awaits discovery. To discover the genetic networks that regulate the activity of secondary metabolites, we examined the genome-wide profiles of gene activity of the cell factory Aspergillus niger across hundreds of conditions. By constructing global networks that link genes with similar activities across conditions, we identified six putative global and pathway-specific regulators of secondary metabolite biosynthesis. Our study shows that elucidating the behavior of the genetic networks of fungi under diverse conditions harbors enormous promise for understanding fungal secondary metabolism, which ultimately may lead to novel drug candidates.

## INTRODUCTION

Fungal secondary metabolites (SMs) are bioactive, usually low-molecular-weight compounds that have restricted taxonomic distribution and are produced at specific stages of growth and development (1). The most well-known clinical applications of these molecules include their use as antibiotics, cholesterol-lowering agents, and immunosuppressants (e.g., penicillin, statins, and cyclosporins, respectively) (2). However, they also play an important role in drug discovery programs, with recently marketed therapeutics consisting of either fungal SMs or their semisynthetic derivatives (3). In contrast to these contributions to human welfare, fungal SMs also include potent carcinogenic crop contaminants (4), and the mycotoxin-producing capacity of commonly used fungal cell factories in food or biotechnological processes is often either unknown (5) or underestimated (6). Moreover, plant-infecting fungi deploy numerous SMs as virulence factors that facilitate successful infection (7), ultimately destroying enough food for 10% of the human population per year (8). Improved understanding of the genetic, molecular, and biochemical aspects of fungal secondary metabolism thus promises to drive novel medical breakthroughs, while also ensuring improvements in global food safety and security (9).

A common feature of SM-producing fungi is that the genes required for producing a single secondary metabolite are often found in contiguous clusters on the genome, which may facilitate both horizontal gene transfer of SMs and epigenetic regulation via chromatin remodeling (1, 10). Biosynthetic gene clusters (BGCs) typically include a gene encoding a core biosynthetic enzyme, most commonly a nonribosomal peptide synthetase (NRPS), polyketide synthase (PKS), or terpene cyclase, that is responsible for the first metabolic step in product synthesis (11). Additionally, BGCs include genes encoding so-called “tailoring” enzymes, such as P450 monooxygenases or methyltransferases, that modify the molecule produced by the core enzyme (11, 12). Moreover, many BGCs contain either genes encoding putative membrane transporters, which are required for metabolite efflux from the cell in some (13) but not all (14) cases, or additional so-called “resistance” genes, which are necessary for detoxification/self-protection against the molecules produced (15).

Most BGCs are transcriptionally silent under standard laboratory and industrial cultivation conditions, which is a major challenge to the discovery of their cognate molecules (16). Interestingly, many BGCs also contain transcription factor (TF)-encoding genes that regulate their activity (11, 12, 17). In several instances, these TF-encoding genes have been overexpressed to activate transcription of the respective BGC, ultimately leading to the discovery of novel SMs (13, 18–21). However, this strategy cannot be used for the approximately 40% of fungal BGCs that lack a resident TF (17).

An alternative approach to engineering SM-overproducing isolates has been to identify and genetically target global regulators of multiple BGCs. These include epigenetic regulators, notably components of the heterotrimeric velvet complex, which links development, light responses, and SM production in ascomycetes (22). Alternatively, globally acting TFs that coordinate SM biosynthesis with differentiation (e.g., BrlA/StuA) and responses to environmental stimuli, such as pH (PacC) or nitrogen availability (AreA), can be activated using molecular approaches for elevated natural product biosynthesis (1, 17, 23). A limitation to these strategies, however, is that all global regulators discovered to date activate only a fraction of the predicted BGCs in a single genome. For example, deletion of genes predicted to encode the methyltransferase LaeA, which is thought to silence BGC expression by the formation of transcriptionally silent heterochromatin, increased the expression of 7 of 17 BGCs in the biomass-degrading fungus Trichoderma reesei and 13 of 22 BGCs analyzed in the human-infecting mould Aspergillus fumigatus (24, 25).

A final confounding factor in understanding and functionally analyzing fungal BGCs and their products is that there is considerable variation in the degrees to which core, tailoring, transport, and regulatory genes are contiguously clustered in fungal genomes (10). This includes so-called “partial” clusters in which some genes encoding biosynthetic enzymes and transporters are not physically linked with other clustered genes (26, 27), “superclusters” in which two or more NRPS-/PKS-encoding genes reside in close physical proximity (28, 29), and SM-biosynthetic genes that are not contiguously clustered (30).

Consequently, innovative strategies are required both to discover novel transcriptional activators of BGCs and to accurately delineate their boundaries. Over the past several years, an approach that has gained considerable interest has been the utilization of coexpression networks to analyze BGCs, for example, during laboratory culture of industrial isolates (29, 31) or during infectious growth of plant-infecting fungi (32). A limitation to these studies, however, was the relatively small number of conditions tested (up to several dozen), which resulted in the inability to detect the transcriptional activity of numerous BGCs. To overcome this limitation, we recently conducted a meta-analysis of 283 microarray data sets covering 155 different cultivation conditions for the biotechnologically exploited cell factory Aspergillus niger. This data collection covers a diverse range of environmental conditions and genetic perturbations and was used to construct a global gene coexpression network based on Spearman’s correlation coefficient (SCC) (33). We found that 53 of the 81 predicted BGC core genes in A. niger are expressed under at least 1 of the 155 conditions, and we were able to delineate the boundaries of numerous BGCs, including, for example, the partial cluster required for biosynthesis of the siderophore triacetylfusarinine C.

Our analysis also suggested that only a minority of BGCs are coexpressed with their resident TF; specifically, from the 25 of the 53 expressed BGCs that contained a TF, only 8 BGCs were coexpressed with their respective TF. However, we were able to use this network to successfully predict TFs that, independent of their physical location on the genome, regulate multiple BGCs. This relied on the so-called “guilt-by-association” principle, whereby genes that are part of similar (or the same) biosynthetic pathways or genetic networks tend to have highly comparable patterns of gene expression. We functionally analyzed two of these coexpressed TFs (MjkA and MjkB) (Table 1) by generating loss-of-function and gain-of-function A. niger mutants and could indeed demonstrate that their overexpression modulated (either indirectly or directly) the transcriptional activity of 45 (MjkA) and 43 (MjkB) BGC core genes (33).

**TABLE 1:** Selected list of transcription factors analyzed in this study that are coexpressed with BGCs in A. niger

Transcription factor	ORF	Protein domain	No. of coexpressed BGC core genes based on:		Clustered in a BGC	Tet-On-based overexpression phenotype on solid growth medium
			SCC	MR-PCC		
MjkA	An07g07370	Myb-like DNA-binding domain	14		No	Red pigment formation, reduced growth, irregular sclerotia formation
MjkB	An12g07690	Fungal Zn_2_-Cys_6_ binuclear cluster domain	13		No	Red pigment formation
MjkC	An01g14020	Fungal Zn_2_-Cys_6_ binuclear cluster domain	17		No	Yellow pigment formation, reduced growth
MjkD	An07g02880	Fungal-specific transcription factor domain	10		No	Yellow pigment formation
MjkE	An08g11000	Fungal Zn_2_-Cys_6_ binuclear cluster domain	13	1	Yes (BGC 34)	Brown pigment formation
MjkF	An08g10880	Fungal Zn_2_-Cys_6_ binuclear cluster domain	15	1	Yes (BGC 34)	Reduced growth, frequent reversions

Despite the utility of coexpression network analyses, there are several possible limitations to the construction of transcriptional networks based on correlation coefficients like Spearman’s or Pearson’s. In these networks, correlation coefficients are used as weighted edges to connect genes (nodes). One major challenge when constructing these networks is determining the edge weight threshold below which correlation coefficients are excluded from the network, with the goal being to remove nonbiologically relevant gene associations. We have previously used *in silico* data randomization experiments to test the likely threshold of biologically meaningful coexpression based on Spearman (33); however, it is still likely that for many BGCs, the correlation coefficient cutoff chosen (ρ ≥ |0.5|) may be unnecessarily stringent, resulting in false-negative coexpression relationships for BGCs. Additionally, average correlation coefficients can vary by gene function and input data (34). Importantly, in the case of BGC genes that are only expressed under a few or only one specific environmental condition, it is likely that the expression vector for a given BGC gene will be sparse and, therefore, more likely to artificially correlate with other rarely expressed genes rather than with genes with a functional link.

To overcome these challenges, in this study, we reanalyzed the existing A. niger transcriptome data set with a specific focus on A. niger BGCs. First, we generated gene expression modules based on a mutual rank approach, which can capture functional relationships for rarely expressed secondary metabolism genes (34, 35), as we have previously shown in analyses of secondary metabolism in plants (36). We compared this mutual rank strategy with our existing Spearman coexpression data sets and, by integrating both approaches, generated a short list of six TF-encoding genes (including *mjkA* and *mjkB*) that we hypothesized may regulate multiple BGCs. Functional analyses of these genes by overexpression using the Tet-On gene switch revealed that they play multiple roles in the growth, development, and pigment formation of A. niger as assayed by standard growth tests on agar plates and in shake flasks. Moreover, metabolomic profiling revealed a change in the metabolite patterns of the overexpression strains analyzed. Finally, by *in silico* analysis, we generated a list of predicted molecules and associated them with putative BGCs. The methods and resources developed in this study will thus enable the efficient activation of fungal SMs for novel drug discovery programs and other studies. More broadly, our general approach holds potential for deciphering the global regulatory network governing BGCs and secondary metabolic pathways in fungi.

## RESULTS

### Mining coexpression networks to identify biosynthetic and regulatory modules.

Using the SCC approach, we previously estimated the global transcriptional activities of A. niger BGCs among the 283 microarray experiments by assessing the gene expression of the predicted core enzymes (33). These data highlighted that BGC expression varies considerably, with genes encoding core enzymes transcriptionally deployed frequently (several dozen experiments), rarely (>5 experiments), or not at all (which was the case for 28 core genes) (33). We reasoned that this microarray meta-analysis was also a promising resource for further interrogation of BGCs using a mutual rank-transformed Pearson’s correlation coefficient (MR-PCC) approach (34, 36). Transforming PCCs into mutual ranks has been shown to improve the recovery of known pathways as discrete subgraphs (i.e., modules) in global coexpression networks (35). We constructed three MR-PCC networks (NET25, NET10, and NET05), each using a different coexpression threshold for assigning edge weights (i.e., associations) between nodes (i.e., genes) in the network. We then used the graph-clustering method ClusterONE (37) to discover modules of coexpressed genes within the global networks. ClusterONE is unusual among graph clustering methods, e.g., Markov clustering (MCL) (38), due to its ability to assign genes to multiple overlapping modules, which is more reflective of complex biological networks. Networks were ordered on size (i.e., total number of edges between nodes) such that NET25, the most relaxed coexpression threshold, represents the largest network with the largest modules, whereas NET05, the most stringent threshold, represents the smallest network with the smallest modules.

**FIG 1:**
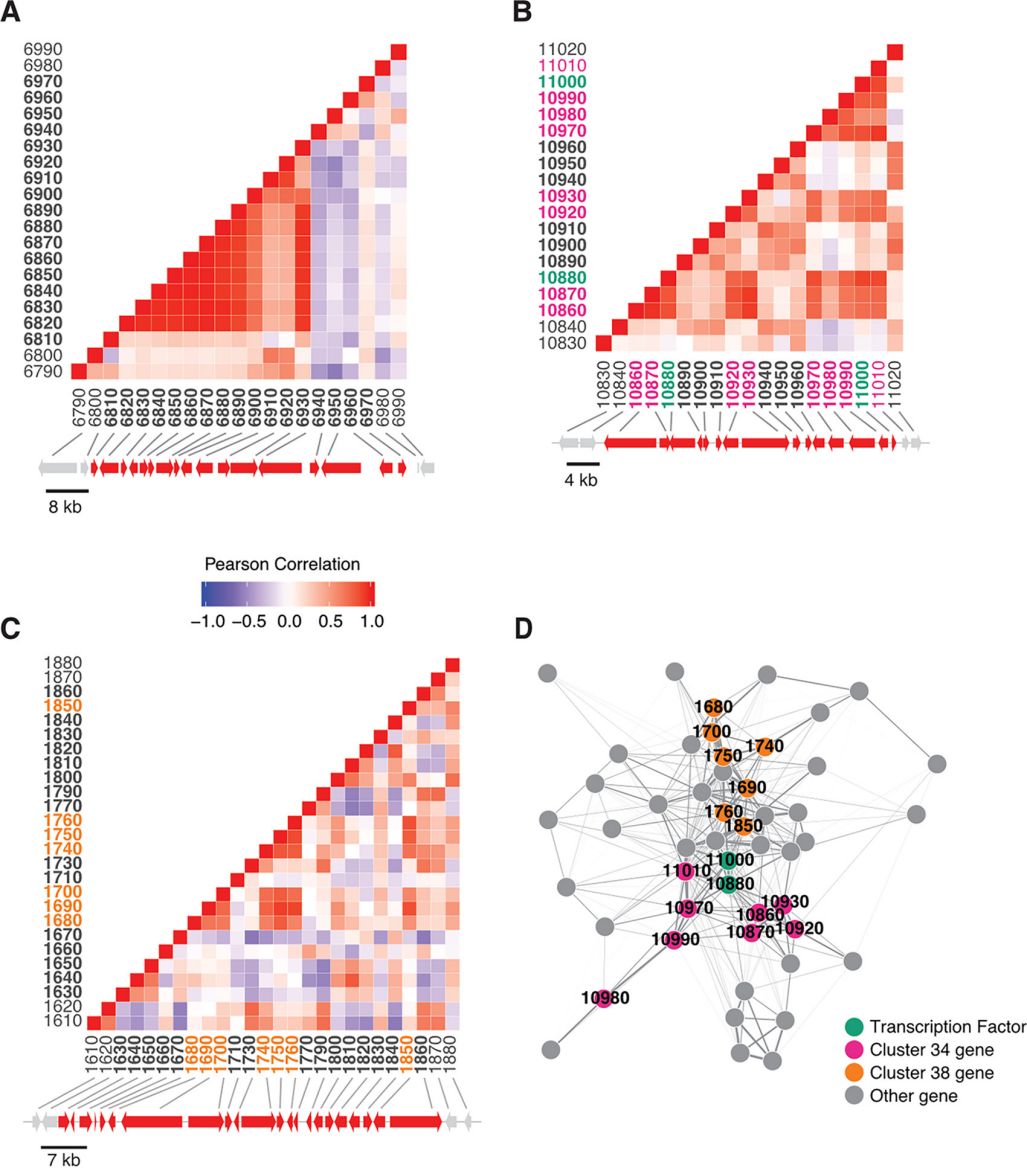
Heatmaps depicting the Pearson’s correlations of coexpression of genes within three canonical BGCs. Across panels A-C, gene ids within the canonical cluster are bolded in the heatmap and the corresponding gene arrow is colored red in the accompanying depiction of the chromosome segment. Two flanking genes are included on either side and corresponding arrows are colored gray. Gene ids have been abbreviated. (A) A significant fraction of genes within the fumonisin metabolic gene cluster are coexpressed. (B) Coexpression of predicted BGC 34, which contains two transcription factors. Both gene ids are colored green in the heatmap, and other clustered gene ids recovered in the metamodule are colored pink. (C) A small fraction of genes within predicted BGC 38 are coexpressed. Genes ids are color coded in the heatmap as in panel A; gene ids recovered in a metamodule are colored orange. (D) Network map of transcription factor metamodule containing all genes coexpressed with both transcription factors across all three network analyses. Nodes in the map represent genes, and edges connecting two genes represent the weight (transformed MR score) for the association. Transcription factors are colored green. Other genes present in BGC 34 are colored pink. Genes present in BGC 38 are colored orange. All other genes are colored gray.

**FIG 2:**
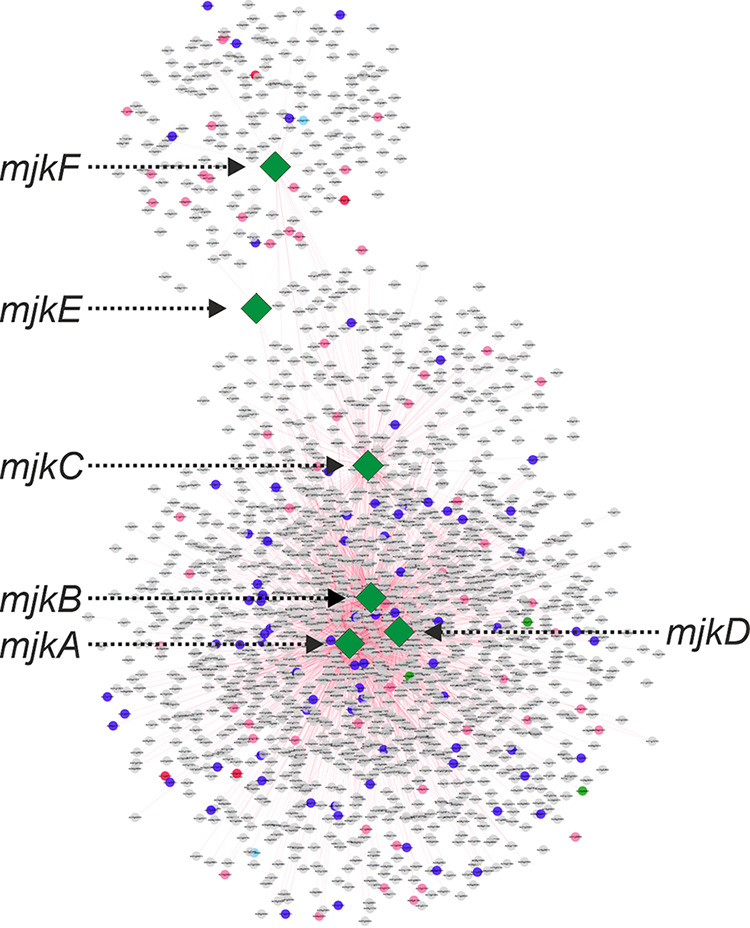
The largest Spearman subnetwork containing predicted BGC core and tailoring genes (highlighted in pink), as well as transcription factors (highlighted in blue). The six transcription factors studied by molecular analyses in this study (MjkA to -F) are indicated in green.

In total, 2,041 modules were recovered from the NET25 network, 2,944 modules from the NET10 network, and 2,999 modules from the NET05 network (Table S1A in the supplemental material). The median module sizes for the NET25, NET10, and NET05 networks were 11, 7, and 5 genes, respectively. Of the 78 predicted BGCs comprising, in total, 81 core genes in the A. niger genome, 43 predicted BGCs had one or more genes recovered within a single module (Table S1B). These 43 BGCs had various levels of coexpression, which we define here as being assigned to at least one shared module. For some BGCs, such as the fumonisin-producing BGC, most genes in the gene cluster are coexpressed at high levels (Fig. 1A). For others, either a small subset of the genes in the BGC were not coexpressed (e.g., BGC 34) (Fig. 1B) or only a small fraction of genes was coexpressed (e.g., BGC 38, where only 6 of 22 genes in the BGC were coexpressed) (Fig. 1C). During our analysis, we noticed that the two putative TF genes in BGC 34 were assigned to multiple overlapping modules due to the nature of the ClusterONE approach. Many of these modules also included genes from BGC 38. We collapsed all modules that contained these two predicted TF-encoding genes into one nonoverlapping metamodule that, notably, contained 7 genes from BGC 38 and 10 genes from BGC 34 (Fig. 1D). This metamodule consisted of 50 genes in total, including 1 core gene (fatty acid synthase [FAS]), 2 TFs from BGC 34 and 2 core genes (PKS and NRPS) from BGC 38. Concordantly, we could also identify coexpression between BGC 34 and BGC 38 cluster members via the SCC approach. Notably, MultiGeneBlast showed that BGC 34 and 38 are conserved in black aspergilli (Fig. S1). Both clusters belong to a large SCC subnetwork comprised of 1,804 genes (Fig. 2), which is the largest gene coexpression subnetwork with BGC genes based on the Spearman rank coefficient ρ ≥ |0.5|. This subnetwork included many TFs that are not physically located inside BGCs or are coexpressed with nonresident BGC genes.

**FIG 3:**
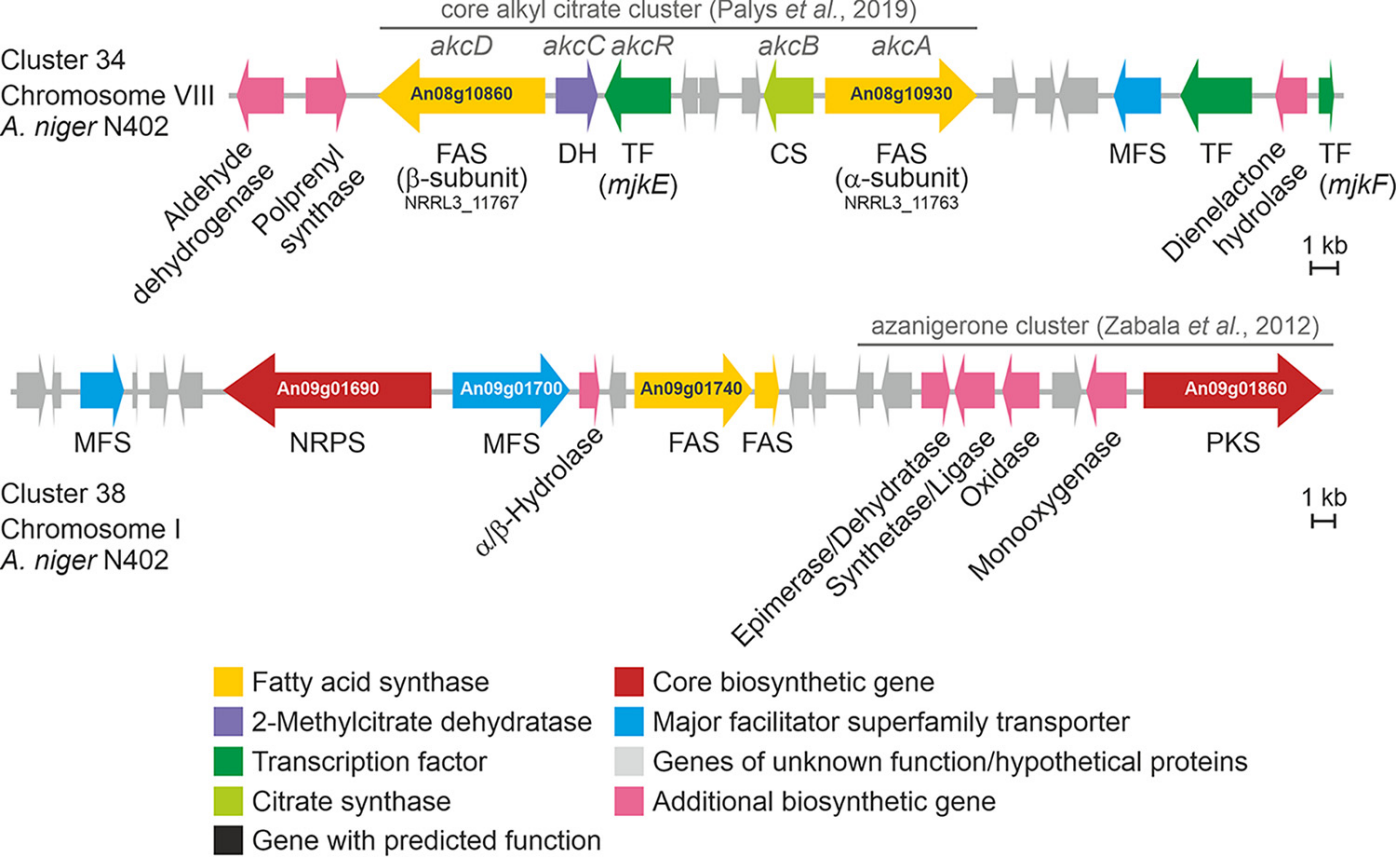
Schematic representation of BGC 34 and BGC 38 as predicted by antiSMASH. Based on sequence similarity and gene functional prediction, BGC 34 corresponds to the alkyl citrate-producing cluster identified in parallel to this study in A. niger NRRL3 (54). BGC 38 is positioned next to the azanigerone cluster (39).

It has been speculated over recent decades that BGC-resident TFs may coregulate gene expression at more than one BGC (1, 17). Both coexpression network approaches supported this hypothesis for A. niger, as evidenced by the coexpression of two TFs residing in BGC 34 (open reading frames [ORFs] An08g11000 and An08g10880, chromosome 1) with multiple genes in BGC 38 (chromosome 8), including the predicted NRPS (Fig. 3). This was especially interesting given that (i) BGC 38 does not contain a predicted TF, (ii) both these BGCs are present in 22 (BGC 34) or 24 (BGC 38) of 83 analyzed genomes of the genus Aspergillus, and (iii) BGC 38 is in close proximity to the functionally characterized BGC 39 that is necessary for azanigerone production (39).

Interestingly, our analysis demonstrates that the SCC approach primarily carves out coexpression of frequently expressed genes, whereas the strength of the MR-PCC approach is the identification of coexpression relationships among rarely expressed genes. We thus decided to study the impact of six putative TF-encoding genes on A. niger secondary metabolism in more depth. Four were predicted by the SCC approach to be coexpressed with at least 10 BGC core genes and are unclustered (MjkA to MjkD), whereas the remaining 2 were predicted by the MR-PCC approach to be coexpressed with both BGC 34 and BGC 38 and are clustered with BGC 34 (MjkE and MjkF) (Table 1 and Fig. 3).

### Overexpression of predicted transcription factors MjkA to -F modulates A. niger pigmentation and development.

**FIG 4:**
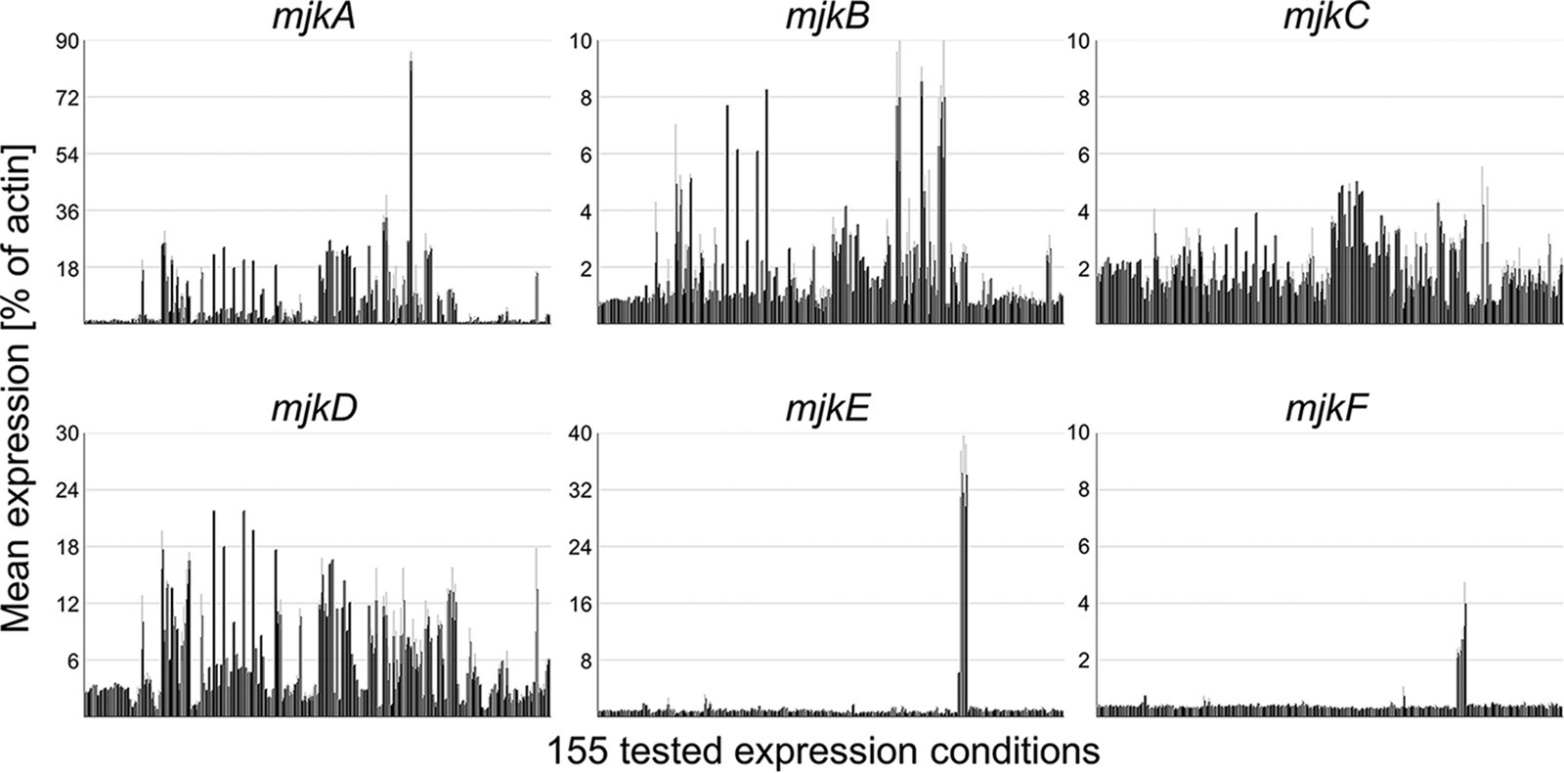
Expression levels for all 6 TFs under 155 expression conditions. Note the different scales. *mjkE* (An08g11000) and *mjkF* (An08g10880) expression levels are notably elevated during maltose-limited bioreactor growth in a Δ*flbA* mutant (77).

Prior to conducting gene functional analysis experiments, we assessed the gene expression profiles for *mjkA* to *mjkF* across our 155 cultivation conditions. While both *mjkE* and *mjkF*, which reside in BGC 34, were rarely expressed, the four genes *mjkA* to *mjkD* encoding unclustered TFs were transcribed under numerous conditions, with *mjkA* notably expressed to 90% of the level of A. niger actin under several conditions (Fig. 4).

To assess the role of these TFs in modulating BGC expression, we generated conditional expression isolates in which a Tet-On gene switch was placed upstream from the open reading frame as previously described for the genes *mjkA* and *mjkB* (33) (Fig. S2 presents cloning information and Southern blot confirmation). The Tet-On gene switch has undetectable levels of basal expression in the absence of induction, and the addition of 10 μg/ml doxycycline (DOX) enables expression above that of the A. niger glucoamylase gene, whose promoter is often used for overexpression studies (33, 40, 41). Conditional expression isolates previously constructed for genes *mjkA* and *mjkB* were also analyzed in this study to further assess their role in A. niger secondary metabolism and development (Table S1C).

**FIG 5:**
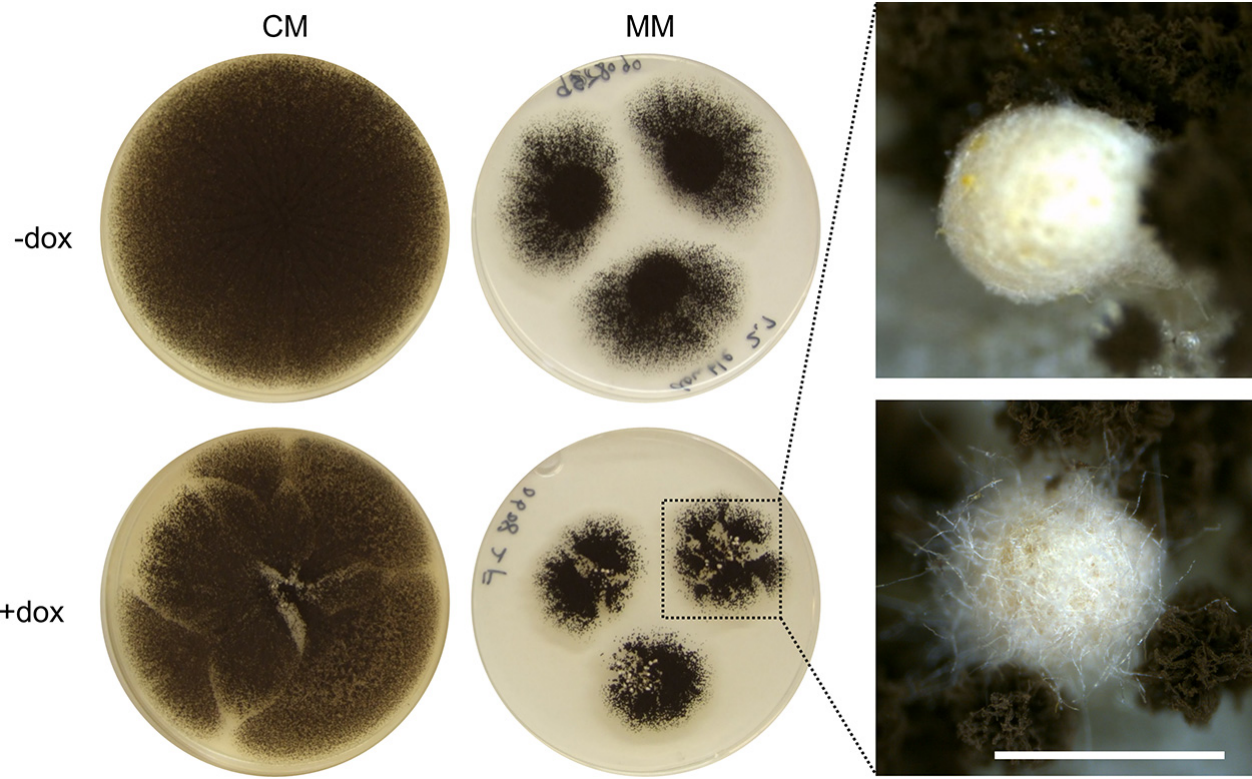
Tet-On-based overexpression of *mjkA* modifies A. niger development. Overexpression of *mjkA* induced by the addition of 10 μg/ml doxycycline leads to irregular formation of putative sclerotia on agar plates, an example of which is shown. Strains were grown on MM or CM for 144 h at 30°C in the dark. Colony sectoring observed in this isolate is not due to formation of unstable heterokaryons, as evidenced by PCR and Southern blot confirmation of homokaryotic strains. Estimated scale bar indicates approximately 1 mm.

**FIG 6:**
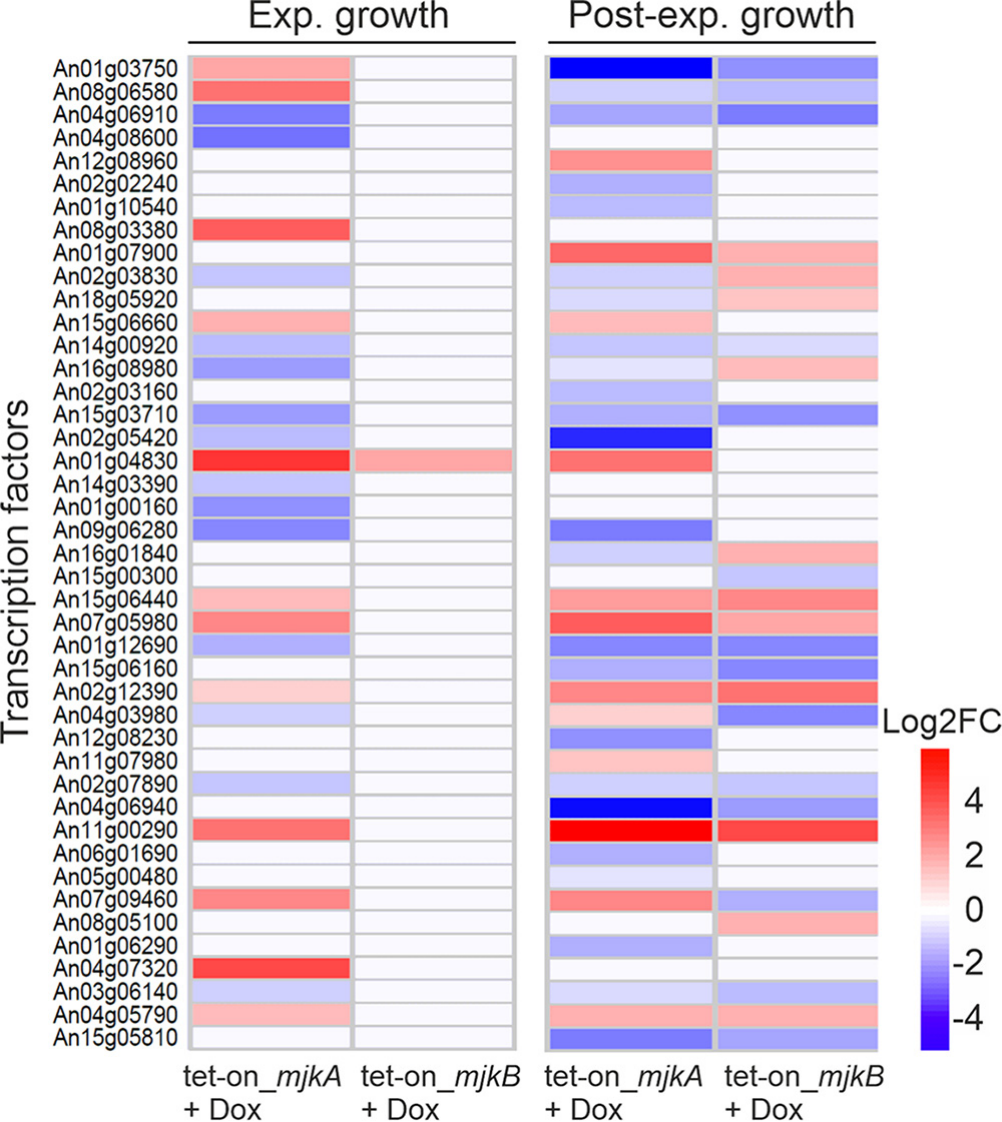
Differential gene expression of transcription factors following overexpression of *mjkA* and *mjkB* genes during controlled bioreactor batch cultivations of A. niger performed in our previous study (33). Note that overexpression of MjkA strongly affects expression of predicted regulators during both growth phases, whereas the effect of MjkB is limited to the post-exponential growth phase. ORF names are given.

Standard growth assays on solid and in liquid media clearly identified differences in medium pigmentation in overexpression isolates compared to the progenitor control (Fig. 5 and Fig. S3), suggesting a role of these genes in A. niger development and/or secondary metabolism. The MjkA, MjkD, and MjkF conditional expression strains also displayed reduced growth on solid agar under overexpression conditions (Fig. S3). Intriguingly, *mjkA* overexpression resulted in the infrequent formation of sclerotia (Fig. 5 and Fig. S3), which are an important prerequisite for sexual development in Aspergillus (42, 43). The observed putative sclerotia were highly similar in size, structure, and color to those recently characterized from A. niger (44). However, A. niger *sensu stricto* has not been reported to have a sexual cycle. Additionally, A. niger rarely produces sclerotia under specific growth conditions, which is paralleled by the production of many secondary metabolites, including indolterpenes of the aflavinine type (42). We thus reanalyzed transcriptomic data that were available for this isolate and for the MjkB overexpression strain from bioreactor cultivation (33) to screen for differential expression of developmental regulators following conditional MjkA and/or MjkB expression. Strikingly, the expression of 36 and 27 regulators and TFs was affected when *mjkA* or *mjkB*, respectively, was up- or downregulated (Fig. 6). Notably, the overexpression of *mjkA* resulted in downregulation of genes encoding transcription factors known to control primary metabolism (*creA*, *areB*, *xlnR*, *amyR*, *prtT*, *pacC*, *crzA*, *hapX*, *farA*, *farB*, and *acuB*) (45) and asexual development (*brlA*, *abaA*, *stuA*, *flbA*, *flbB*, and *flbC*) (45), as well as chromatin structure (*laeA*, *velB*, *vipC*, *mtfA*, and *hdaA*) (45), in Aspergillus (Fig. 6). Deletion of *mjkA* caused strong upregulation of the regulator-encoding genes *areA*, *cpcA*, *msnA*, *csnE*, *flbD*, and *vosA* (Table S1D), with functions in primary metabolism and development (45), implying that MjkA is a regulator of multiple A. niger BGCs, differentiation, and development. Note that the MjkA-encoding gene can be found in 61 of 83 sequenced Aspergillus genomes as identified by BLAST analyses (Table S2).

We further analyzed strains overexpressing genes *mjkA* to -*F* in shake flask cultures following induction with DOX by quantitative PCR (qPCR) (Table S1E). All genes displayed elevated transcription following the addition of DOX to the growth medium, with the exception of the *mjkD* overexpression strain, which we hypothesize could potentially be due to an autoregulation phenomenon (Table S1E) (46). Notably, qPCR analysis revealed that the overexpression of several putative TFs modified the expression of various known or predicted secondary metabolite-regulating genes, including *brlA*, *pacC*, and *mjkC* in the isolate overexpressing *mjkA*, and *mjkE* and *mjkF* in the isolate overexpressing *mjkC* (Table S1E). Furthermore, overexpression of *mjkF* caused marked upregulation of predicted core genes in cluster 34 (An08g10930 and An08g10860) and cluster 38 (An09g01690), suggesting that the *mjkF*-encoded protein may indeed regulate the expression of these clusters. Taken together, qPCR experimentation supported microarray-based coexpression analyses suggesting that genes *mjkA*, -*B*, -*C*, -*E*, and -*F* may regulate the expression of secondary metabolite gene expression.

### Overexpression of predicted transcription factors MjkA to -F modulates the secondary metabolite profile of A. niger.

**FIG 7:**
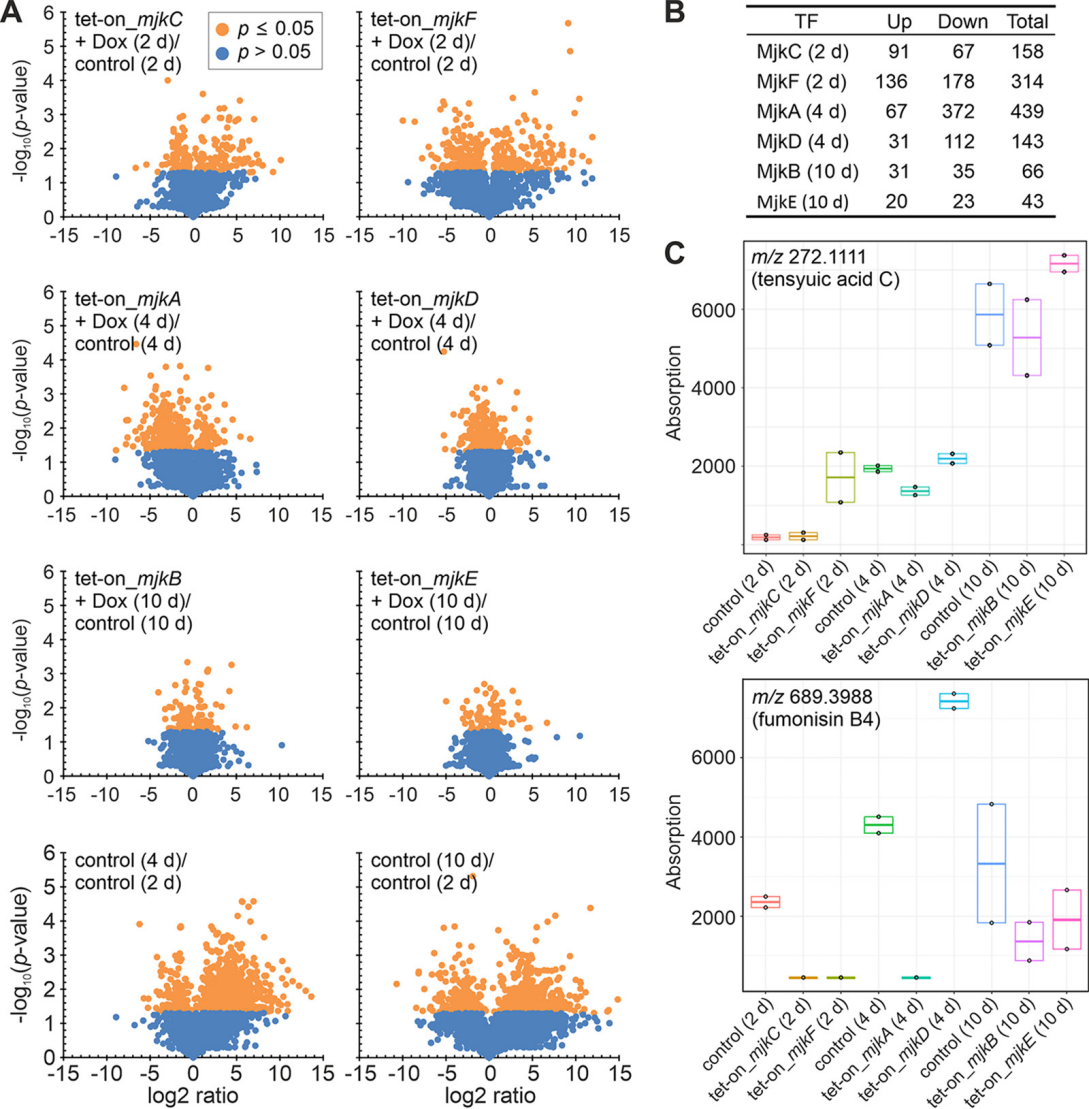
Overexpression of *mjkA* to *mjkF* genes affects numerous metabolites in A. niger. (A) Annotated metabolites were plotted by significance (*P* value) versus fold change (log_2_ ratio). Metabolites reaching a *P* value of <0.05 are marked orange. Metabolites with a *P* value of <0.05 and a log_2_ ratio greater than 1 or −1 were considered significant. (B) Numbers of significantly affected metabolites (*P* value of <0.05 and log_2_ ratio greater than 1 or −1) in comparison to their expression in the control strain. (C) Exemplary visualization of tensyuic acid C (alkyl citrate) and fumonisin B4 abundances during cultivation of overexpression and control strains of A. niger on agar plates at different time points (biological duplicates).

To understand the effects of the MjkA to -F TFs on the secondary metabolite profile of A. niger, we next conducted untargeted metabolome analysis of the progenitor strain and *mjkA* to *mjkF* conditional expression strains after 2, 4, or 10 days of incubation on minimal agar plates supplemented with 10 μg/ml DOX (Fig. S3). For each overexpression strain, a single time point was selected for metabolome analysis. Time points were chosen when the greatest deviation in either medium pigmentation or growth relative to that of the control strain was observed (Fig. S3). Since culture samples were harvested at both the center and the outer edges of the growing colonies and pooled for analysis, the results obtained comprise metabolites from both old and young mycelia. This analysis detected a total of 2,063 compounds, of which 1,835 were annotated. Metabolic pathway visualization of the identified metabolites using iPATH showed that intermediates from various biosynthetic routes toward SMs (Fig. S5, S6, and S7) were covered (47–49). Statistical analysis (*t* test) identified numerous metabolites that were significantly different (*P ≤ *0.05 and log_2_ ratio greater than 1 or −1) for the genotypes and time points compared (Fig. 7A).

Generally, overexpression of *mjkC* and *mjkF* (2 days) and of *mjkA* and *mjkD* (4 days) each affected more than 140 metabolites compared to the levels in the control strain at the respective time point (Fig. 7B). Interestingly, only overexpression of *mjkC* led to an upregulation of more than half of the affected metabolites, whereas overexpression of *mjkA*, *mjkD*, and *mjkF* led to downregulation relative to the levels in the control (Fig. 7B). In comparison, overexpression of *mjkB* and *mjkE* (10 days) apparently affected fewer metabolites (66 and 43, respectively), which might also be due to a reduced overall metabolic activity of the cultures after prolonged cultivation.

Among the significantly affected metabolites, several known SMs of A. niger and related species (50) could be putatively identified by means of liquid chromatography-quadrupole time of flight high-resolution mass spectrometry (LC-QTOF-HRMS), based on mass and retention time (Fig. 7C and Fig. S5 to S7). These compounds comprise naphtho-γ-pyrones (aurasperones, isonigerone, fonsecin, and carbonarins), bicoumarins (bicoumanigrin, kotanin, desmethylkotanin, and funalenone), and fumonisins. Moreover, overexpression of the putative TFs affected meroterpenoids (1-hydroxyyanuthone A) and benzoquinone-type pigments (atromentin and cycloleucomelone), as well as different types of alkaloids, such as pyranonigrins, pyrophens (aspernigrin A, carbonarone A, and nygerone A), nigragillins (nigragillin and nigerazine B), and tensidols. Not found among the significantly affected compounds were some known SMs of A. niger that have already been linked to their corresponding BGCs, such as azanigerone (39), TAN-1612 (51), and ochratoxin (52).

Notably, the list of previously identified SMs of A. niger is almost exclusively comprised of polyketide products (Fig. S6). Thus, even though the peptide-forming NRPS from BGC 38 (An09g01690) is present in a mutual rank metamodule with MjkE and MjkF, the biosynthetic product of BGC 38 is unlikely to be one of the compounds identified in the current study. Based on an *in silico* assembly line prediction using antiSMASH, An09g01690 encodes a bimodular NRPS that cannot be classified yet into a linear or iterative assembly type, and its product is thus not predictable. Since it is coexpressed with two putative fatty acid synthase-encoding genes (An09g01740 and An09g01750) in BGC 38 (Fig. 1 and 3), the encoded peptide presumably features a fatty acid moiety of various lengths based on the available fatty acid pool of A. niger. Similar patterns have been observed for other nonribosomally synthesized lipopeptides, such as daptomycin (53).

In parallel to this study, BGC 34 (Fig. 3) was recently demonstrated to be responsible for alkyl citrate production in A. niger strain NRRL3 (54). For this SM class, a range of bioactivities has been reported, including antiparasitic (55), antifungal (56), antibacterial (57), and plant root growth promotion effects (58). Other complex alkyl citrates (zaragozic acids, also called squalestatins) have been shown to be among the most potent natural squalene synthase inhibitors (59, 60). Notably, the metabolome analysis in this study showed that several alkyl citrates, such as hexylaconitic acid A, hexylitaconic acid J, and tensyuic acids C and E, were also differentially produced at different time points upon TF overexpression (Fig. 7C and Fig. S5 to S7).

## DISCUSSION

This study has demonstrated that gene coexpression analysis enables the identification of fungal transcriptional networks in which secondary metabolite genes are embedded. By comparing mutual rank and Spearman-derived coexpression networks, we have identified, respectively, both BGC-resident and, additionally, unclustered TFs, a finding that is broadly consistent with the existence of SM regulatory genes that reside outside predicted BGC loci (17). However, there is a growing body of evidence to suggest that, at least in some instances, there has been an overreliance on physical clustering for the prediction of SM pathway genes and their cognate transporters/regulators. Indeed, with several notable exceptions (61, 62), it is still relatively rare that genes required for the biosynthesis of an entire fungal SM are, first, experimentally verified and, second, fully contiguously clustered. Thus, the true extent of SM pathway gene clustering in fungi remains unclear. This is further complicated by divergence in the degrees to which the BGCs are intact across fungal genomes, which is even true for “gold standard” BGCs, such as those necessary for epipolythiodioxopiperazine synthesis (e.g., gliotoxin/sirodesmin) (61). Hence, experimental approaches to activate and functionally analyze the full fungal SM repertoire cannot exclusively rely on *in silico* genomics approaches.

Given that coexpression approaches have only recently been applied to define fungal BGC boundaries and their transcriptional networks (29, 31, 33, 63), in this study, we examined the potential utility of two different approaches for constructing coexpression networks, namely, mutual rank and Spearman approaches. Our results suggest that both approaches enable the delineation and refinement of contiguous BGC boundaries. However, whereas the Spearman approach was better suited for the identification of global TFs, the mutual rank approach was better suited for the identification of pathway-specific TFs. This work should therefore guide future coexpression analyses of other fungal transcriptional data sets based on the requirements of the end user (i.e., global or pathway-specific studies). Regarding the minimal number of transcriptomic data sets necessary to generate useful coexpression resources, we argue that, given the variation between BGC expression levels between fungi, such estimations are not currently possible. However, we advocate that, ideally, interested users should (i) utilize a maximum amount of transcriptomic data from high-quality public databases, (ii) select and generate transcriptomic data from conditions known or predicted to activate BGC expression for the BGC of interest, or (iii) use a combination of these approaches.

Overexpression of six TF-encoding genes (*mjkA* to -*F*) predicted from coexpression networks to be involved in A. niger SM regulation enabled the modification of A. niger secondary metabolite profiles, which included the production of SMs that were not detected in the progenitor control (Fig. S7). Thus, wholesale modulation of fungal SMs in standard laboratory culture is possible using hypotheses derived from both Spearman and mutual rank network approaches. The simplicity of the culture conditions is an attractive aspect of the discovery pipeline in this work, which may be preferable to more complex experimental setups, such as cocultivation experiments or isolation of novel metabolites from the complex fungal niche (e.g., soil) or marine environments (64).

From a methodological perspective, our data support the notion that TF overexpression using an inducible gene switch is an effective strategy for SM activation and is probably preferable to conventional gene deletion approaches (33). It should be noted, however, that this study was clearly not able to activate all A. niger SMs, as we only analyzed SM profiles from a single growth stage/time point for each mutant. Therefore, we speculate that activation of other metabolites will be observed under different culture conditions or at different growth phases. Consequently, the full exploration of the SM repertoire of A. niger MjkA to -F isolates will be conducted in follow-up studies. It should be noted that it is currently unclear whether the *mjkA* to *-F* genes will enable the activation of BGCs at levels comparable to the levels induced by existing regulators, such as *laeA*, *pacC*, *areA*, *creA*, *stuA*, or *brlA*. Where conservation of MjkA to -F is observed in other fungal genomes, the functional analysis (i.e., overexpression) of such orthologues to activate and discover other SM molecules appears feasible.

An exciting observation during this study was the irregular formation of putative sclerotia due to overexpression of MjkA, which can be viewed as a preliminary (and tentative) step toward laboratory-controlled sex, opening up the possibility of classical genetics in this species (65). Such developmental jackpots may be viewed as an additional benefit to wholesale analysis of fungal SMs using coexpression networks.

In this work, we also conducted significant *in silico* and mass spectrometry-based characterization of differential SM production profiles and attempted to link empirically observed SMs to specific BGCs. Despite recent advances in publicly available tools for such experiments, including the prediction of putative SM structures based on the analysis of PKS/NRPS domains (66), coupling BGCs to their products is still challenging. In this respect, linking BGCs among multiple differentially produced SMs between control and experimental cohorts remains a significant bottleneck in discovery pipelines and requires experimental validation of putative BGC metabolite candidates, e.g., by means of core gene knockout or overexpression.

In summary, this study has generated novel coexpression resources and methods for the microbial cell factory A. niger. Overexpression strains MjkA to -F are promising tools for metabolite discovery and will be used in future to reverse engineer the transcriptional networks to which they belong. Our data clearly support the well-established prevalence of BGCs in filamentous fungal genomes but suggest a refinement to this paradigm, whereby for activation and functional analysis experiments of SMs, it may be safer to consider that the necessary genes for a fungal SM of interest (including core genes, tailoring genes, transporters, detoxifiers, and regulators) may be unclustered but can be identified by means of both SCC and MR-PCC coexpression analyses. Such shifts in experimental thinking may help facilitate the full exploitation and comprehensive understanding of SMs among the fungal kingdom.

## MATERIALS AND METHODS

### Calculating mutual rank for microarray experiments.

A. niger microarray data sets obtained across a range of experimental conditions and genetic backgrounds (33) were analyzed in R using the affy, simpleaffy, and makecdfenv packages (67–69). Raw data from each of the 283 individual microarrays were normalized using the robust multiarray average (RMA) expression method as implemented in the affy package (67). To enable cross-experiment comparisons, expression values were normalized by scaling to the cross-experiment trimmed mean (excluding the top and bottom 5% of expression values). Pearson’s correlation coefficient was calculated between every pair of genes across all conditions. An ordered list of all genes from most to least correlated was generated for each gene. For every pair of genes, the mutual rank was calculated by taking the geometric mean of the rank of each gene in the other gene’s ordered list. The mutual rank (MR) of two genes A and B is the geometric mean of each gene’s correlation rank and is given by the following formula:

$${\text{MutualRank}}_{A,B}=\sqrt{{\text{Rank}}_{A(B)}\times {\text{Rank}}_{B(A)}}$$

where Rank*_A_*_(_*_B_*_)_ is the rank of gene B in an ordered list of the correlation coefficients of all genes with respect to gene A ranked from most to least correlated (34). MR scores were transformed to network edge weights using the exponential decay function *e*^−(MR − 1/^*^x^*^)^; three different networks were constructed with *x* set to 5, 10, and 25, respectively. Edges with a Pearson’s correlation coefficient of <0.3 or an edge weight of <0.1 were excluded from the global network, which was then visualized in Cytoscape (70). Modules of coexpressed genes were inferred using ClusterONE with default parameters (37). Modules were analyzed for the presence of transcription factors and for SM backbone genes based on protein domains found within these genes and from gene annotations predicted by antiSMASH (71). For two transcription factor genes (*mjkE* and *mjkF*), the results from all coexpression networks were combined by collapsing all modules containing these genes of interest into a metamodule of nonoverlapping genes. For identification of shared clusters in Aspergillus species (Table S2), MultiGeneBlast (72) was used with 83 available representative genome assemblies available on NCBI Assembly as the search database (Table S1F).

### Strains and molecular techniques.

The A. niger strains used in this study are summarized in Table S1C. Medium compositions and the methods for transformation of A. niger, strain purification, and fungal chromosomal DNA isolation were as previously described (73). Standard PCR and cloning procedures were used for the generation of all constructs (74), and all cloned fragments were confirmed by DNA sequencing. Correct integrations of constructs in A. niger were verified by Southern blot analysis (74). For overexpressing *mjkC*, *mjkD*, *MjkE*, and *mjkF*, the respective open reading frames were cloned into the Tet-On vector pVG2.2 (40) and the resulting plasmids integrated as single or multiple copies at the *pyrG* locus of strain MA169.4. The expression of the respective Tet-On-controlled gene was measured 24 h after DOX induction in all overexpression mutants, using qPCR (Table S1E). Details on cloning protocols, primers used, and Southern blot results are available upon request from the authors.

### Growth assays.

All A. niger isolates were routinely cultured in the dark at 30°C in either minimal medium (MM) (75) or complete medium (CM), which consisted of MM supplemented with 0.5% Casamino Acids and 1% yeast extract as described previously (75). Doxycycline (DOX) was added to either solid or liquid medium where indicated to a final concentration of 10 μg/ml. For growth assays on solid medium, 10^5^ spores were inoculated on CM or MM with or without DOX and grown for up to 144 h. For A. niger cultivations in liquid medium, spores were inoculated into 50 ml of MM at a concentration of 10^6^/ml. Cultures were incubated at 30°C and 200 rpm. DOX was added 16 h after inoculation, which approximates the exponential growth phase. DOX was then added every 24 h until a maximum period of 92 h. Strain MJK17.25 served as the control strain for all growth assays (Table S1C). For qPCR, 50 ml Aspergillus minimal medium supplemented with 0.1% yeast extract to enhance spore germination was inoculated at a concentration of 5 × 10^6^ spores/ml into 250-ml shake flasks. Technical duplicates were prepared for all overexpression strains, and a quadruplicate cultivation was performed for the parental strain. Flasks were shaken at 250 rpm and 30°C for 12 h to allow spore germination before the addition of 20 μg/ml doxycycline to induce the expression of genes under the control of the Tet-On system. The parental strain was treated identically. Samples for qPCR were taken 24 h after induction by separating broth and mycelium via vacuum filtration, briefly washing with Milli-Q water, and sampling 100 to 200 mg mycelium into screw-cap tubes with sterile glass beads and 1 ml TRIzol reagent. Until total RNA extraction, samples were stored at −80°C.

### RNA isolation and qPCR.

For isolation of total RNA, samples were thawed and homogenized using a FastPrep 120 (Thermo Fisher Savant) and processed further using a Direct-zol RNA miniprep kit (Zymo Research). Total RNA was quantified using a BioSpectrometer (Eppendorf), and 2 μg of total RNA was used for reverse transcription in a 20-μl reaction mixture with random hexameric primers following the instructions of the RevertAid H minus first-strand cDNA synthesis kit (Thermo Fisher). Reverse transcription reaction mixtures were diluted 12-fold with nuclease-free water, and 2 μl of the dilution was used as the input for a 10-μl SYBR-based qPCR (Blue S′Green qPCR kit; Biozym Scientific) on an AriaMx real-time PCR system (Agilent). Primers are listed in Table S1E. For each sample, technical duplicates for each target gene were measured. Raw threshold cycle (*C_T_*) data were exported to MS Excel and the Δ(*C_T_*) values from technical replicates (shake flasks and reaction replicates) were averaged before calculating the ΔΔ(*C_T_*) values according to the method in reference 76, assuming an amplification efficiency of one. Actin (An15g00560) was used as the reference gene.

### Metabolome profiling.

Metabolites were extracted from colonies of A. niger MJK17.25 grown on agar plates (independent biological duplicates) by Metabolon (Potsdam, Germany). In brief, three agar plugs (outer edge to plate, center of colony, and outer edge adjacent to next colony) were collected at different time points from a colony cultivated for 2 to 10 days on minimal agar medium and pooled in one reaction tube. Each sample was extracted in a concentration of 0.5 g/ml with isopropanol:ethyl acetate (1:3, vol/vol) by ultrasound for 60 min and centrifuged at 4°C at 13,500 rpm for 20 min. The supernatant was sterile filtrated (0.22 μm; Carl Roth) and transferred into a new Eppendorf tube. All subsequent steps were carried out at Metabolon (Potsdam, Germany). Metabolites were identified in comparison to Metabolon’s database entries of authentic standards. The LC separation was performed using hydrophilic interaction chromatography with an iHILIC-Fusion, 150- by 2.1-mm, 5-μm, 200-Å column (Hilicon, Umeå Sweden), operated by an Agilent 1290 ultraperformance liquid chromatography (UPLC) system (Agilent, Santa Clara, CA, USA).

The LC mobile phase A was 10 mM ammonium acetate (Sigma-Aldrich, USA) in water (Thermo Fisher, USA) with 95% acetonitrile (pH 6; Thermo Fisher, USA), and mobile phase B was acetonitrile with 5% 10 mM ammonium acetate in 95% water. The LC mobile phase was a linear gradient from 95% to 65% acetonitrile over 8.5 min, followed by a linear gradient from 65% to 5% acetonitrile over 1 min and then a 2.5-min wash with 5% and a 3-min reequilibration with 95% acetonitrile (flow rate, 400 μl/min). Mass spectrometry was performed using a high-resolution 6540 QTOF/MS detector (Agilent, Santa Clara, CA, USA). Spectra were recorded in a mass range from 50 *m/z* to 1,700 *m/z* in positive and negative ionization mode. The measured metabolite concentration was normalized to the internal standard. Significant concentration changes of metabolites in different samples were analyzed by appropriate statistical test procedures (Student test, Welch test, and Mann-Whitney test). A *P* value of <0.05 was considered significant.
