# Supplementary material for: Beyond the Biosynthetic Gene Cluster Paradigm: Genome-Wide Coexpression Networks Connect Clustered and Unclustered Transcription Factors to Secondary Metabolic Pathways
Source: Microbiol Spectr. 2021 Sep 15;9(2):e00898-21. doi: 10.1128/Spectrum.00898-21 (PMC8557879; doi:10.1128/Spectrum.00898-21)
Supplement: SUPPLEMENTAL FILE 1 — Supplemental material. Download SPECTRUM00898-21_Supp_1_seq10.pdf, PDF file, 1.7 MB [file spectrum00898-21_supp_1_seq10.pdf]

Supplemental Tables (excel files)

Supplemental Table 1: (A) All co-expression modules called using ClusterONE. (B) List of all co-expression modules containing BGC core genes that overlap with two or more clustered genes. (C) Strains used in this study. (D) Differential expression of selected genes amongst conditional expression isolates described in this study. (E) qPCR assessment of various genes in overexpression mutants. (F) Aspergillus species used for MultiGeneBlast database generation.

Supplemental Table 2: MjkA homologs amongst identified by BLAST analysis.

Supplemental Figure 1: MultiGeneBLAST of BGC 34 and 38 members reveals several orthologous clusters in related *Aspergilli*. Cluster members for the query were chosen based on the co-expression heatmap of BGC 34 (An08g10830 - An08g11020) and BGC 38 (An09g01610 - An09g01880), respectively.

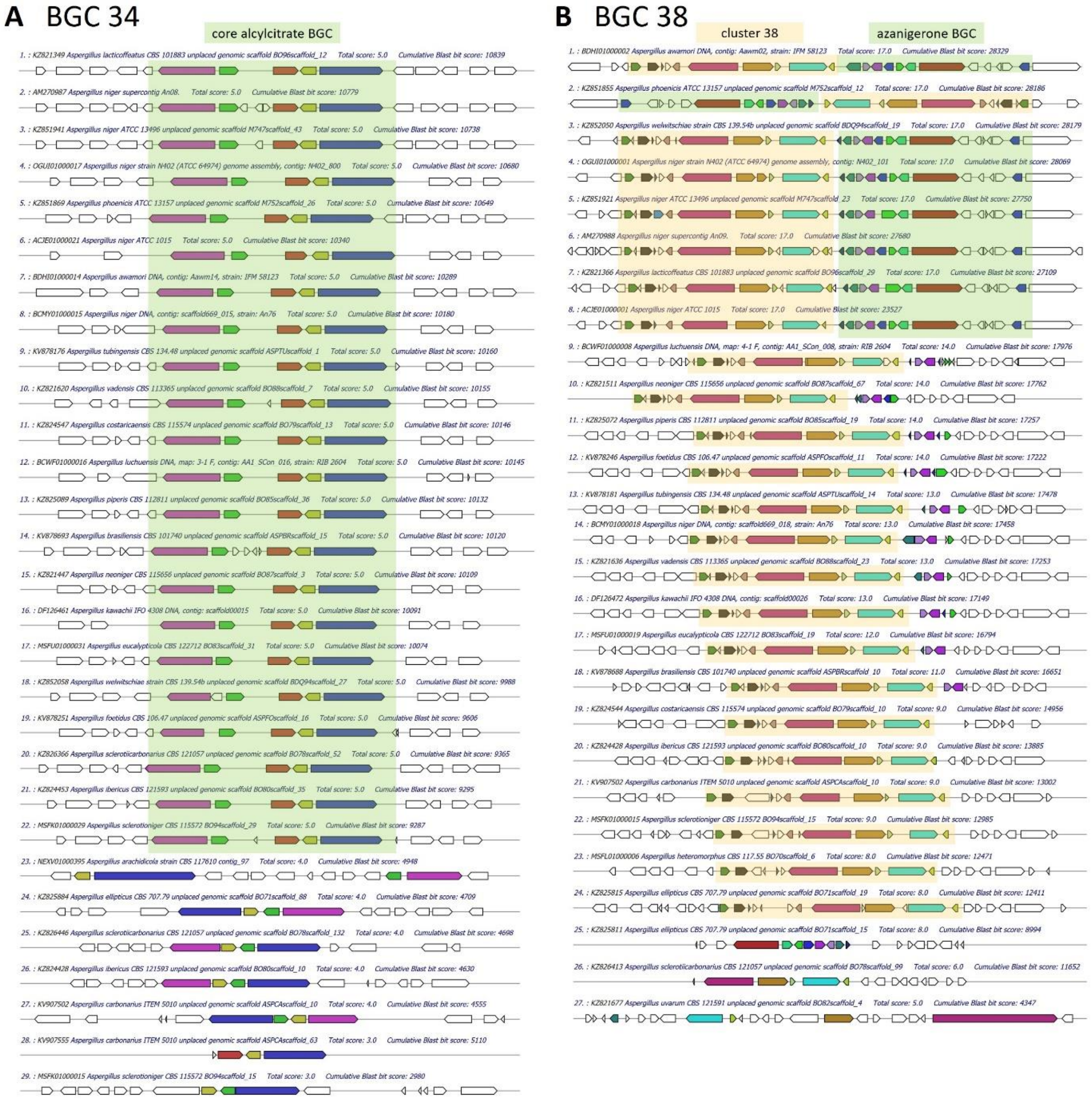

Supplemental Figure 2: (A) Schematic representation of *pyrG* targeting expression cassettes. All over expression constructs were generated via aqua cloning using PCR amplified ORF fragment containing homologous overhangs with PmeI digested vector pVG4.1. In this instance gene *mjkC* is given as an exemplar. (B) Schematic diagram depicting restriction endonuclease digestion sites at the *pyrG* locus in progenitor control and mutants generated in this study. (C) Two Southern blots used to confirm single integration events of the cassette at the *pyrG* locus in PCR confirmed recipient genomes for isolates used in this study. WT indicates control strain. Isolates depicted with a red tick pass Southern blot quality control for single integration events. Note that strains and restriction enzymes not used in this study are depicted in the blot.

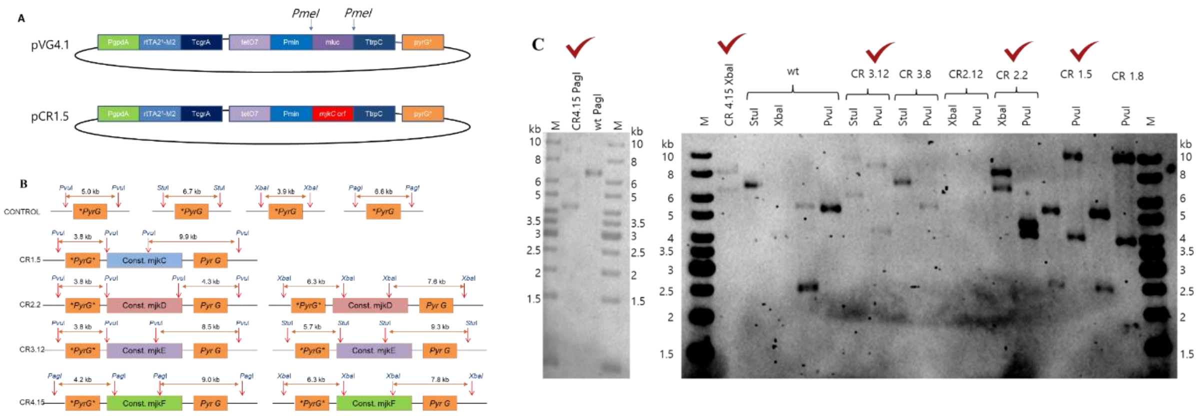

Supplemental Figure 3: *A. niger* growth phenotypes. (A) Phenotypes and sampling time points for metabolome analysis. Note that extensive phenotypic profiling was conducted, and data will be made available on request. (B) Overexpression of *mjkA* induced by the addition of 10 µg/ml doxycycline leads to irregular formation of putative sclerotia on agar plates, an example of which is shown. Strains were grown on MM for 144 hours at 30 °C in the dark. (C) Variations in culture and supernatant pigmentation in isolate MJK17.25 (control) and isolates Tet-on_*mjkA-F.* Strains we incubated as described in the Materials and Methods section in MM for 92 hours at 30°C with 200 RPM in the dark. Media was supplemented with 10 µg/ml Dox, and representative images from duplicate cultures are shown. Scale bar in bottom panel = 0.5 cm.

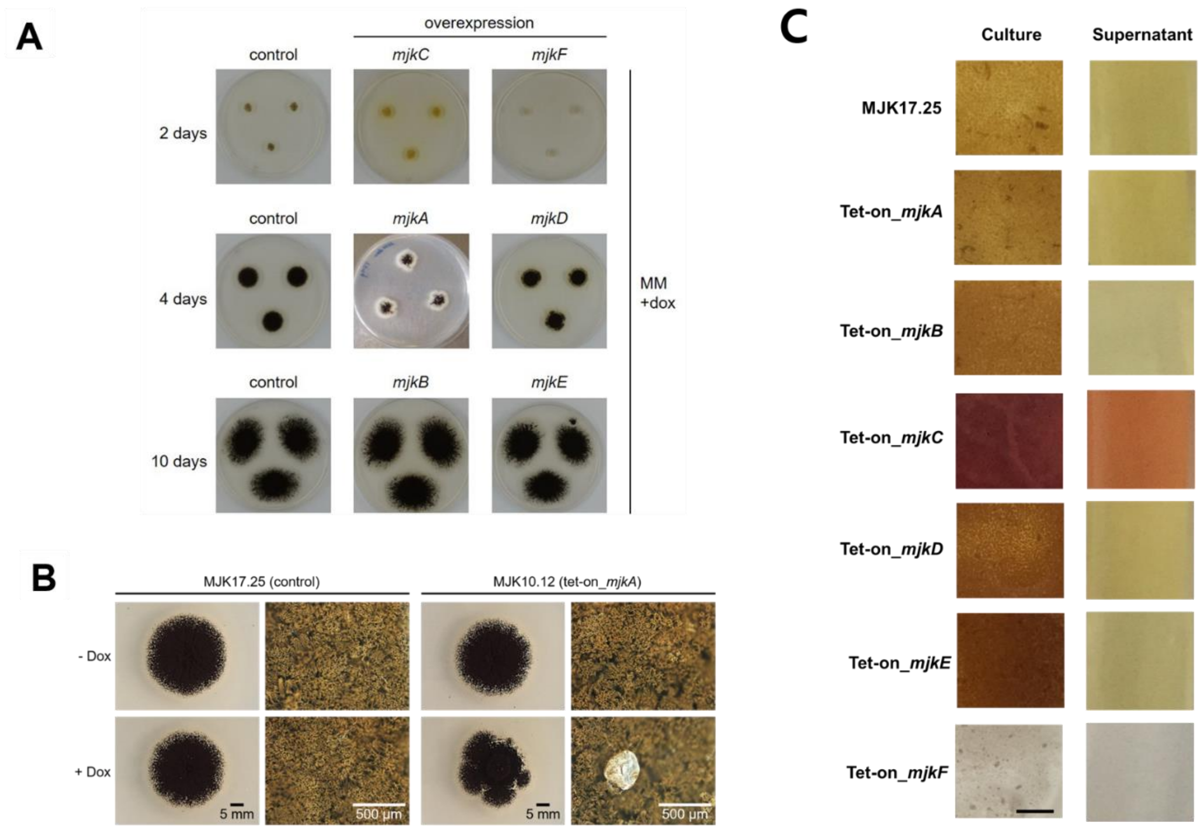

Supplemental Figure 4: Identified metabolites from the untargeted metabolome studies of *A. niger*. (A) Identified metabolites involved in SM biosynthesis. Metabolites were visualized on a metabolic pathway map depicting SM biosynthesis routes using iPATH. (B) Identified metabolites involved in antibiotics biosynthesis. Metabolites were visualized on a metabolic pathway map depicting biosynthesis routes leading to antibiotics using iPATH.

A

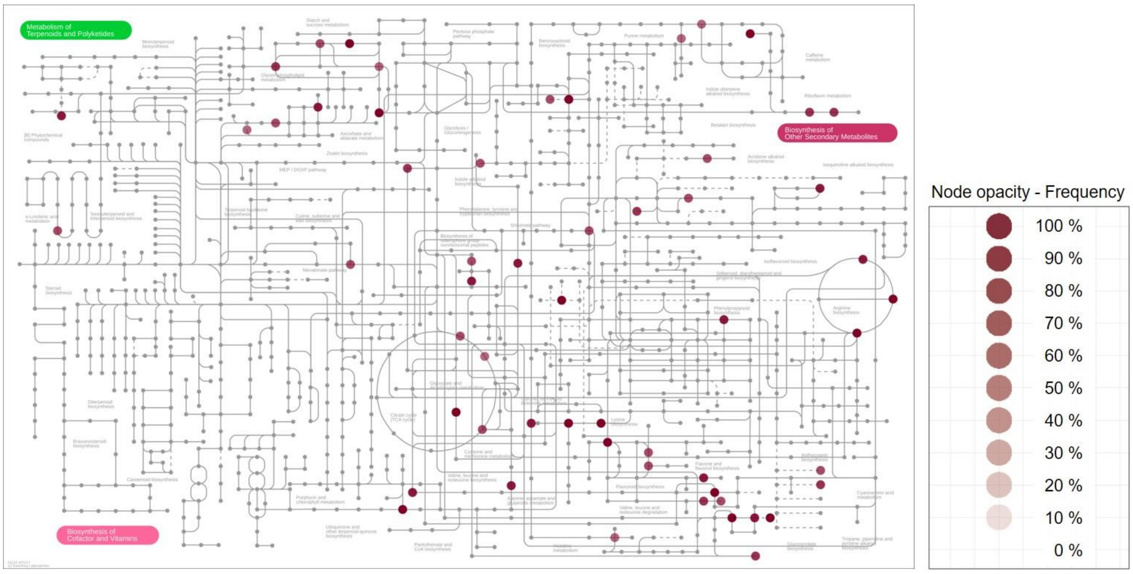

B

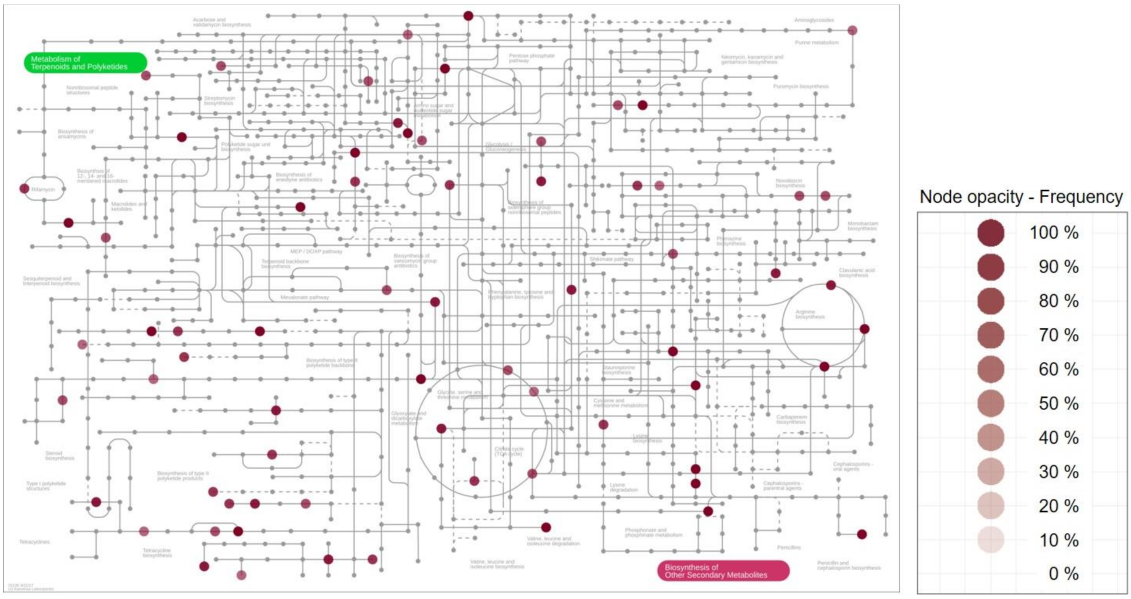

Supplemental Figure 5: Metabolite production overview for *A. niger* strains overexpressing *mjkA–F*. LC-MS total ion chromatograms are shown for the different mutants in comparison to the control strain MJK17.25 (black) over time (2, 4, 10 d) in solid cultivation. Measurements were performed in positive (top part) and negative ionization mode (bottom part), respectively. Retention times of known metabolites of *A. niger* (right part) were marked in the chromatograms (grey dotted lines). The gradient used for all LC-MS measurements is stated in the first chromatogram (blue line).

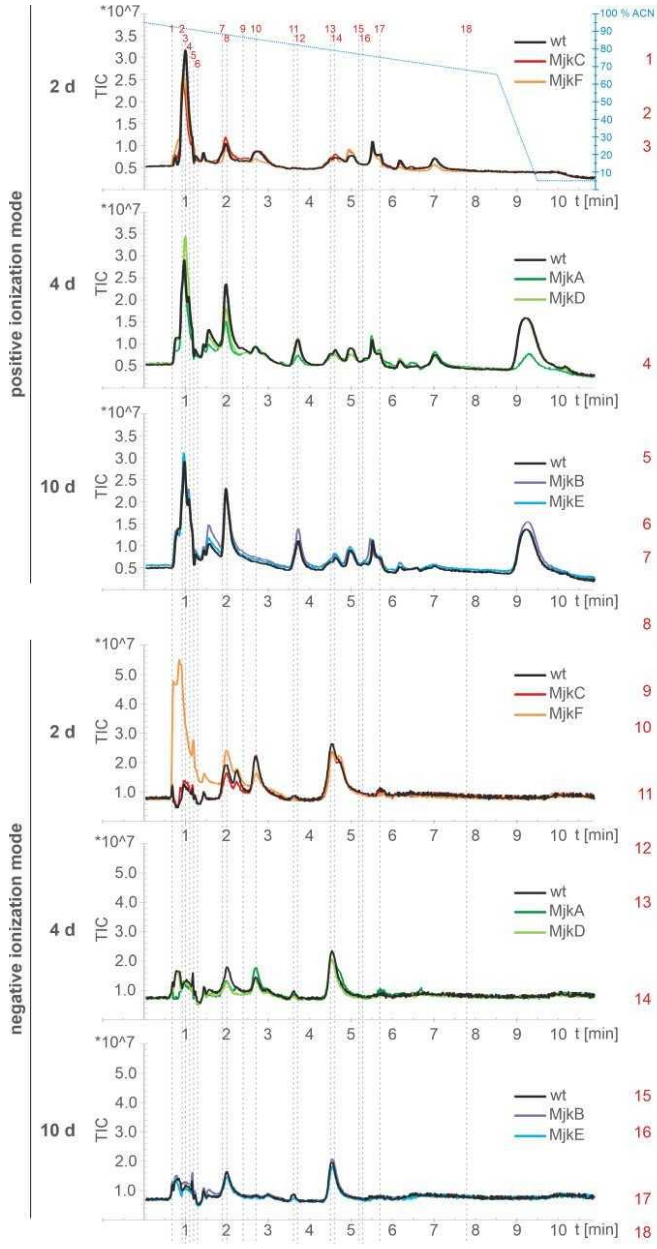

	Compound	*m/z*
1	Dianhydro-aurasperone C	556.1361
2	Atromentin	324.0625
3	Isonigerone	570.1528
	Aurasperone E	588.1640
	Kotanin	438.1326
	Aurasperone B	606.1750
	Demethylkotanin	424.1167
	Fumonisin B1	721.3812
	Aurasperone C	592.1581
4	Bicoumanigrin	410.1007
	Tensidol B	343.1066
	Fonsecin	290.0796
5	Carbonarone A	229.0722
	Cycloleucomelon	338.0437
6	Funalenone	288.0640
7	Carbonarin H	566.1411
	Nygerone A	418.1479
8	Carbonarin F	612.1658
	Tensyuic acid E	286.1572
9	Nigerazine B	258.1734
10	Nigragillin	222.1732
	Asnipyrone A	322.1628
11	Pyranonigrin A (pyranopyrrol A)	223.0483
12	1-Hydroxy-yanuthone A	400.2205
13	Fumonisin B4	689.3988
	Fumonisin B2	705.3938
	Glycolic acid	76.0169
14	Aspernigrin A	228.0852
	Carbonarin E	311.1220
	Tensyuic acid C	272.1111
15	Aspergillin	368.0464
16	Tensyuic acid	244.0992
	Aspernigrin B	456.1812
17	Carbonarin C	565.1616
18	Oxalic acid	89.9968

Supplemental Figure 6: Known SMs of *A. niger* and related species affected by overexpression of *mjkA* - *mjkF*. Structures of putatively identified SMs from metabolome analysis, which were significantly affected and have been previously described for *A. niger* and related species.

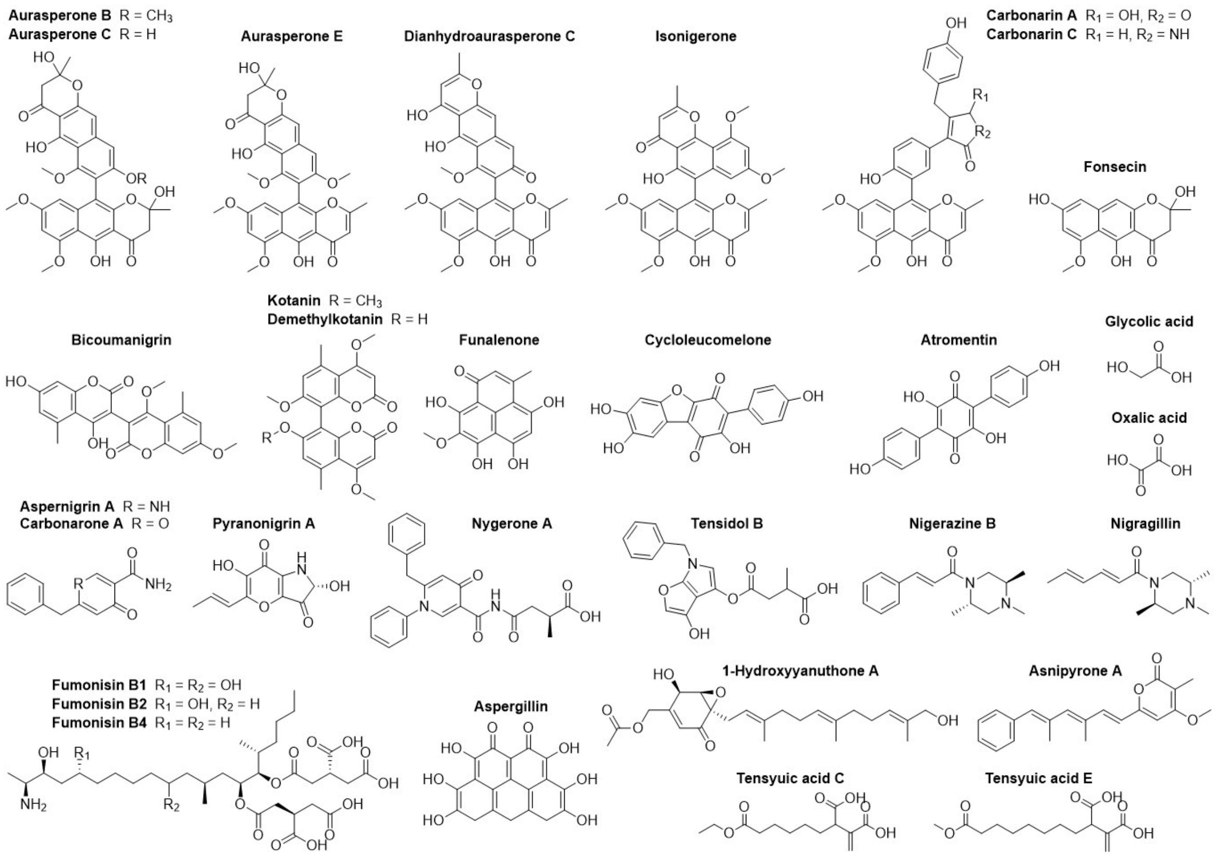

Supplemental Figure 7: Boxplot visualization of known SMs of *A. niger* identified from metabolome analysis. SM abundances were monitored during cultivation of overexpression and control strains of *A. niger* on agar plates at different time points (biological duplicates).

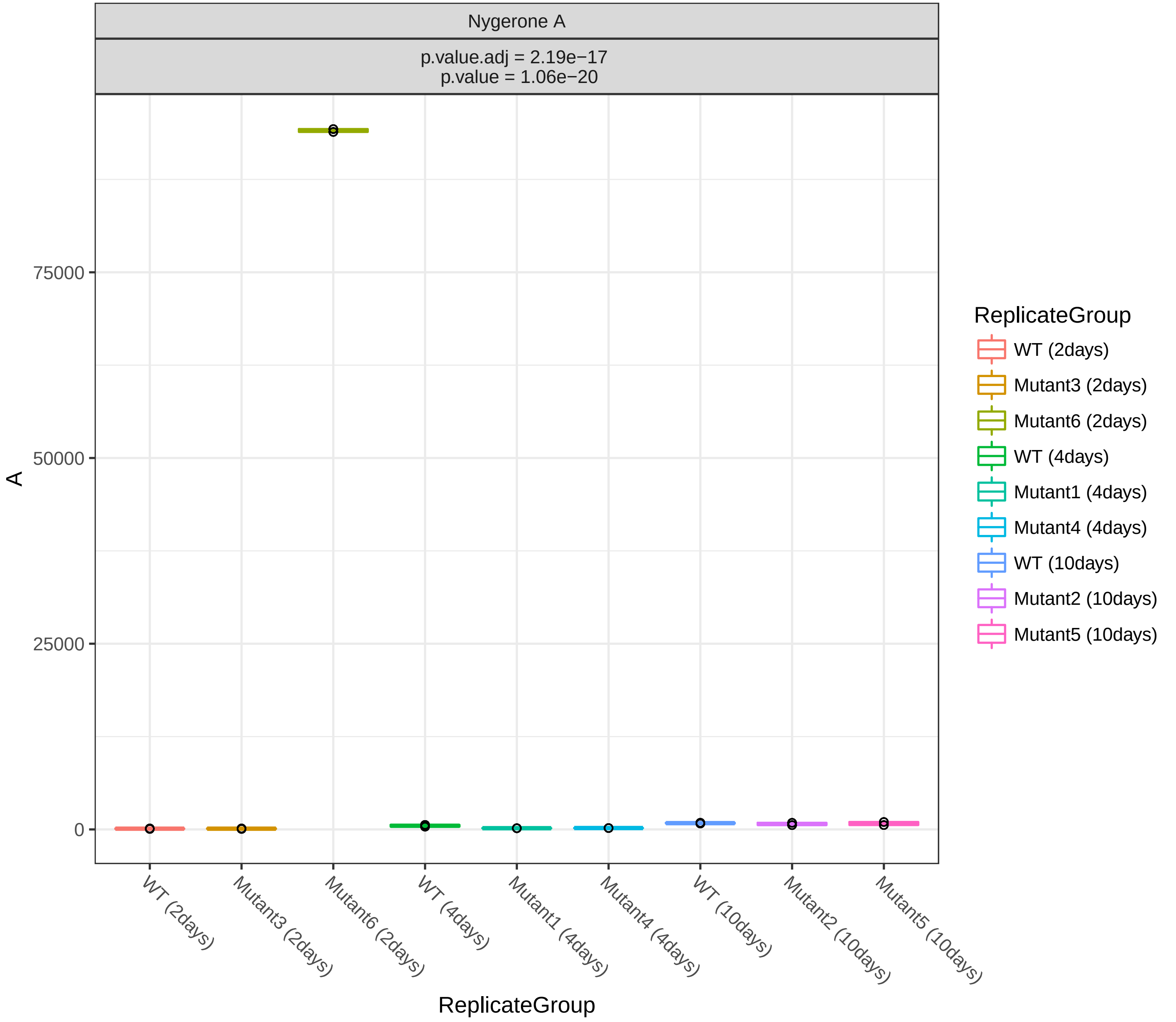

Nygerone A
p.value.adj = 2.19e−17
p.value = 1.06e−20
A
75000
50000
25000
0
WT (2days)
Mutant3 (2days)
Mutant6 (2days)
WT (4days)
Mutant1 (4days)
Mutant4 (4days)
WT (10days)
Mutant2 (10days)
Mutant5 (10days)
ReplicateGroup
ReplicateGroup
WT (2days)
Mutant3 (2days)
Mutant6 (2days)
WT (4days)
Mutant1 (4days)
Mutant4 (4days)
WT (10days)
Mutant2 (10days)
Mutant5 (10days)

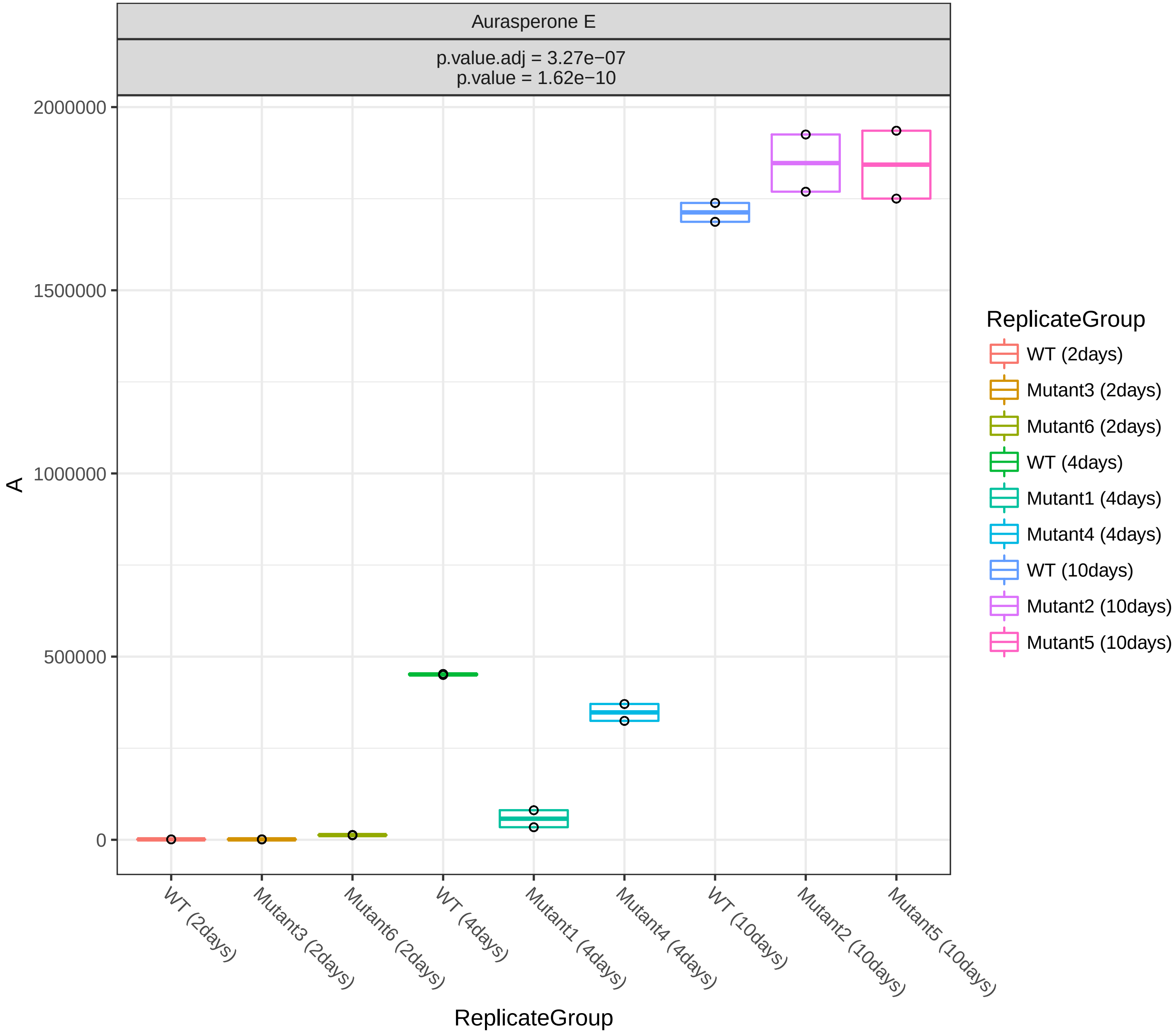

Aurasperone E
p.value.adj = 3.27e−07
p.value = 1.62e−10
A
2000000
1500000
1000000
500000
0
WT (2days)
Mutant3 (2days)
Mutant6 (2days)
WT (4days)
Mutant1 (4days)
Mutant4 (4days)
WT (10days)
Mutant2 (10days)
Mutant5 (10days)
ReplicateGroup
ReplicateGroup
WT (2days)
Mutant3 (2days)
Mutant6 (2days)
WT (4days)
Mutant1 (4days)
Mutant4 (4days)
WT (10days)
Mutant2 (10days)
Mutant5 (10days)

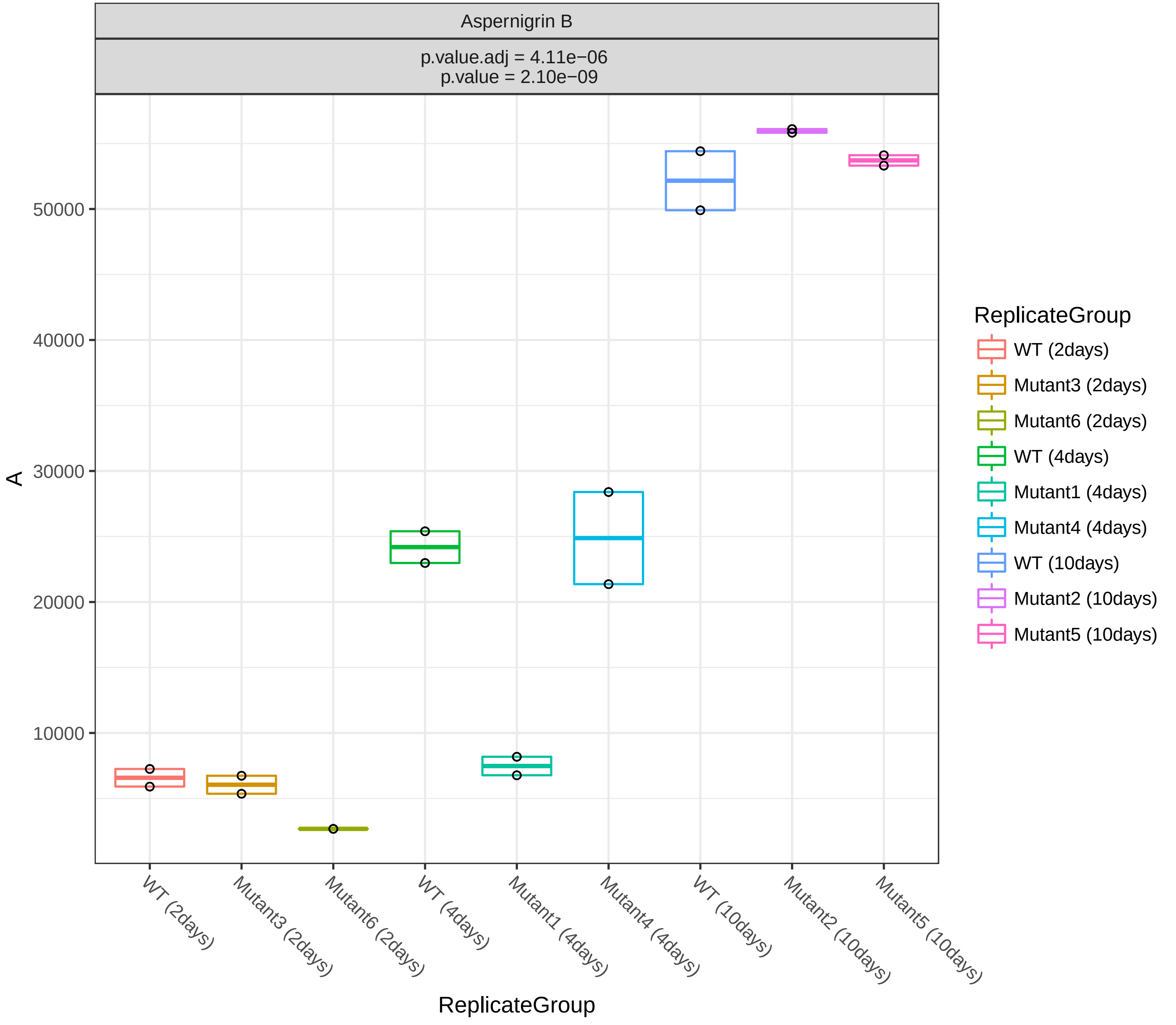
Aspernigrin B
p.value.adj = 4.11e−06
p.value = 2.10e−09
A
50000
40000
30000
20000
10000
WT (2days)
Mutant3 (2days)
Mutant6 (2days)
WT (4days)
Mutant1 (4days)
Mutant4 (4days)
WT (10days)
Mutant2 (10days)
Mutant5 (10days)
ReplicateGroup
ReplicateGroup
WT (2days)
Mutant3 (2days)
Mutant6 (2days)
WT (4days)
Mutant1 (4days)
Mutant4 (4days)
WT (10days)
Mutant2 (10days)
Mutant5 (10days)

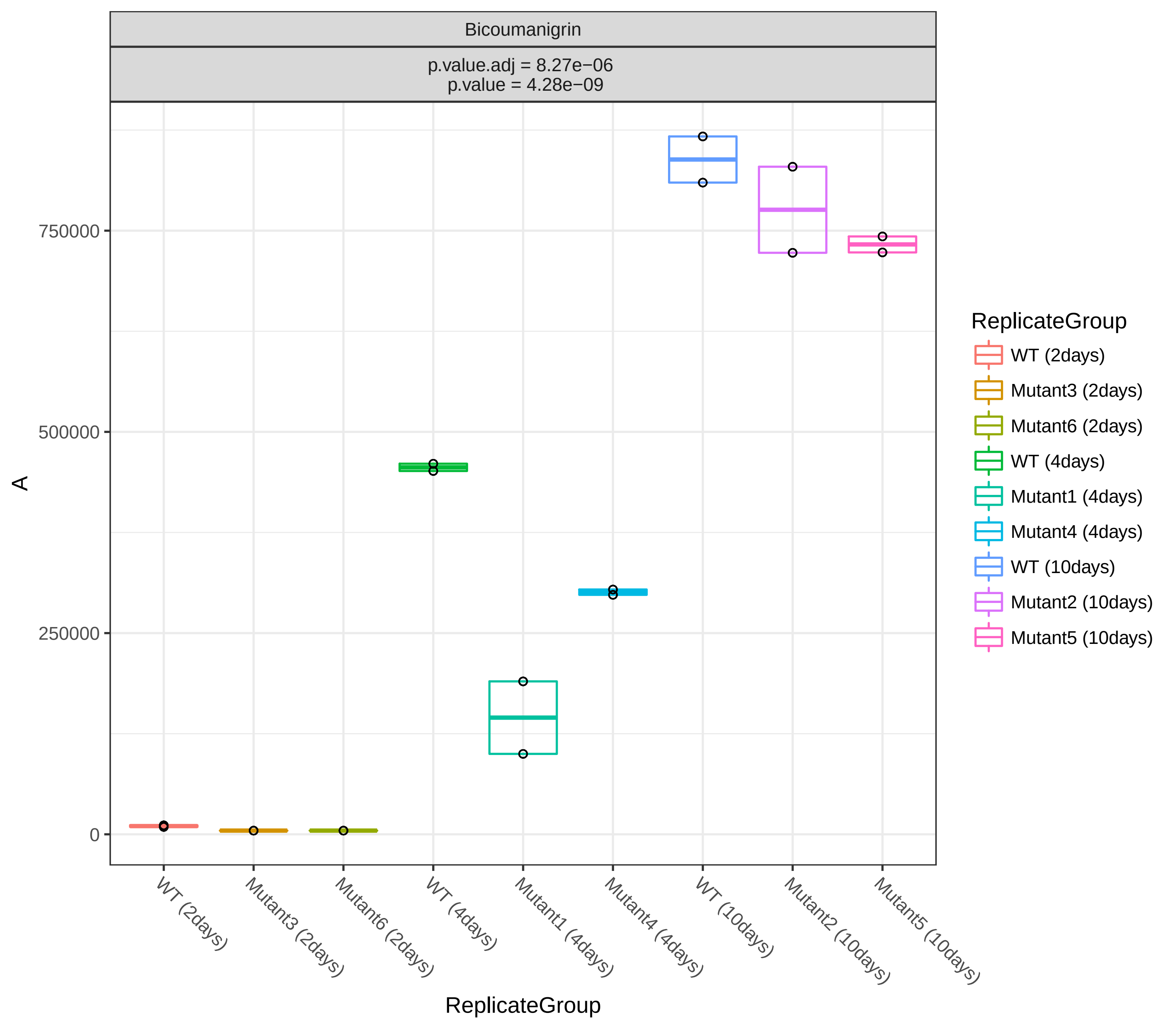

Bicoumanigrin
p.value.adj = 8.27e−06
p.value = 4.28e−09
A
750000
500000
250000
0
ReplicateGroup
WT (2days)
Mutant3 (2days)
Mutant6 (2days)
WT (4days)
Mutant1 (4days)
Mutant4 (4days)
WT (10days)
Mutant2 (10days)
Mutant5 (10days)

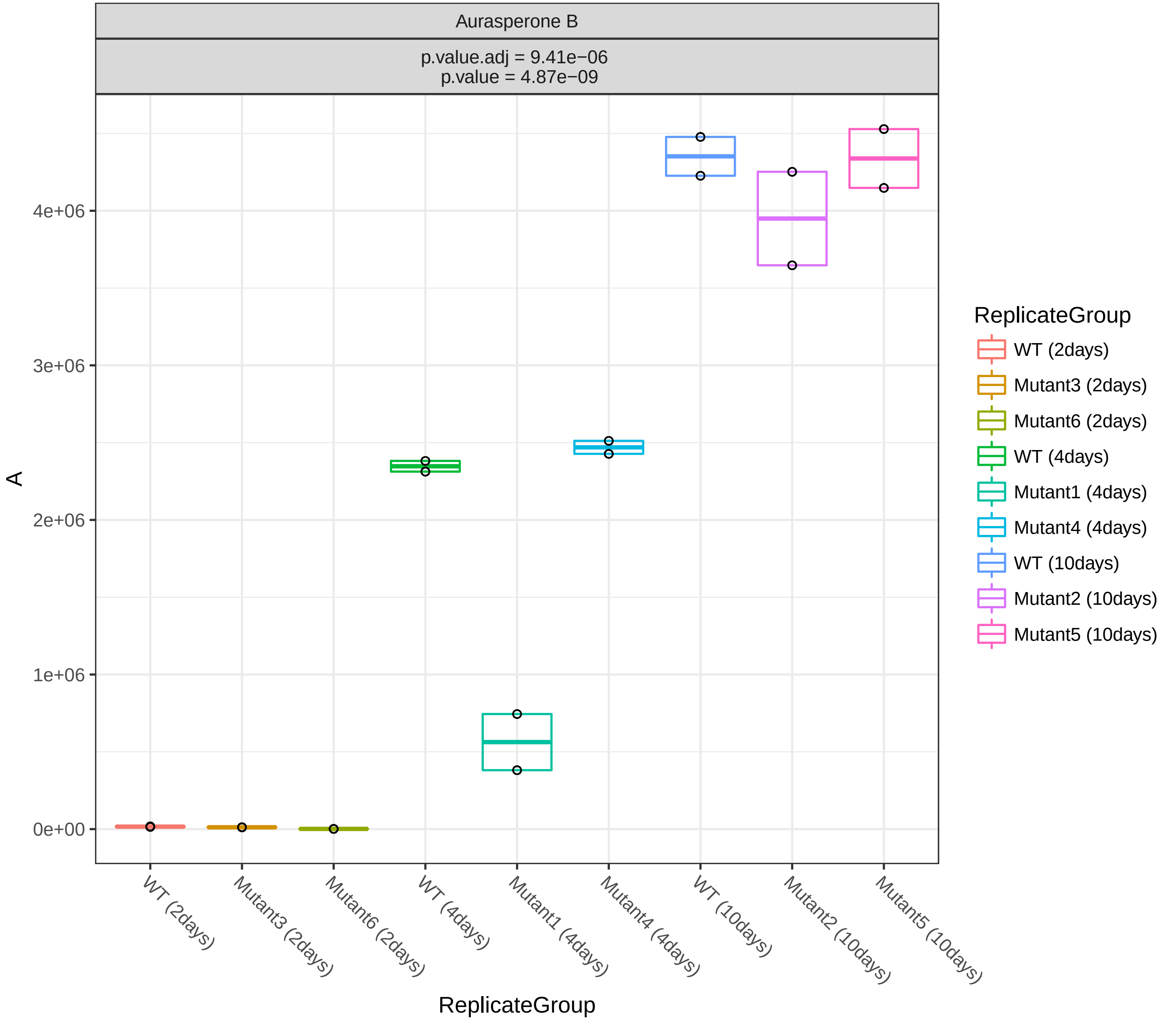
Aurasperone B
p.value.adj = 9.41e−06
p.value = 4.87e−09
A
4e+06
3e+06
2e+06
1e+06
0e+00
ReplicateGroup
WT (2days)
Mutant3 (2days)
Mutant6 (2days)
WT (4days)
Mutant1 (4days)
Mutant4 (4days)
WT (10days)
Mutant2 (10days)
Mutant5 (10days)

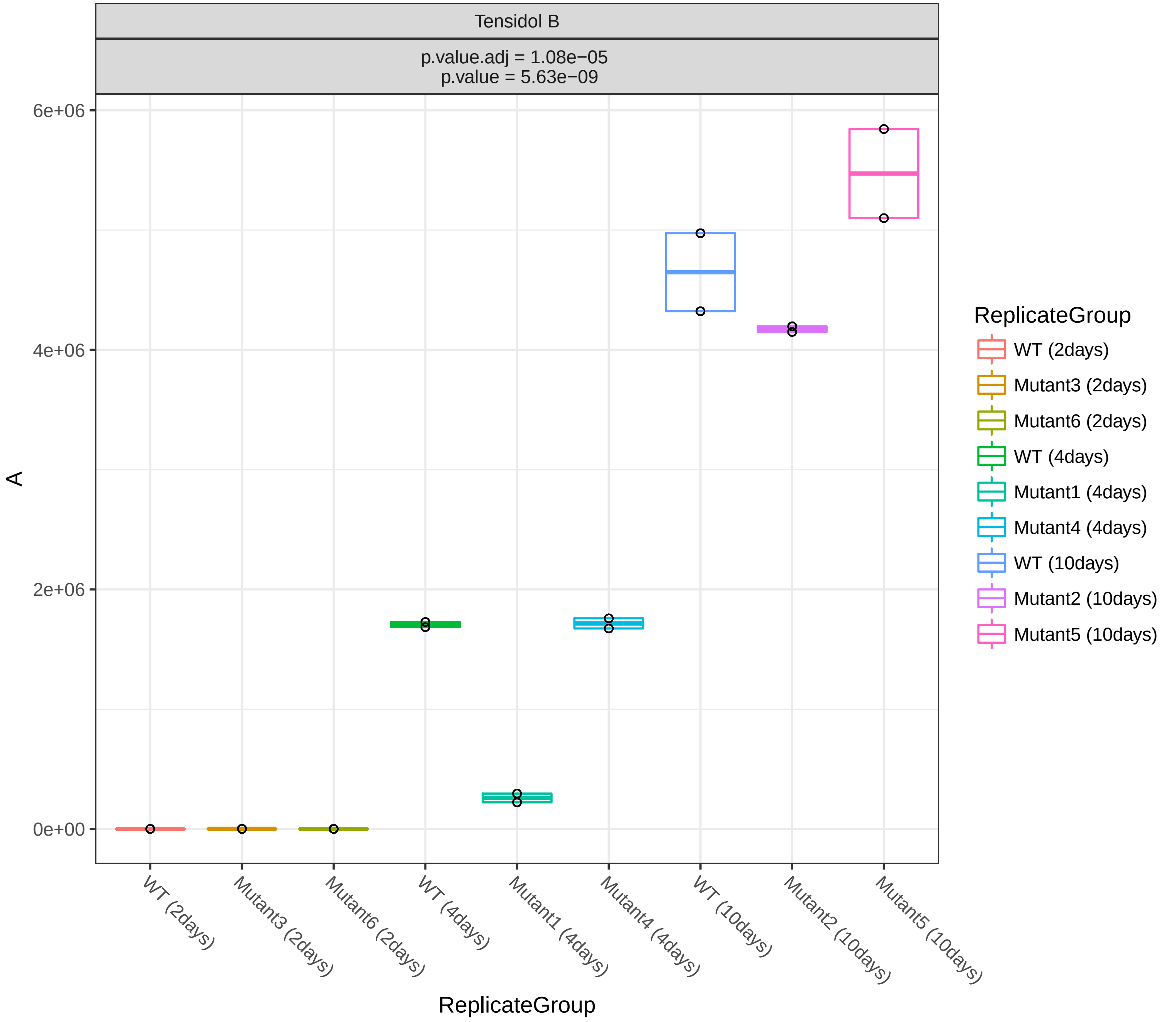
Tensidol B
p.value.adj = 1.08e−05
p.value = 5.63e−09
6e+06
4e+06
2e+06
0e+00
A
WT (2days)
Mutant3 (2days)
Mutant6 (2days)
WT (4days)
Mutant1 (4days)
Mutant4 (4days)
WT (10days)
Mutant2 (10days)
Mutant5 (10days)
ReplicateGroup
ReplicateGroup
WT (2days)
Mutant3 (2days)
Mutant6 (2days)
WT (4days)
Mutant1 (4days)
Mutant4 (4days)
WT (10days)
Mutant2 (10days)
Mutant5 (10days)

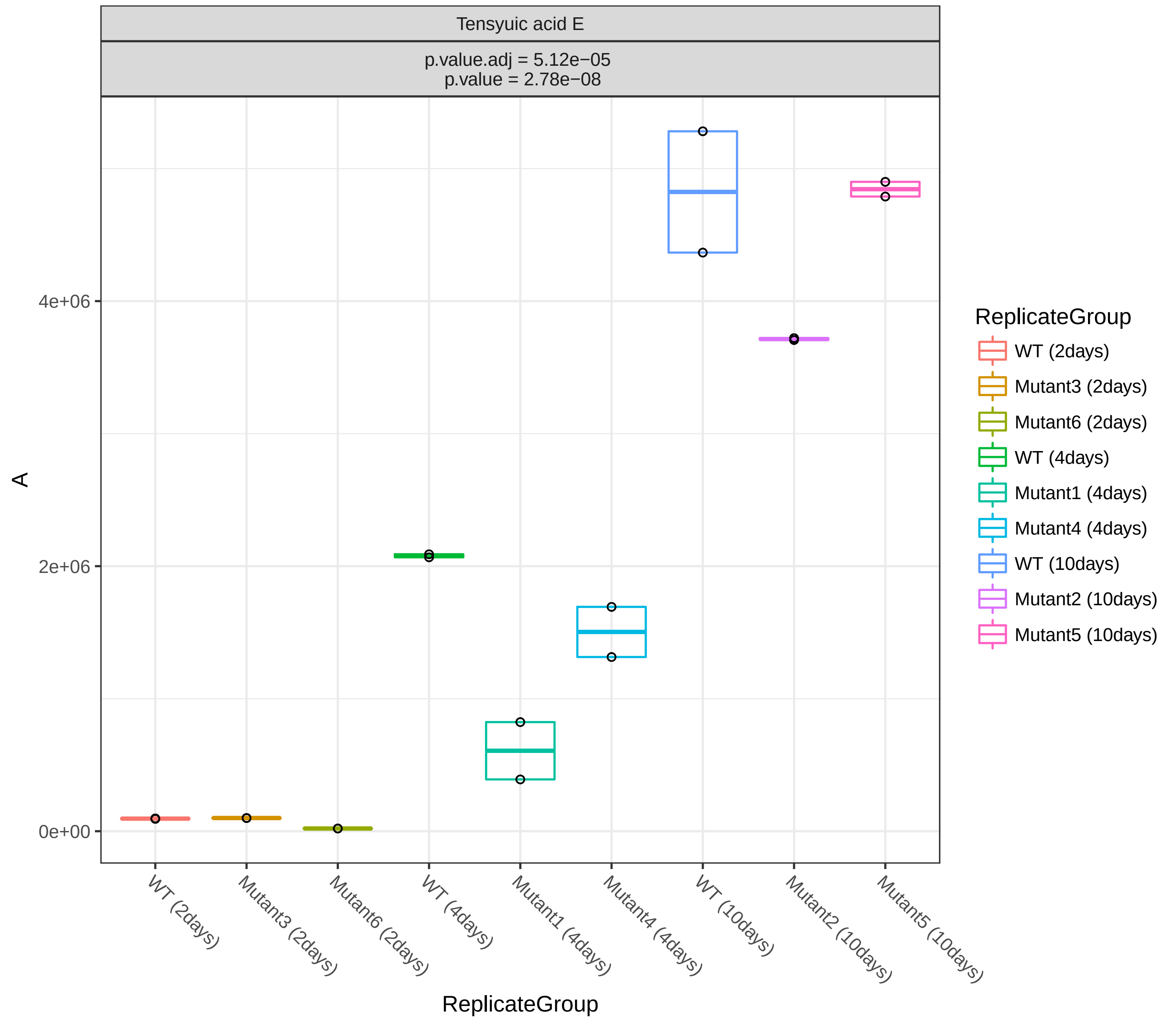
Tensyuic acid E
p.value.adj = 5.12e−05
p.value = 2.78e−08
A
4e+06
2e+06
0e+00
ReplicateGroup
WT (2days)
Mutant3 (2days)
Mutant6 (2days)
WT (4days)
Mutant1 (4days)
Mutant4 (4days)
WT (10days)
Mutant2 (10days)
Mutant5 (10days)

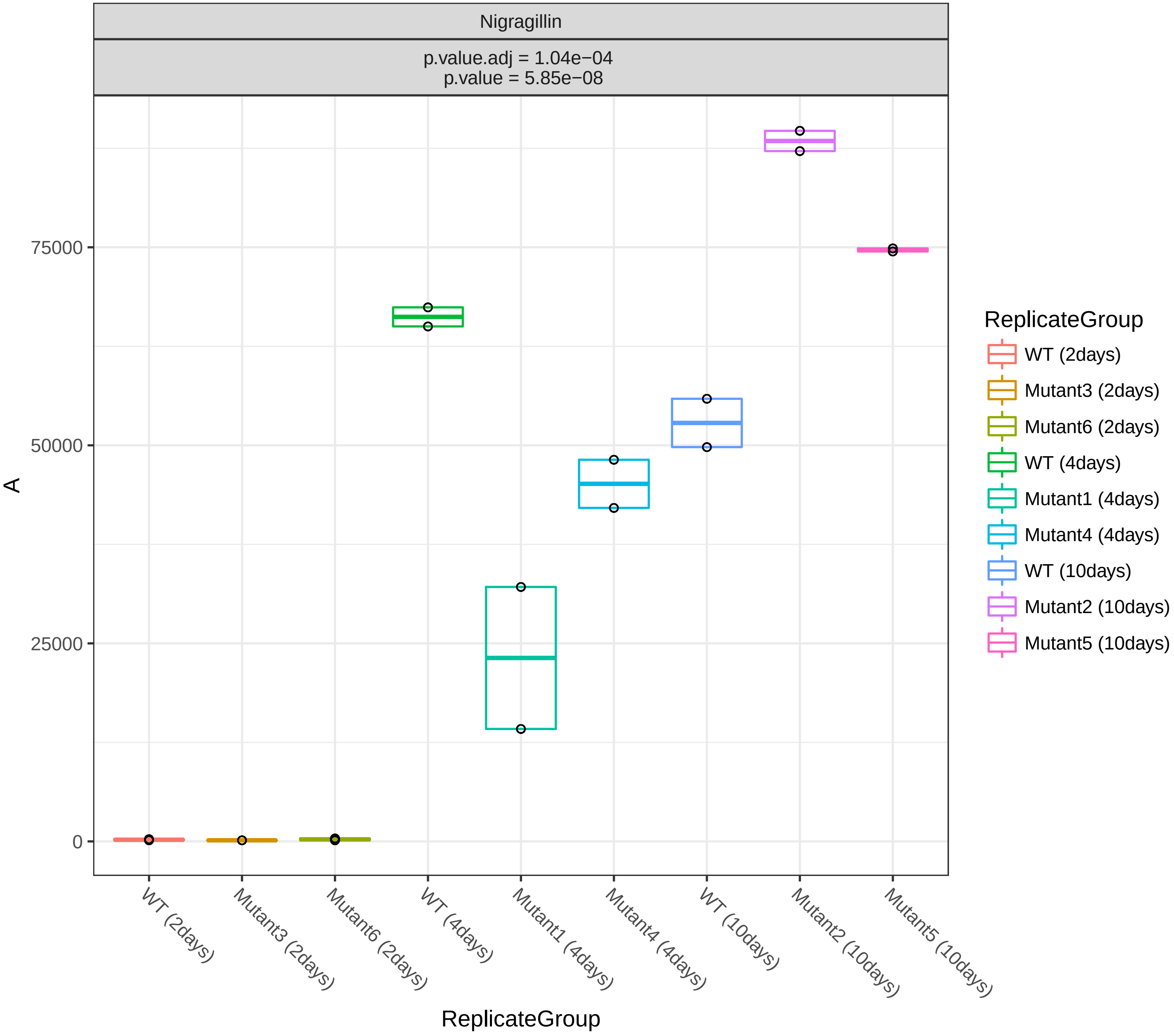
Nigragillin
p.value.adj = 1.04e−04
p.value = 5.85e−08
75000
50000
25000
0
A
WT (2days)
Mutant3 (2days)
Mutant6 (2days)
WT (4days)
Mutant1 (4days)
Mutant4 (4days)
WT (10days)
Mutant2 (10days)
Mutant5 (10days)
ReplicateGroup
ReplicateGroup
WT (2days)
Mutant3 (2days)
Mutant6 (2days)
WT (4days)
Mutant1 (4days)
Mutant4 (4days)
WT (10days)
Mutant2 (10days)
Mutant5 (10days)

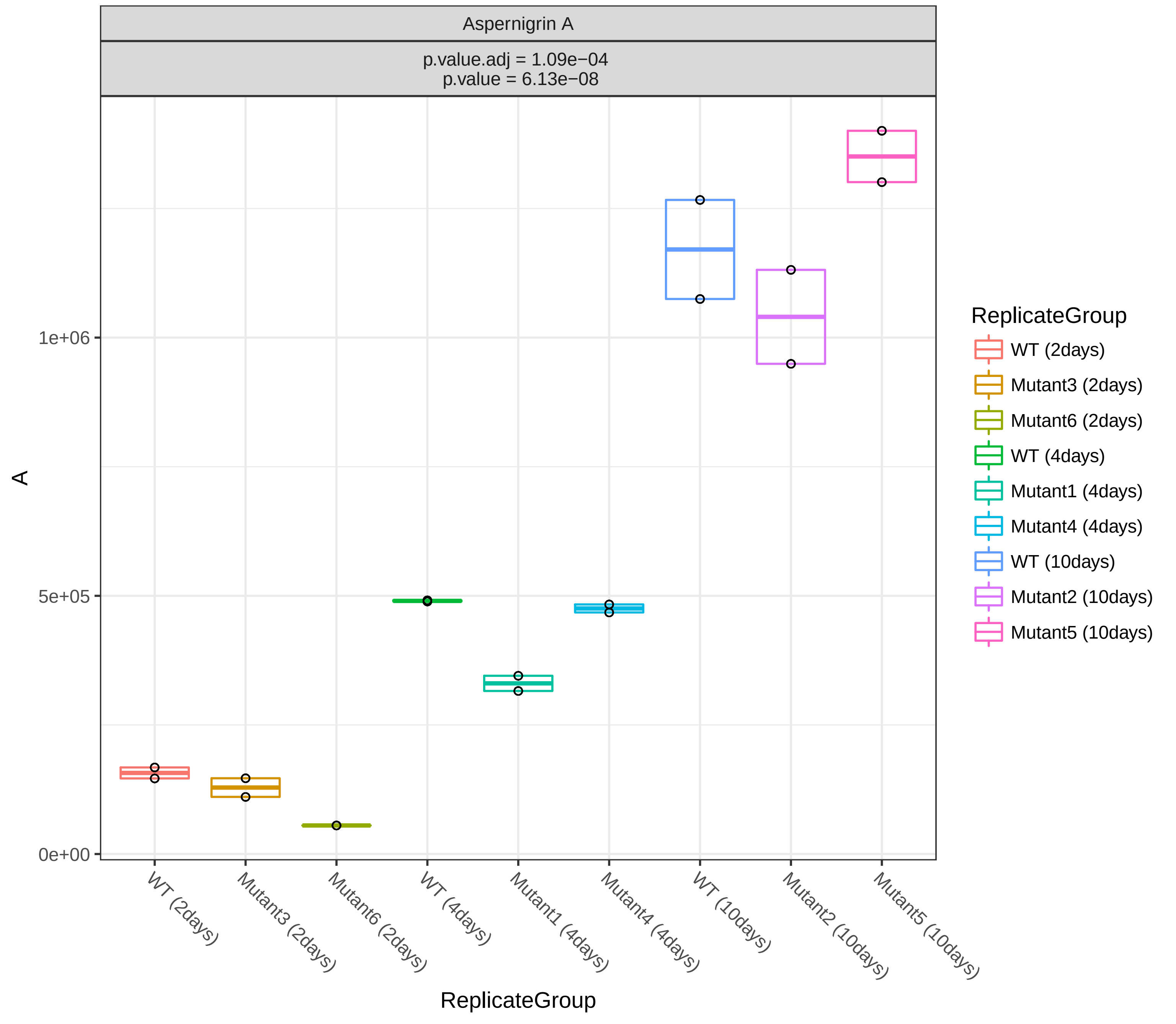

Aspernigrin A
p.value.adj = 1.09e−04
p.value = 6.13e−08
A
1e+06
5e+05
0e+00
WT (2days)
Mutant3 (2days)
Mutant6 (2days)
WT (4days)
Mutant1 (4days)
Mutant4 (4days)
WT (10days)
Mutant2 (10days)
Mutant5 (10days)
ReplicateGroup
ReplicateGroup
WT (2days)
Mutant3 (2days)
Mutant6 (2days)
WT (4days)
Mutant1 (4days)
Mutant4 (4days)
WT (10days)
Mutant2 (10days)
Mutant5 (10days)

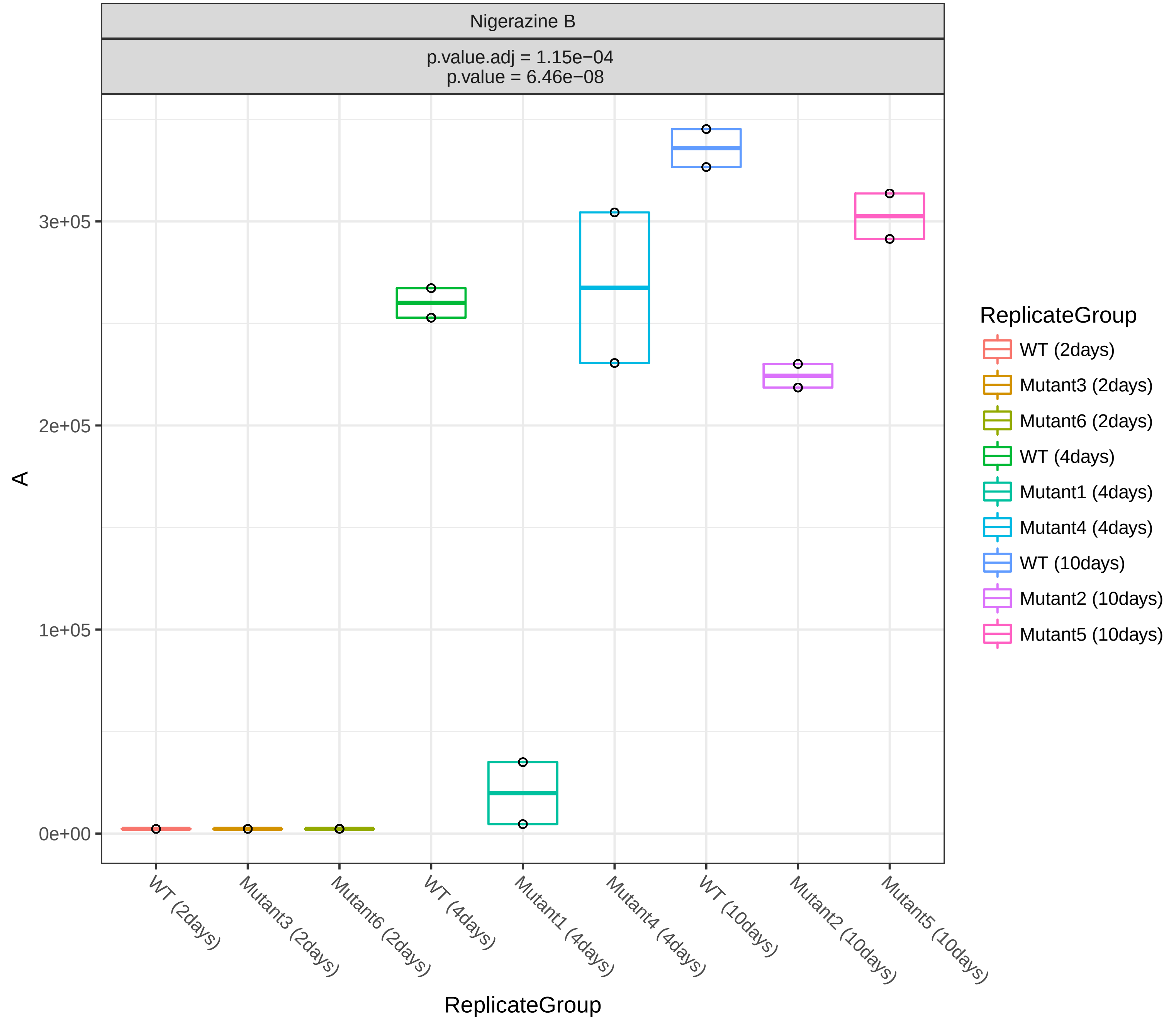
Nigerazine B
p.value.adj = 1.15e−04
p.value = 6.46e−08
A
3e+05
2e+05
1e+05
0e+00
ReplicateGroup
WT (2days)
Mutant3 (2days)
Mutant6 (2days)
WT (4days)
Mutant1 (4days)
Mutant4 (4days)
WT (10days)
Mutant2 (10days)
Mutant5 (10days)
ReplicateGroup

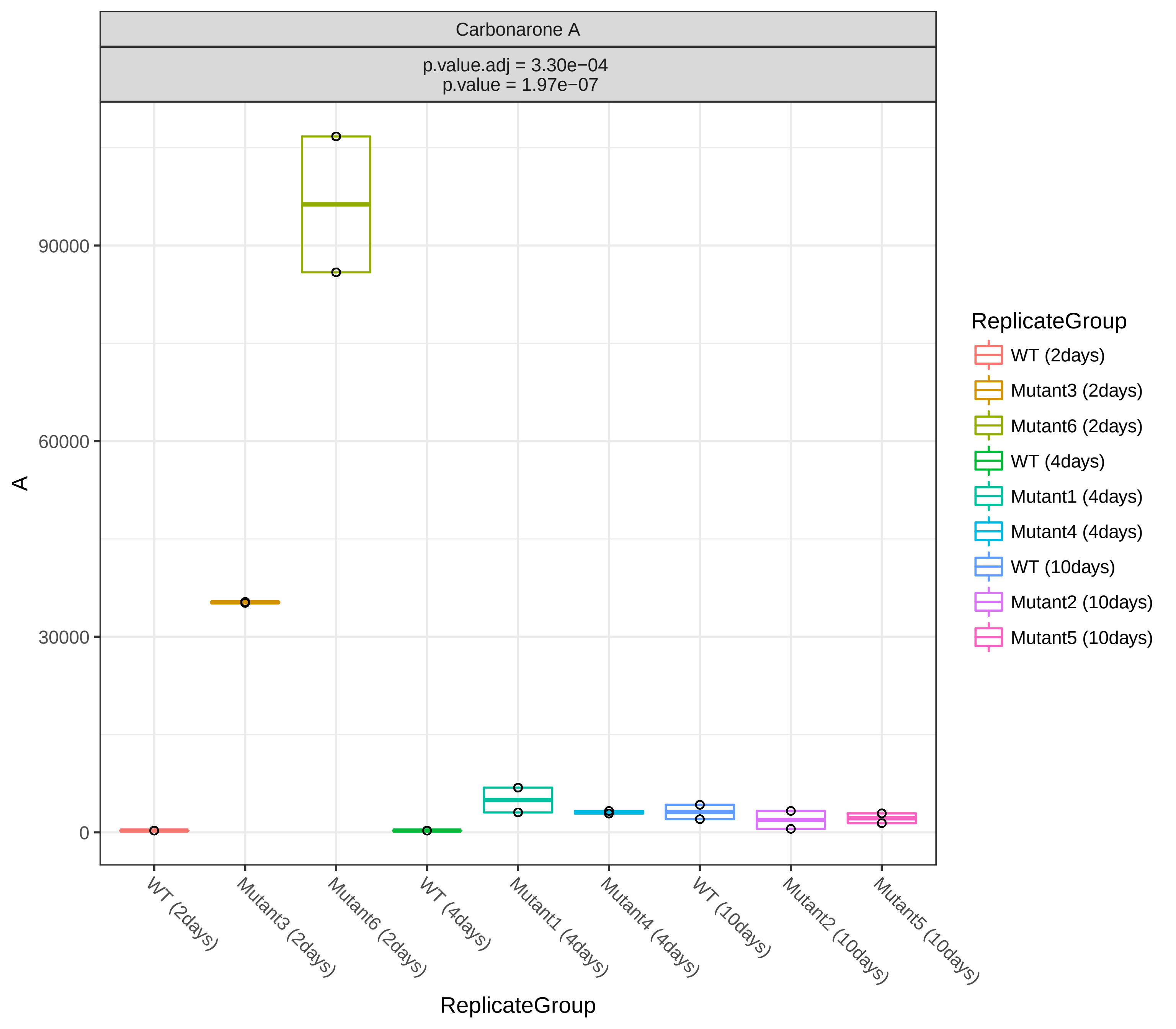

Carbonarone A
p.value.adj = 3.30e−04
p.value = 1.97e−07
A
90000
60000
30000
0
ReplicateGroup
WT (2days)
Mutant3 (2days)
Mutant6 (2days)
WT (4days)
Mutant1 (4days)
Mutant4 (4days)
WT (10days)
Mutant2 (10days)
Mutant5 (10days)

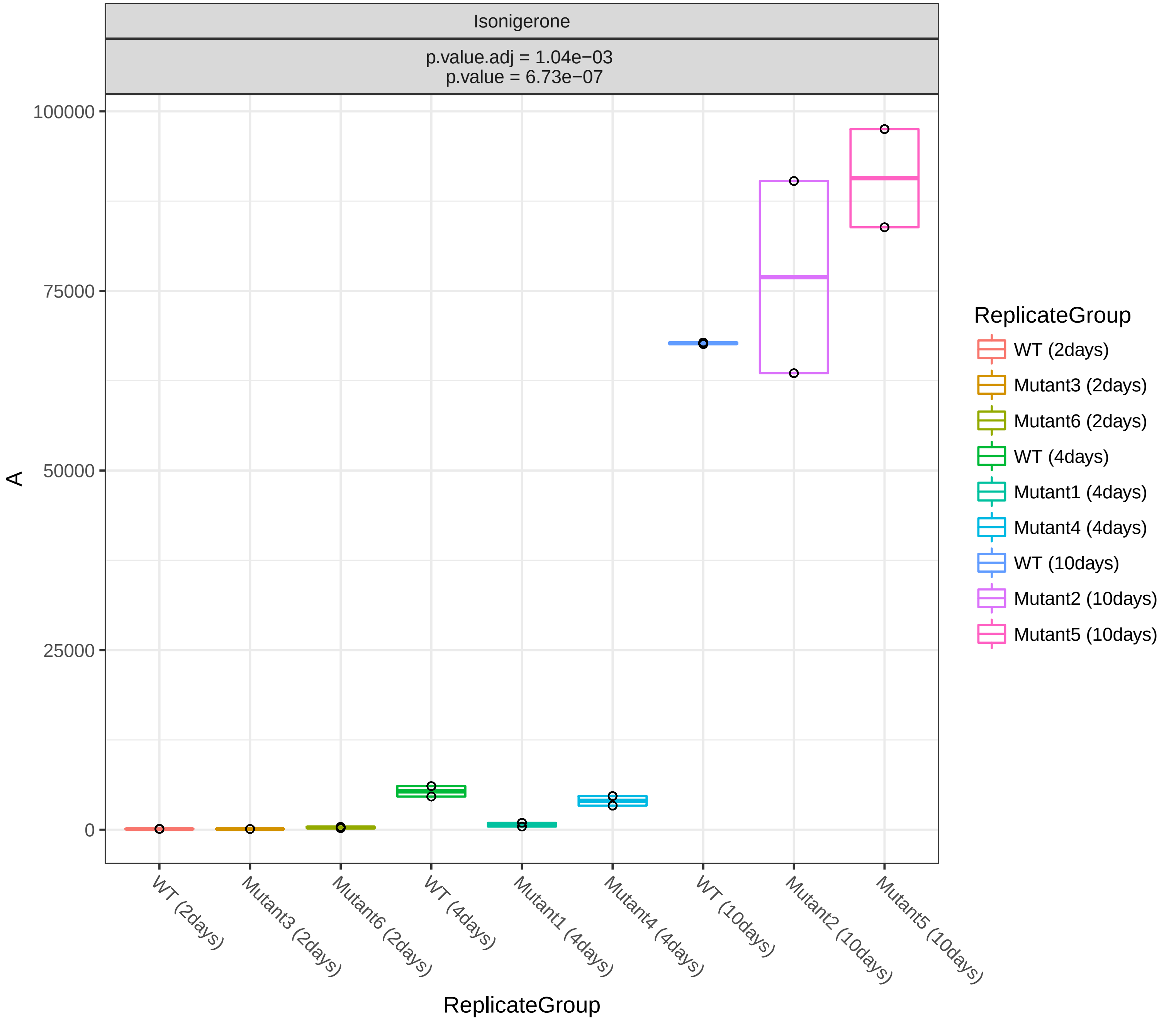
Isonigerone
p.value.adj = 1.04e−03
p.value = 6.73e−07
100000
75000
50000
25000
0
A
WT (2days)
Mutant3 (2days)
Mutant6 (2days)
WT (4days)
Mutant1 (4days)
Mutant4 (4days)
WT (10days)
Mutant2 (10days)
Mutant5 (10days)
ReplicateGroup
ReplicateGroup
WT (2days)
Mutant3 (2days)
Mutant6 (2days)
WT (4days)
Mutant1 (4days)
Mutant4 (4days)
WT (10days)
Mutant2 (10days)
Mutant5 (10days)

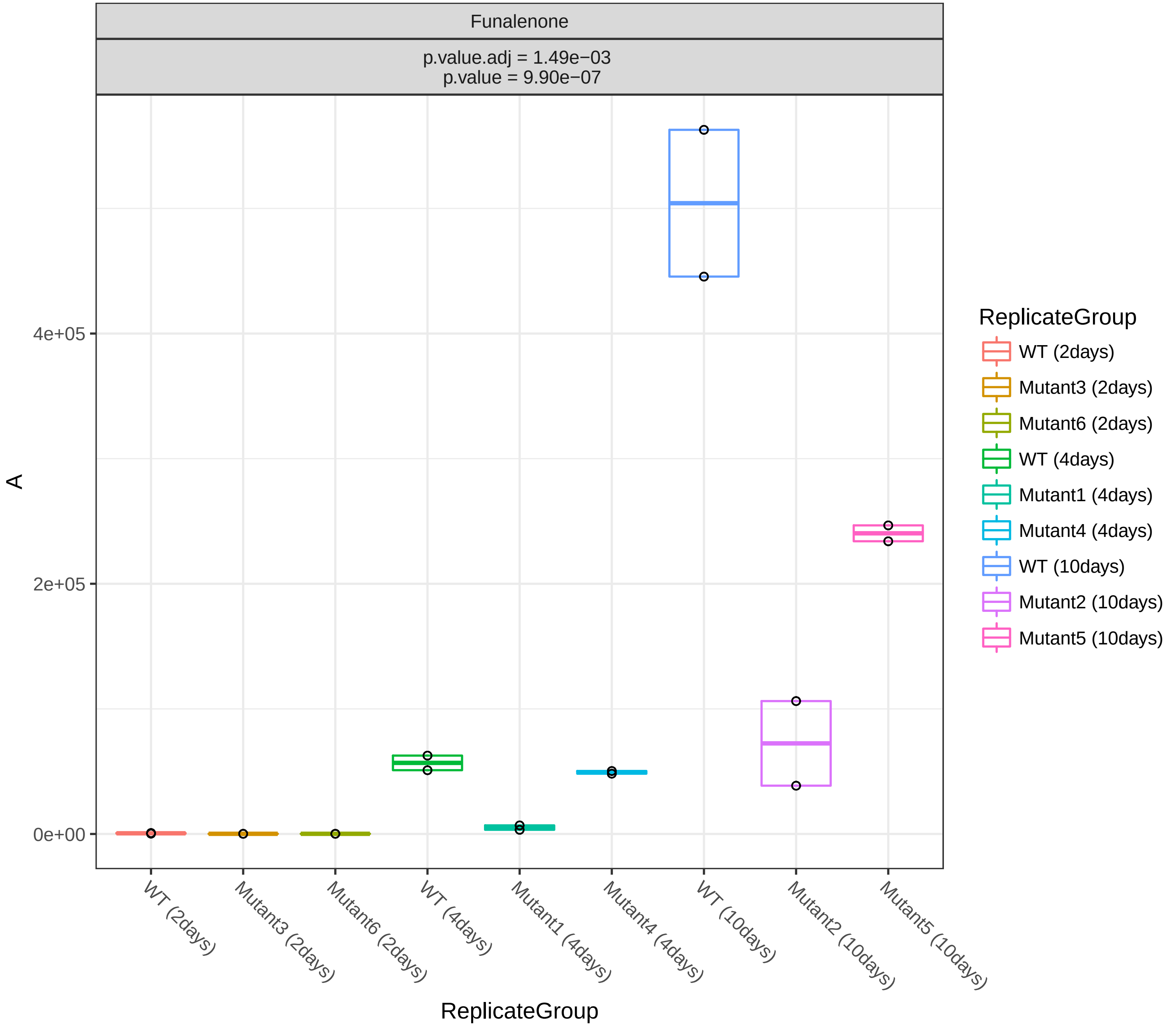

Funalenone
p.value.adj = 1.49e−03
p.value = 9.90e−07
A
0e+00
2e+05
4e+05
ReplicateGroup
WT (2days)
Mutant3 (2days)
Mutant6 (2days)
WT (4days)
Mutant1 (4days)
Mutant4 (4days)
WT (10days)
Mutant2 (10days)
Mutant5 (10days)
ReplicateGroup
WT (2days)
Mutant3 (2days)
Mutant6 (2days)
WT (4days)
Mutant1 (4days)
Mutant4 (4days)
WT (10days)
Mutant2 (10days)
Mutant5 (10days)

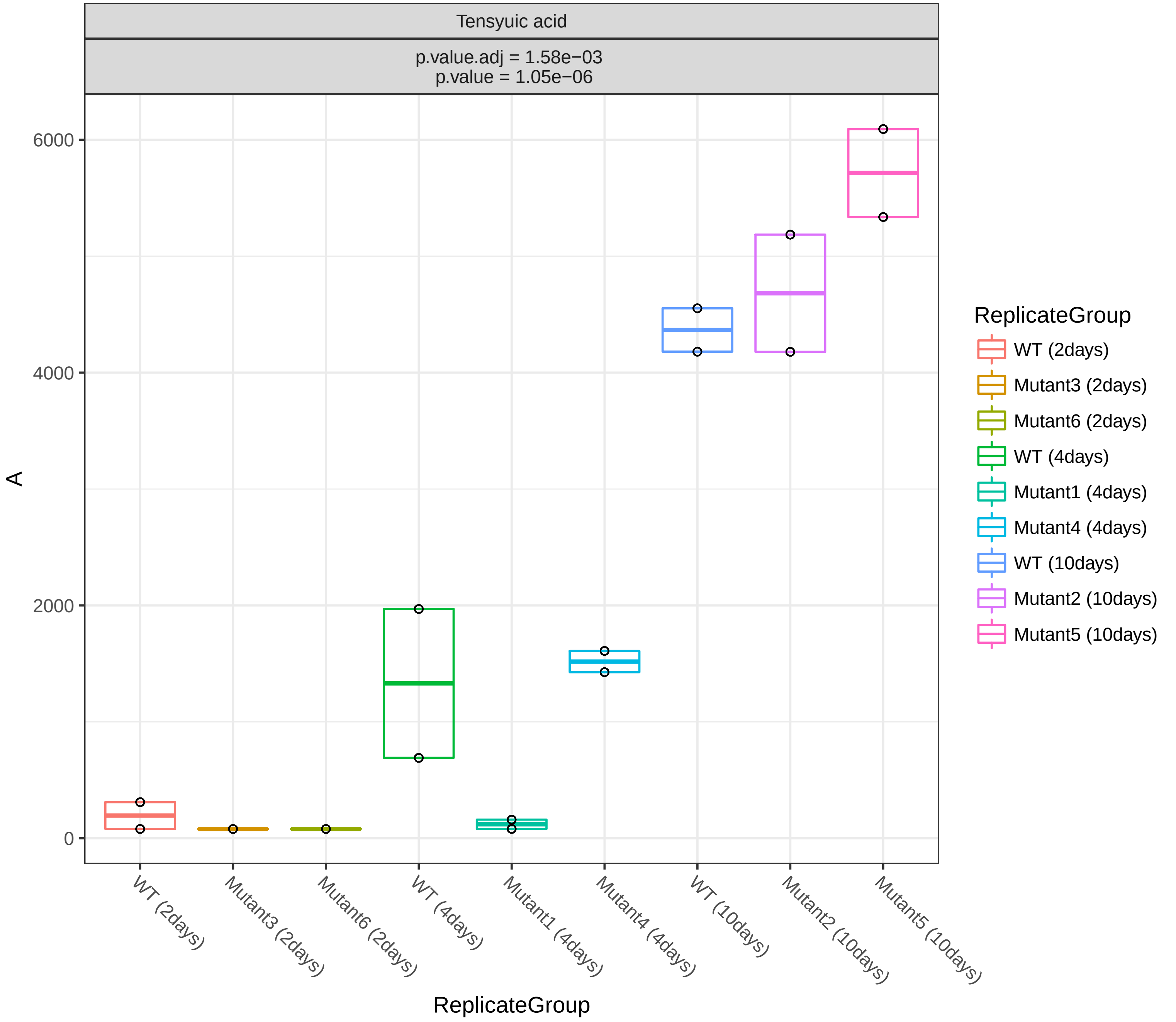
Tensyuic acid
p.value.adj = 1.58e−03
p.value = 1.05e−06
6000
4000
2000
0
A
WT (2days)
Mutant3 (2days)
Mutant6 (2days)
WT (4days)
Mutant1 (4days)
Mutant4 (4days)
WT (10days)
Mutant2 (10days)
Mutant5 (10days)
ReplicateGroup
ReplicateGroup
WT (2days)
Mutant3 (2days)
Mutant6 (2days)
WT (4days)
Mutant1 (4days)
Mutant4 (4days)
WT (10days)
Mutant2 (10days)
Mutant5 (10days)

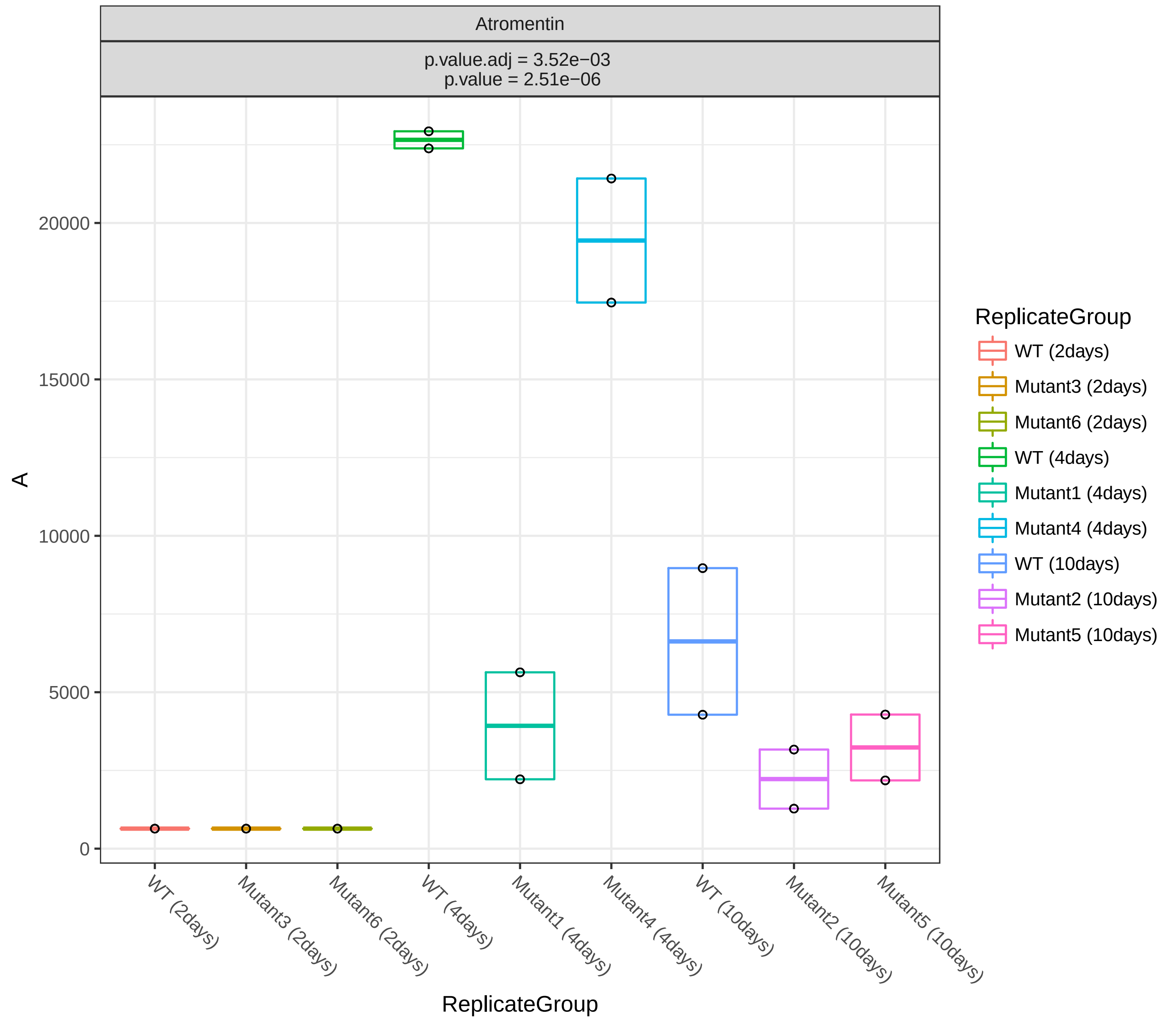
Atromentin
p.value.adj = 3.52e−03
p.value = 2.51e−06
A
20000
15000
10000
5000
0
ReplicateGroup
WT (2days)
Mutant3 (2days)
Mutant6 (2days)
WT (4days)
Mutant1 (4days)
Mutant4 (4days)
WT (10days)
Mutant2 (10days)
Mutant5 (10days)
ReplicateGroup
WT (2days)
Mutant3 (2days)
Mutant6 (2days)
WT (4days)
Mutant1 (4days)
Mutant4 (4days)
WT (10days)
Mutant2 (10days)
Mutant5 (10days)

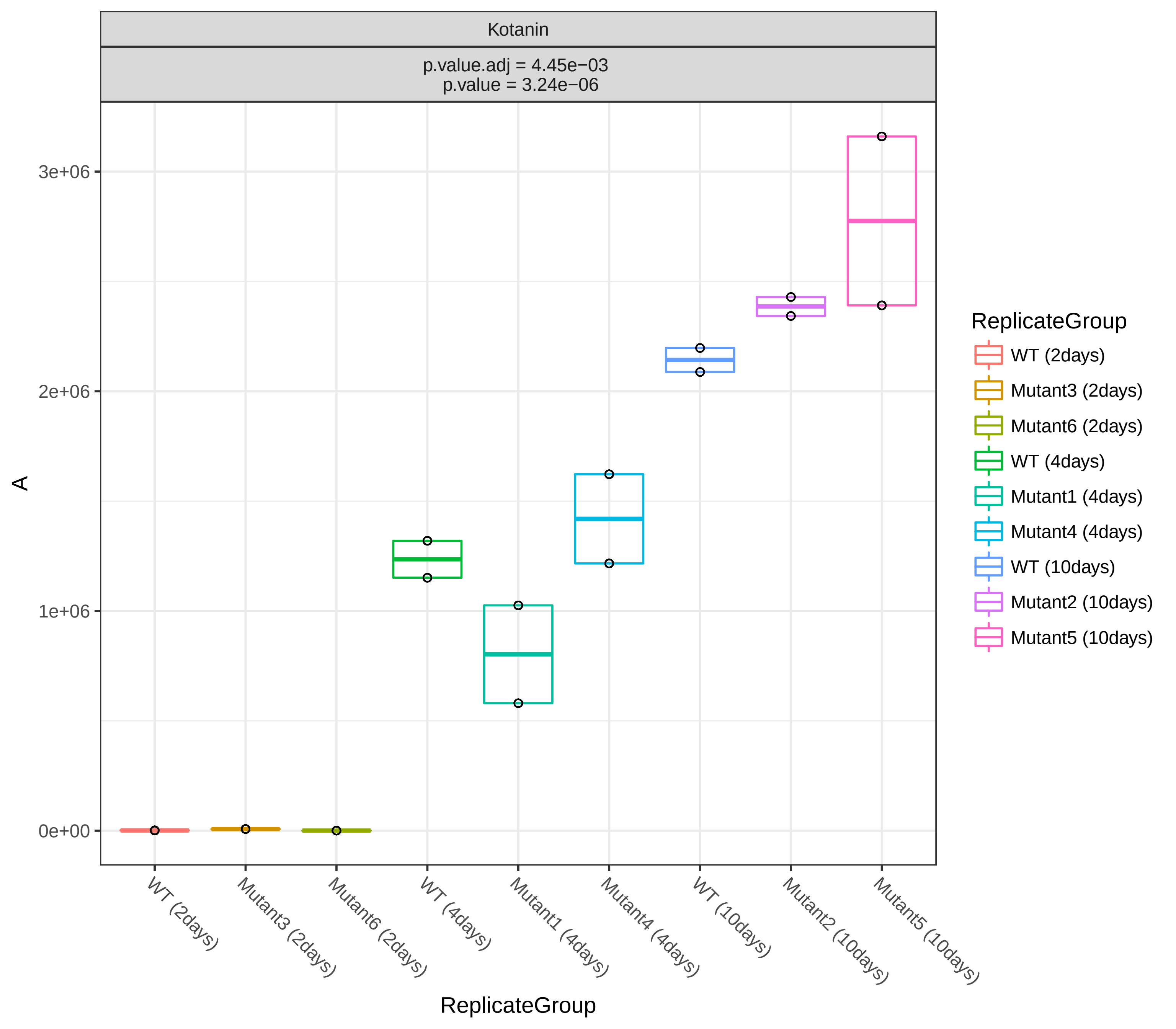
Kotanin
p.value.adj = 4.45e−03
p.value = 3.24e−06
3e+06
2e+06
1e+06
0e+00
A
WT (2days)
Mutant3 (2days)
Mutant6 (2days)
WT (4days)
Mutant1 (4days)
Mutant4 (4days)
WT (10days)
Mutant2 (10days)
Mutant5 (10days)
ReplicateGroup
ReplicateGroup
WT (2days)
Mutant3 (2days)
Mutant6 (2days)
WT (4days)
Mutant1 (4days)
Mutant4 (4days)
WT (10days)
Mutant2 (10days)
Mutant5 (10days)

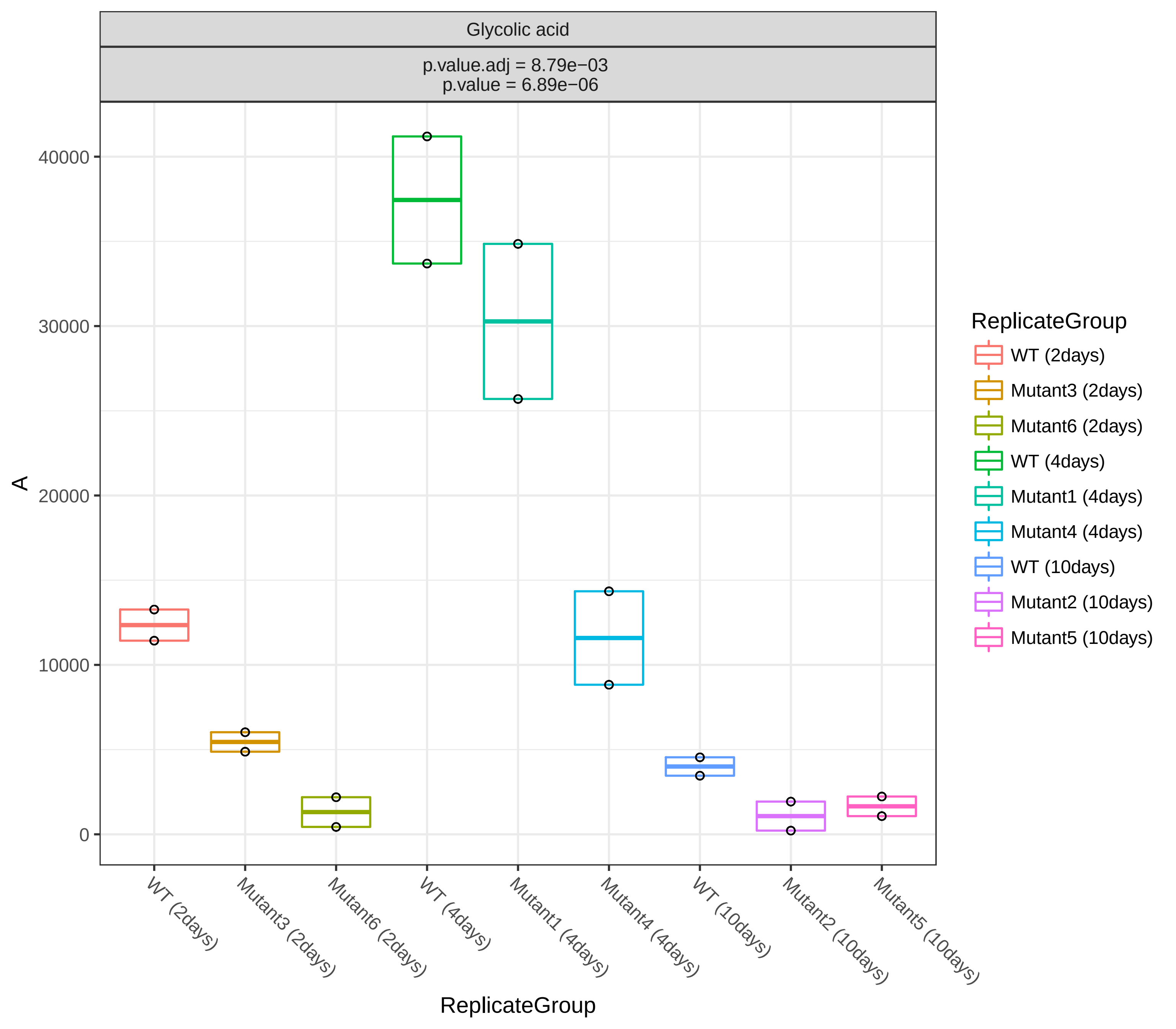
Glycolic acid
p.value.adj = 8.79e−03
p.value = 6.89e−06
A
40000
30000
20000
10000
0
WT (2days)
Mutant3 (2days)
Mutant6 (2days)
WT (4days)
Mutant1 (4days)
Mutant4 (4days)
WT (10days)
Mutant2 (10days)
Mutant5 (10days)
ReplicateGroup
ReplicateGroup
WT (2days)
Mutant3 (2days)
Mutant6 (2days)
WT (4days)
Mutant1 (4days)
Mutant4 (4days)
WT (10days)
Mutant2 (10days)
Mutant5 (10days)

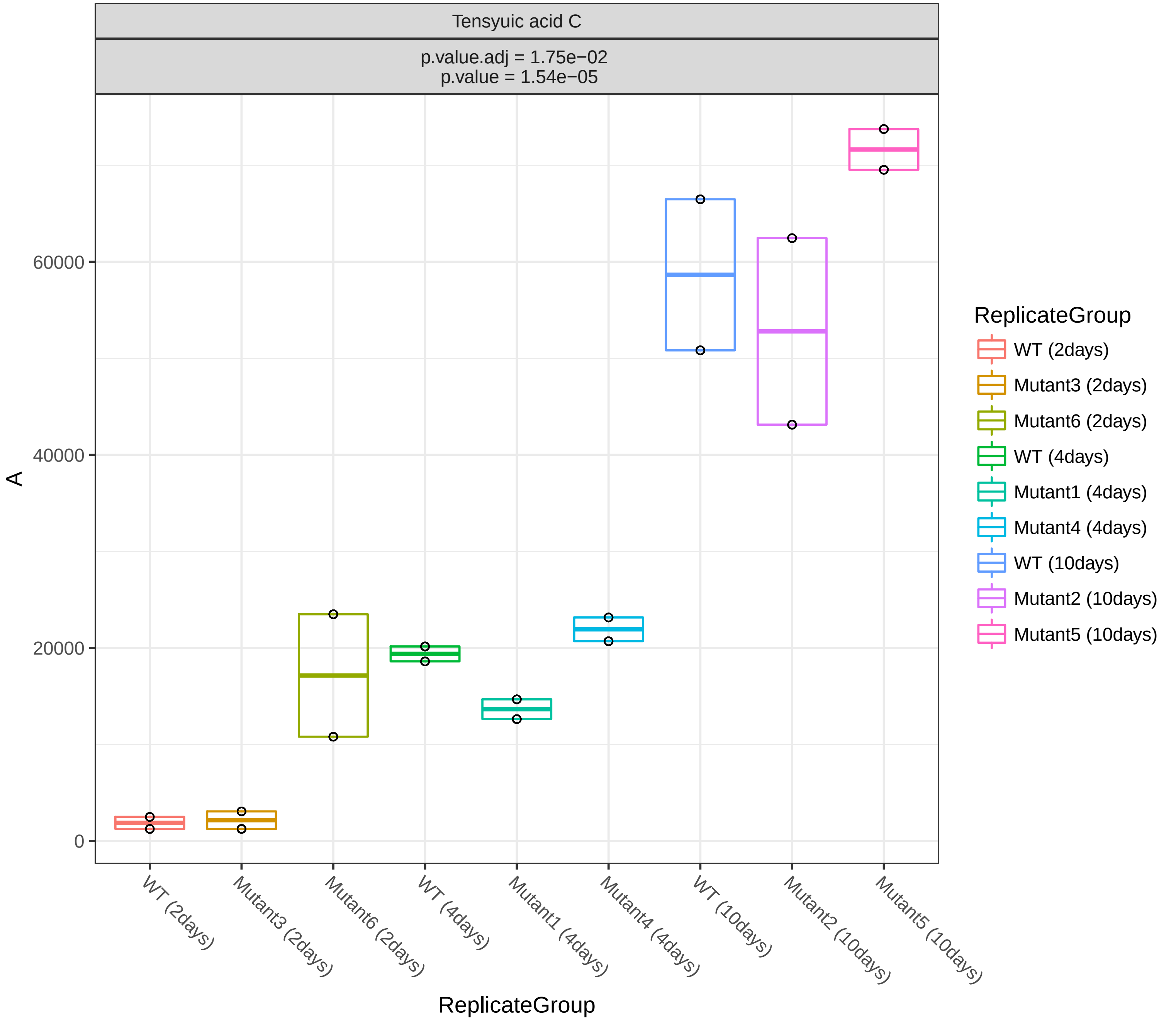
Tensyuic acid C
p.value.adj = 1.75e−02
p.value = 1.54e−05
A
60000
40000
20000
0
ReplicateGroup
WT (2days)
Mutant3 (2days)
Mutant6 (2days)
WT (4days)
Mutant1 (4days)
Mutant4 (4days)
WT (10days)
Mutant2 (10days)
Mutant5 (10days)

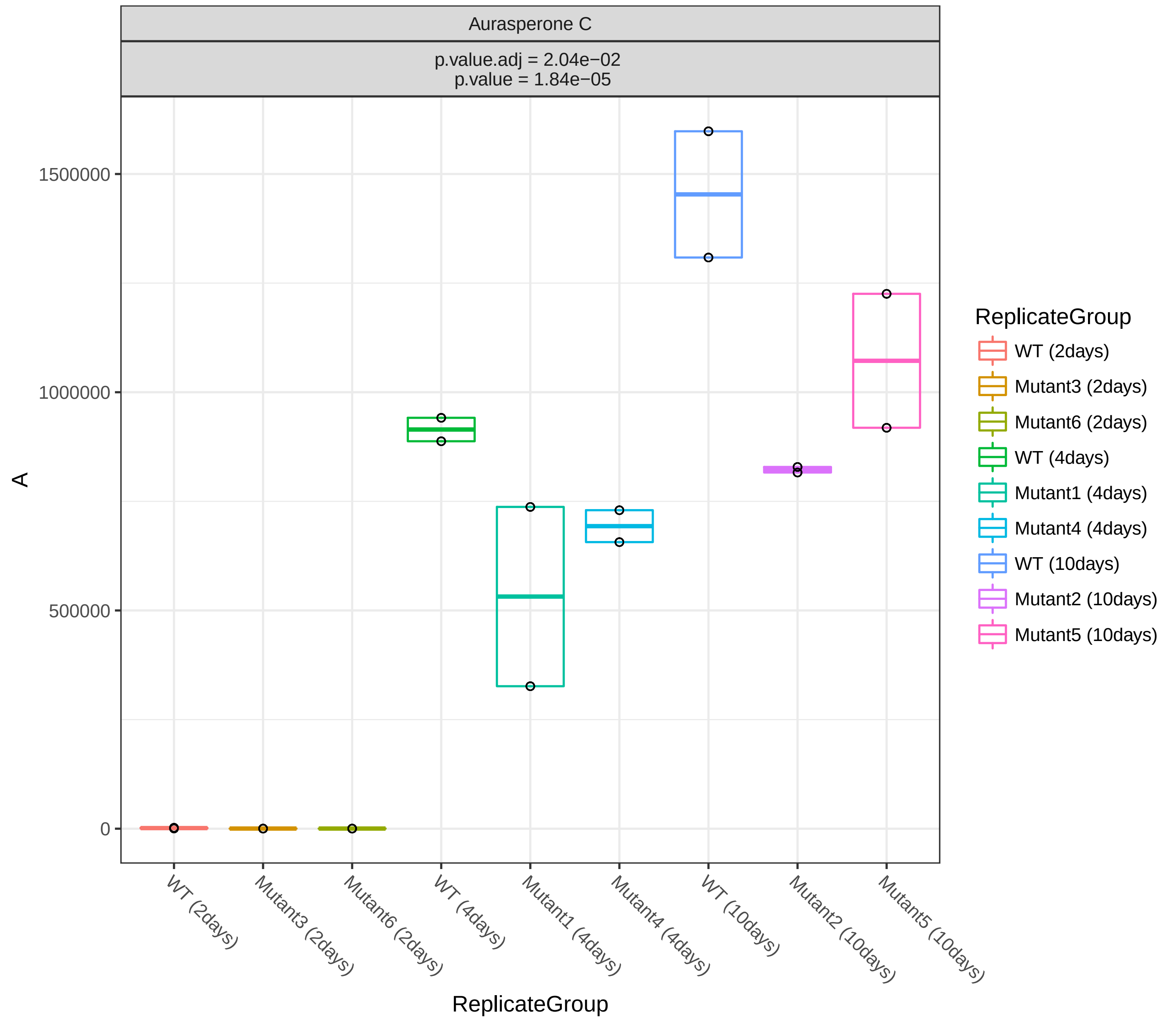

Aurasperone C
p.value.adj = 2.04e−02
p.value = 1.84e−05
1500000
1000000
500000
0
A
WT (2days)
Mutant3 (2days)
Mutant6 (2days)
WT (4days)
Mutant1 (4days)
Mutant4 (4days)
WT (10days)
Mutant2 (10days)
Mutant5 (10days)
ReplicateGroup
ReplicateGroup
WT (2days)
Mutant3 (2days)
Mutant6 (2days)
WT (4days)
Mutant1 (4days)
Mutant4 (4days)
WT (10days)
Mutant2 (10days)
Mutant5 (10days)

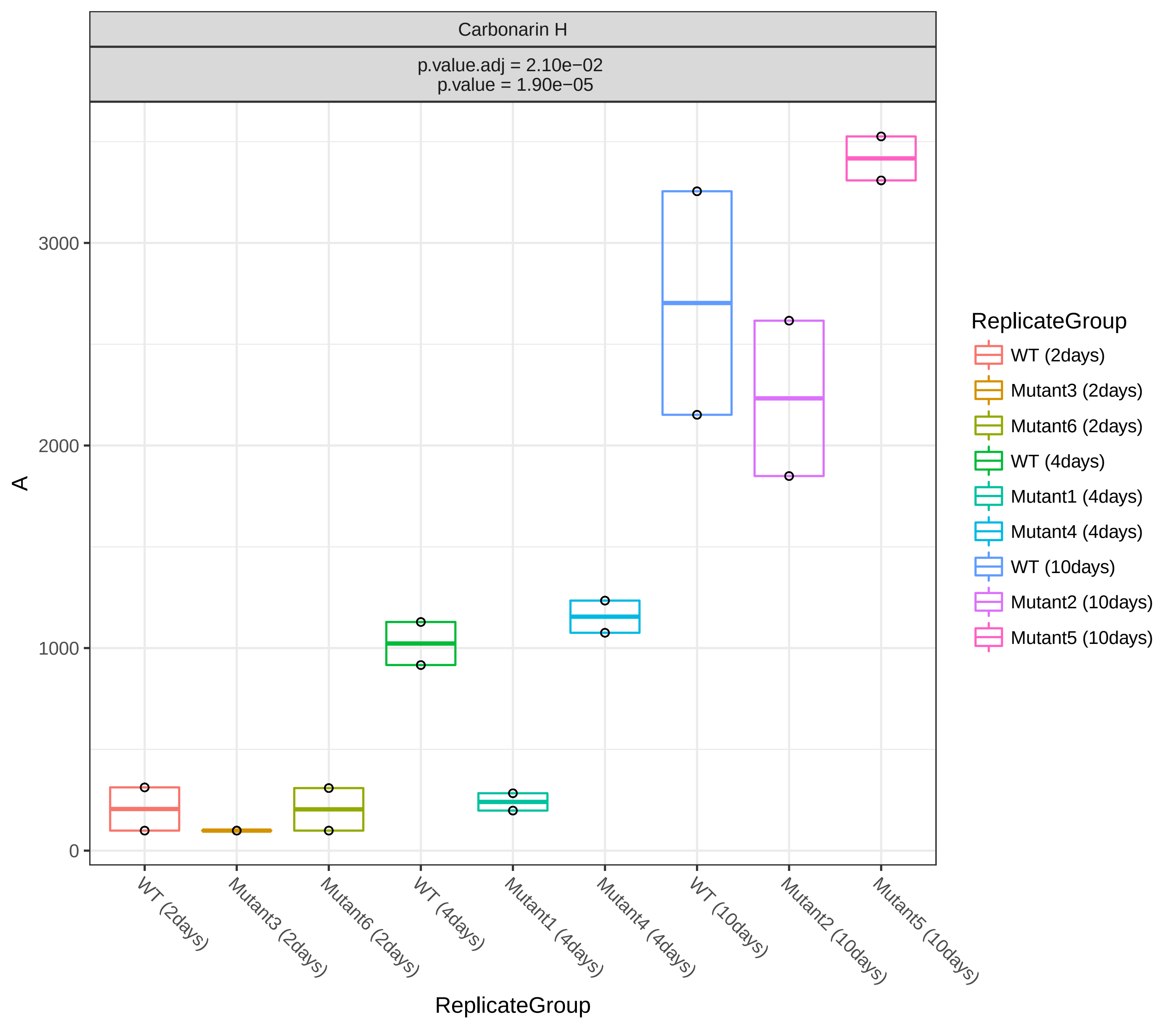
Carbonarin H
p.value.adj = 2.10e−02
p.value = 1.90e−05
A
0
1000
2000
3000
ReplicateGroup
WT (2days)
Mutant3 (2days)
Mutant6 (2days)
WT (4days)
Mutant1 (4days)
Mutant4 (4days)
WT (10days)
Mutant2 (10days)
Mutant5 (10days)

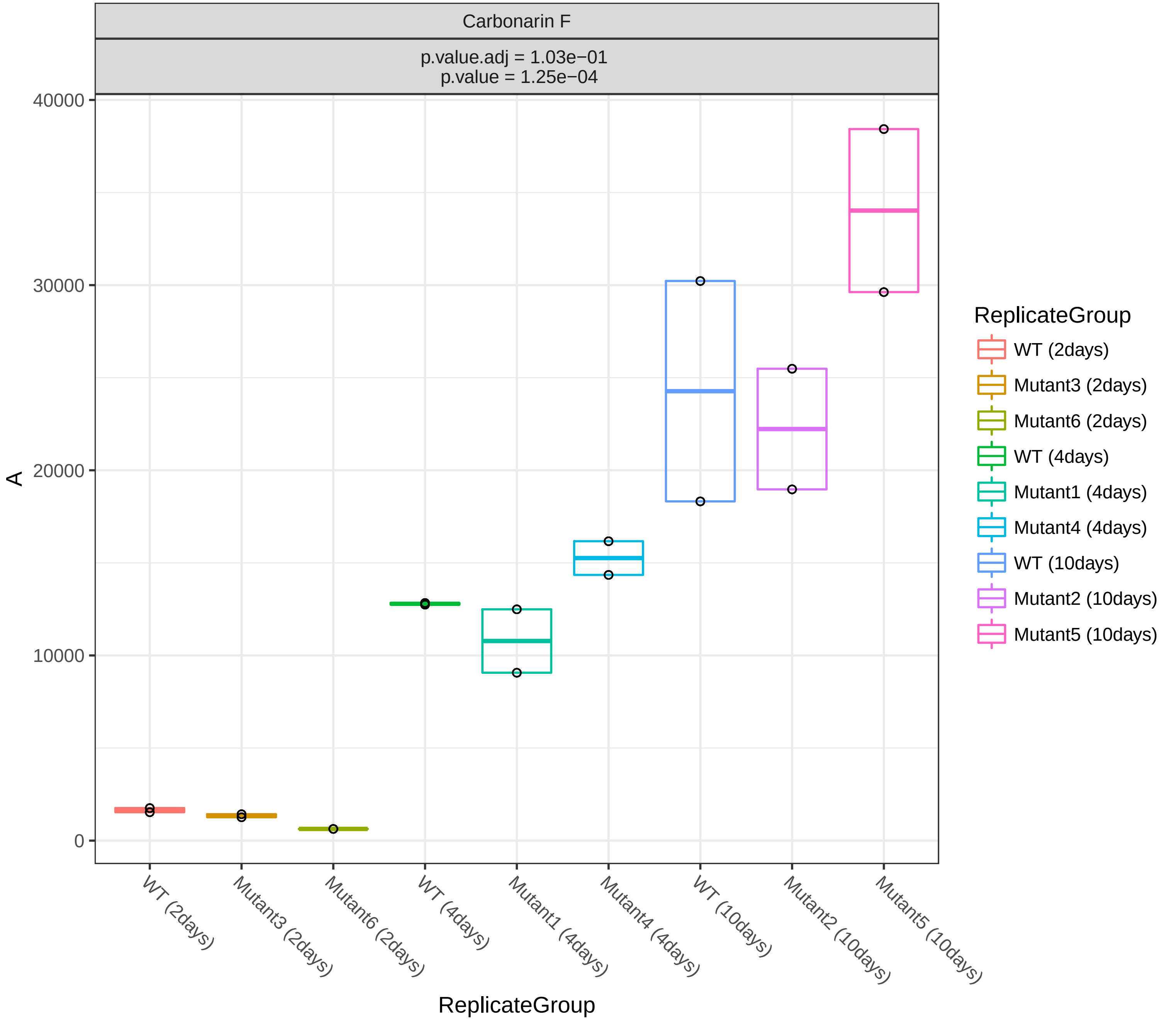

Carbonarin F
p.value.adj = 1.03e−01
p.value = 1.25e−04
A
40000
30000
20000
10000
0
WT (2days)
Mutant3 (2days)
Mutant6 (2days)
WT (4days)
Mutant1 (4days)
Mutant4 (4days)
WT (10days)
Mutant2 (10days)
Mutant5 (10days)
ReplicateGroup
ReplicateGroup
WT (2days)
Mutant3 (2days)
Mutant6 (2days)
WT (4days)
Mutant1 (4days)
Mutant4 (4days)
WT (10days)
Mutant2 (10days)
Mutant5 (10days)

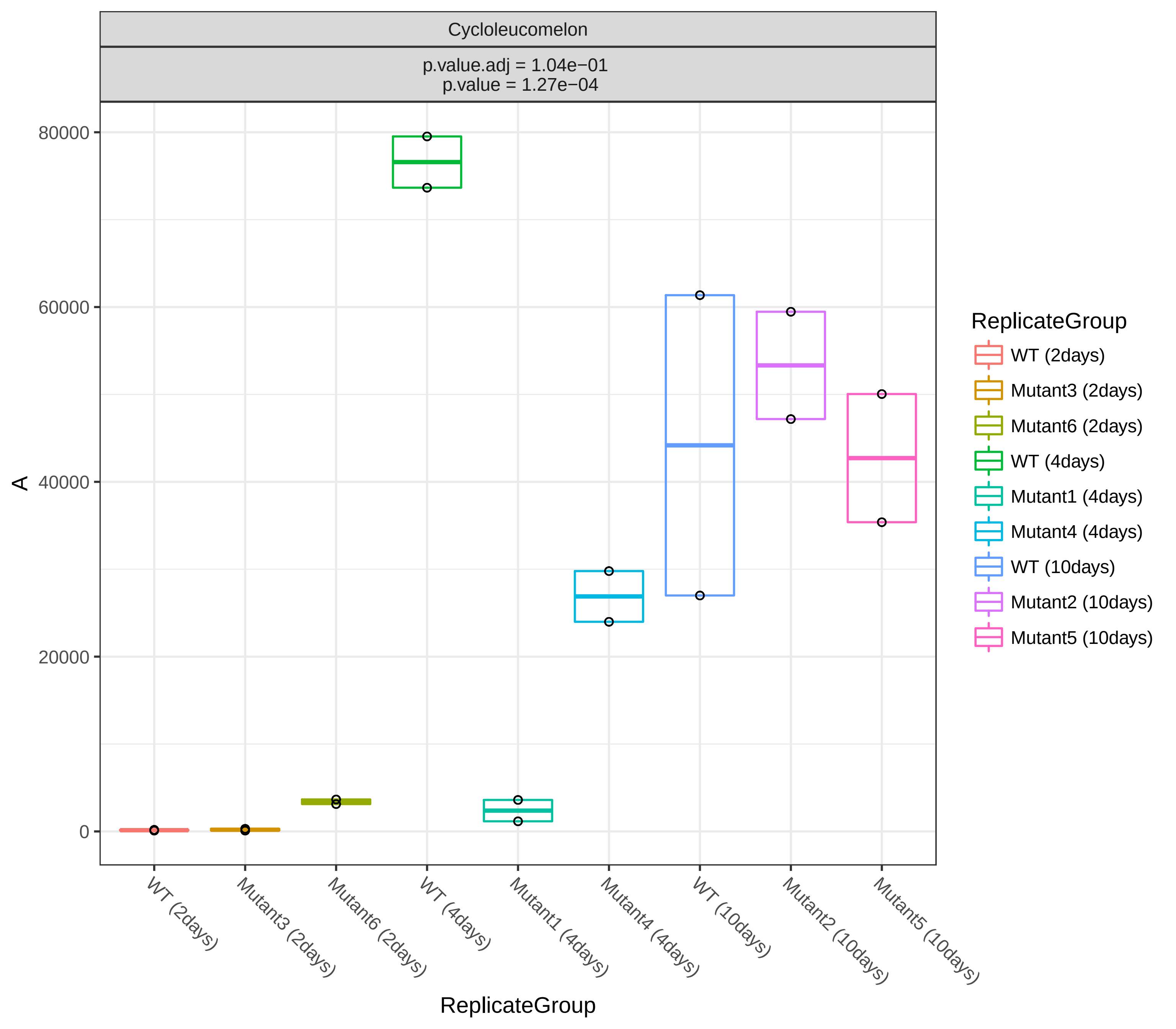

Cycloleucomelon
p.value.adj = 1.04e−01
p.value = 1.27e−04
80000
60000
40000
20000
0
A
WT (2days)
Mutant3 (2days)
Mutant6 (2days)
WT (4days)
Mutant1 (4days)
Mutant4 (4days)
WT (10days)
Mutant2 (10days)
Mutant5 (10days)
ReplicateGroup
ReplicateGroup
WT (2days)
Mutant3 (2days)
Mutant6 (2days)
WT (4days)
Mutant1 (4days)
Mutant4 (4days)
WT (10days)
Mutant2 (10days)
Mutant5 (10days)

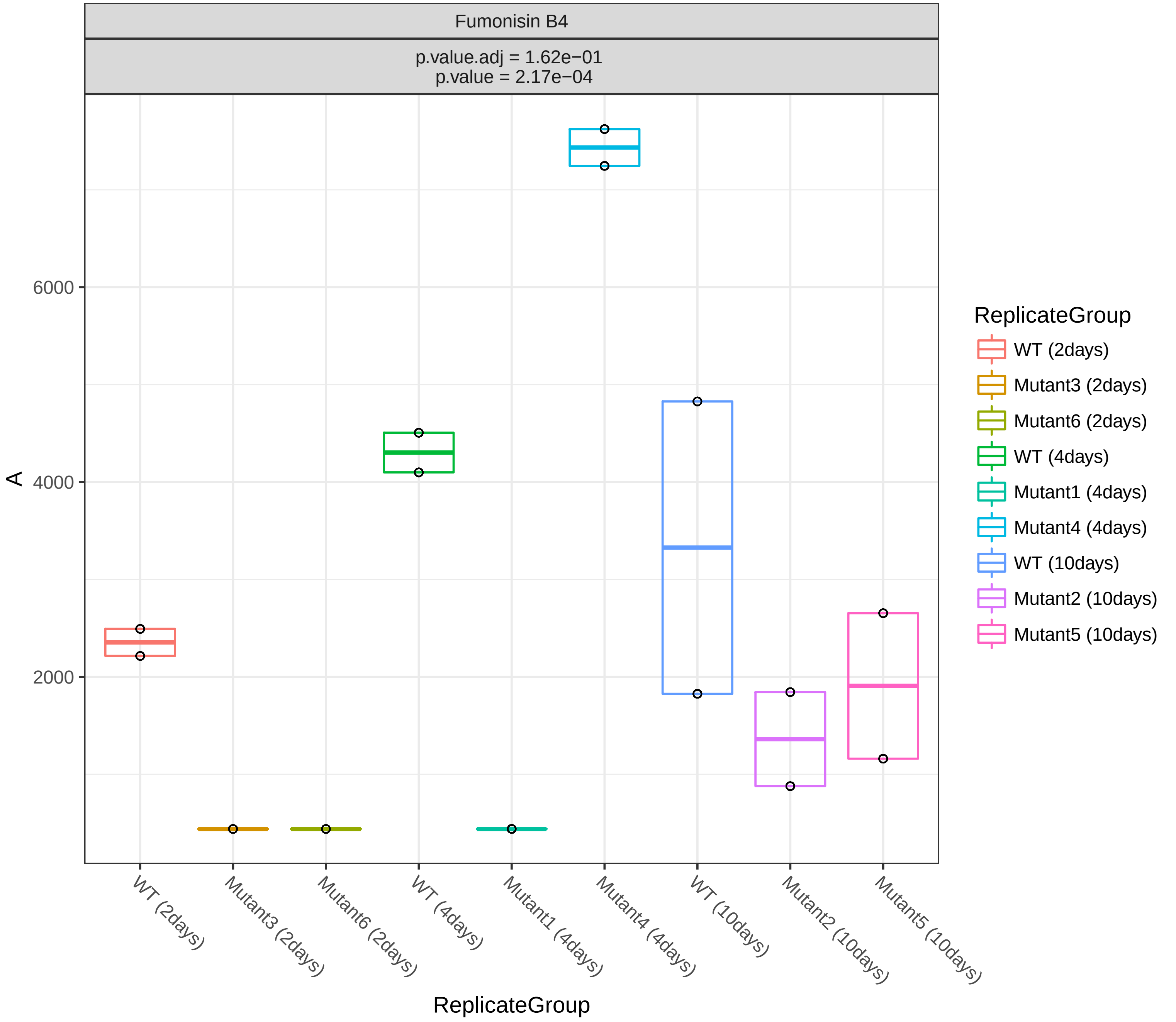
Fumonisin B4
p.value.adj = 1.62e−01
p.value = 2.17e−04
A
6000
4000
2000
ReplicateGroup
WT (2days)
Mutant3 (2days)
Mutant6 (2days)
WT (4days)
Mutant1 (4days)
Mutant4 (4days)
WT (10days)
Mutant2 (10days)
Mutant5 (10days)

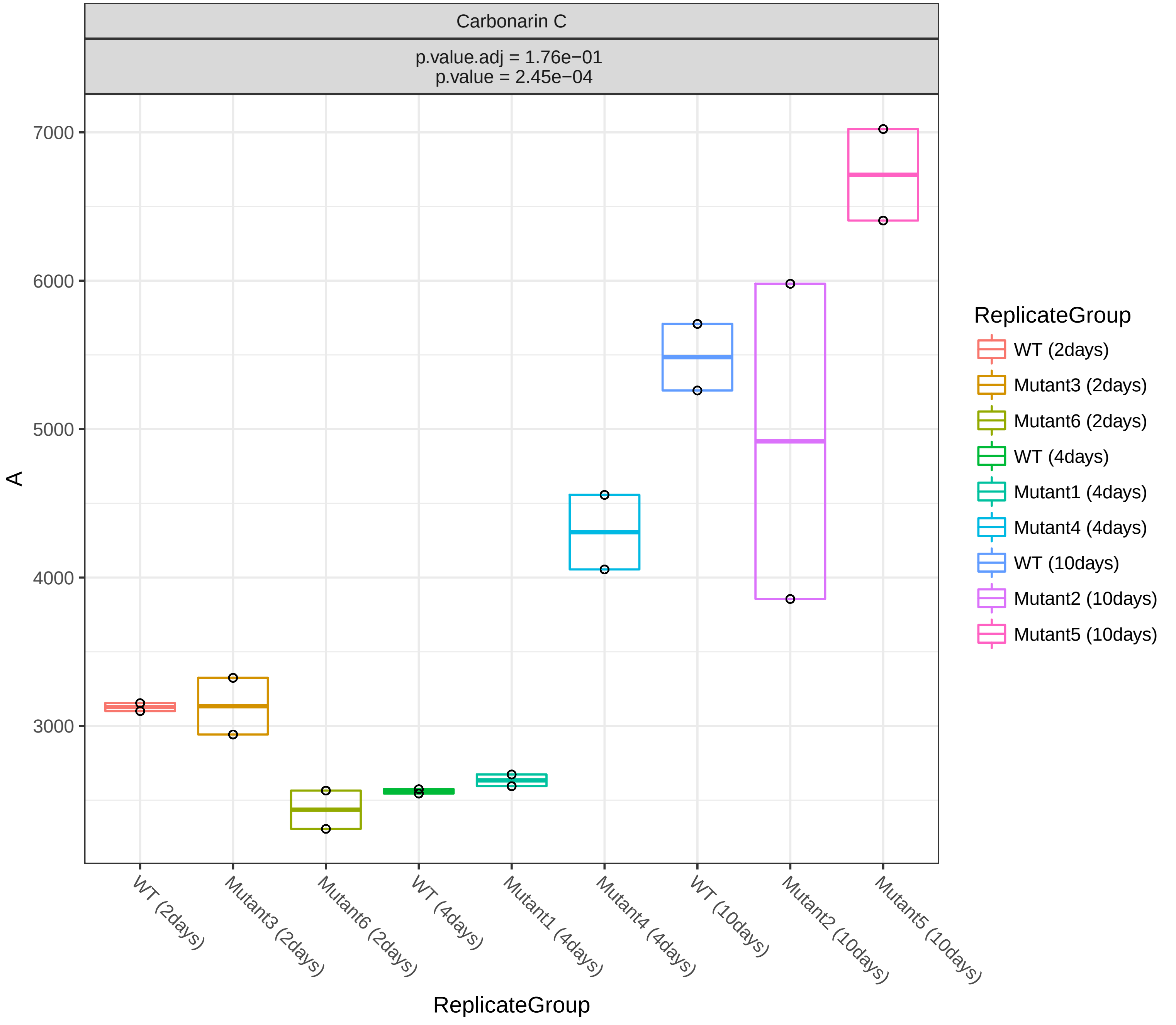
Carbonarin C
p.value.adj = 1.76e−01
p.value = 2.45e−04
7000
6000
5000
4000
3000
A
WT (2days)
Mutant3 (2days)
Mutant6 (2days)
WT (4days)
Mutant1 (4days)
Mutant4 (4days)
WT (10days)
Mutant2 (10days)
Mutant5 (10days)
ReplicateGroup
ReplicateGroup
WT (2days)
Mutant3 (2days)
Mutant6 (2days)
WT (4days)
Mutant1 (4days)
Mutant4 (4days)
WT (10days)
Mutant2 (10days)
Mutant5 (10days)

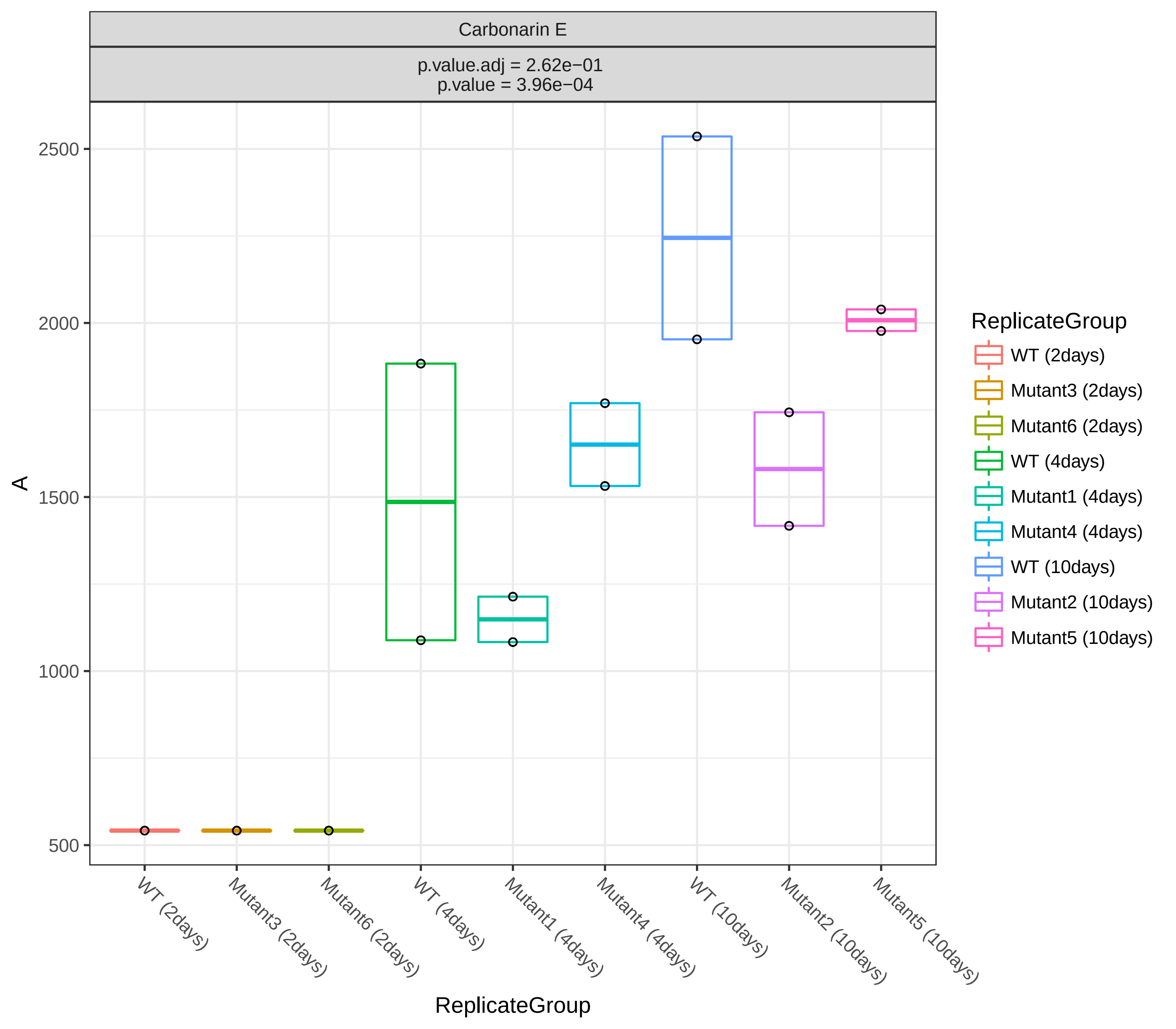

Carbonarin E
p.value.adj = 2.62e−01
p.value = 3.96e−04
A
2500
2000
1500
1000
500
ReplicateGroup
WT (2days)
Mutant3 (2days)
Mutant6 (2days)
WT (4days)
Mutant1 (4days)
Mutant4 (4days)
WT (10days)
Mutant2 (10days)
Mutant5 (10days)

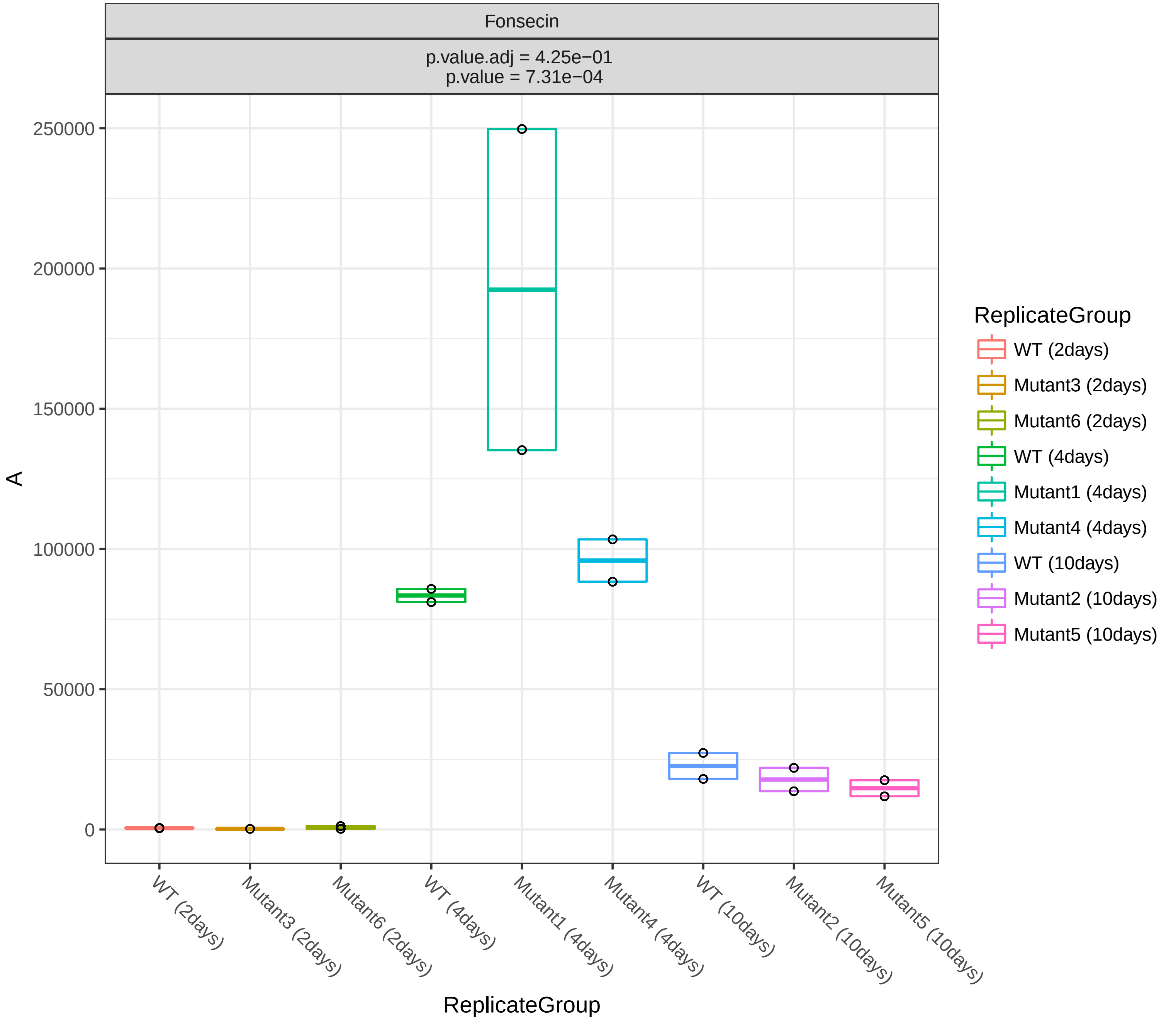

Fonsecin
p.value.adj = 4.25e−01
p.value = 7.31e−04
A
250000
200000
150000
100000
50000
0
WT (2days)
Mutant3 (2days)
Mutant6 (2days)
WT (4days)
Mutant1 (4days)
Mutant4 (4days)
WT (10days)
Mutant2 (10days)
Mutant5 (10days)
ReplicateGroup
ReplicateGroup
WT (2days)
Mutant3 (2days)
Mutant6 (2days)
WT (4days)
Mutant1 (4days)
Mutant4 (4days)
WT (10days)
Mutant2 (10days)
Mutant5 (10days)

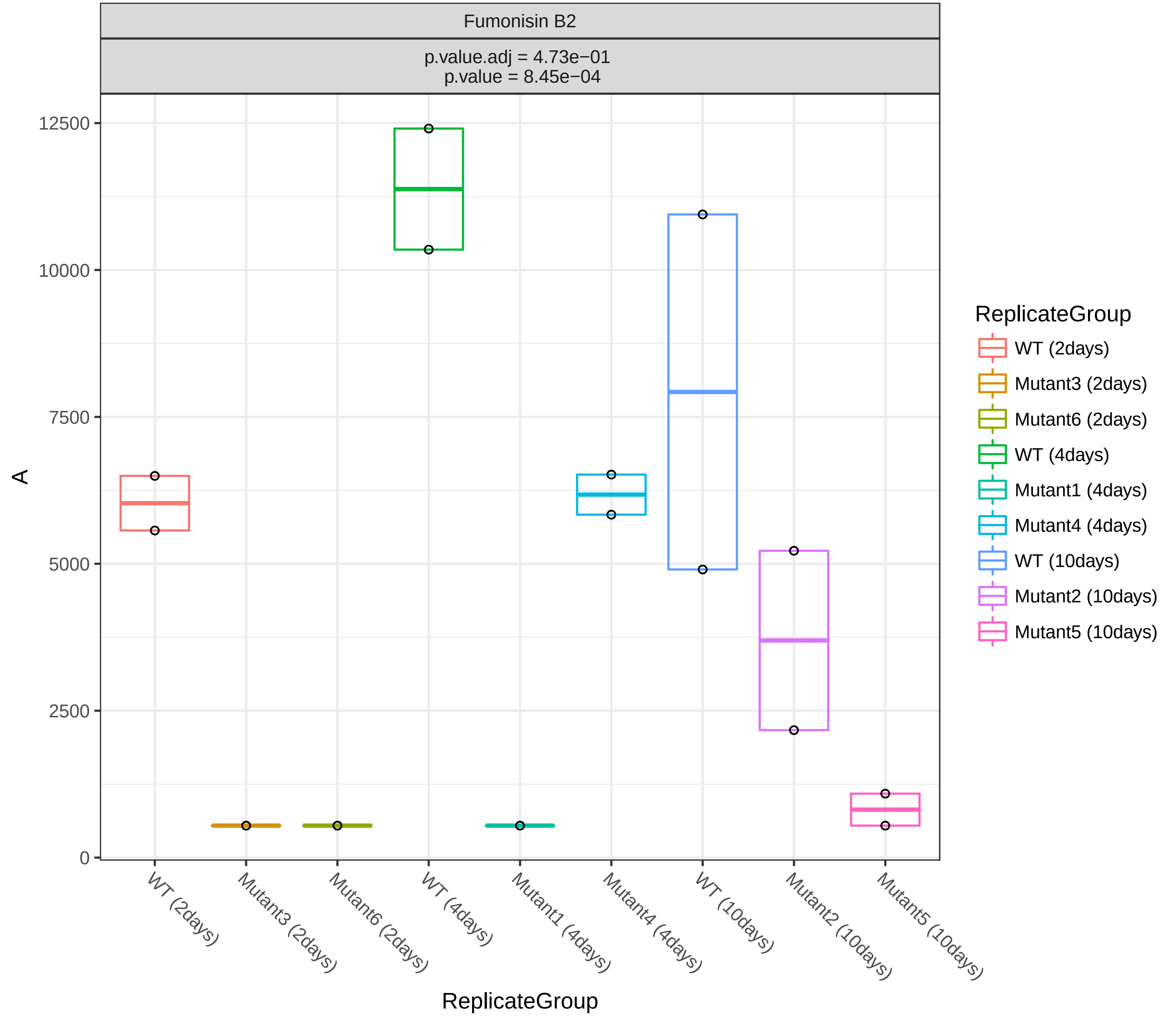

Fumonisin B2
p.value.adj = 4.73e−01
p.value = 8.45e−04
A
12500
10000
7500
5000
2500
0
WT (2days)
Mutant3 (2days)
Mutant6 (2days)
WT (4days)
Mutant1 (4days)
Mutant4 (4days)
WT (10days)
Mutant2 (10days)
Mutant5 (10days)
ReplicateGroup
ReplicateGroup
WT (2days)
Mutant3 (2days)
Mutant6 (2days)
WT (4days)
Mutant1 (4days)
Mutant4 (4days)
WT (10days)
Mutant2 (10days)
Mutant5 (10days)

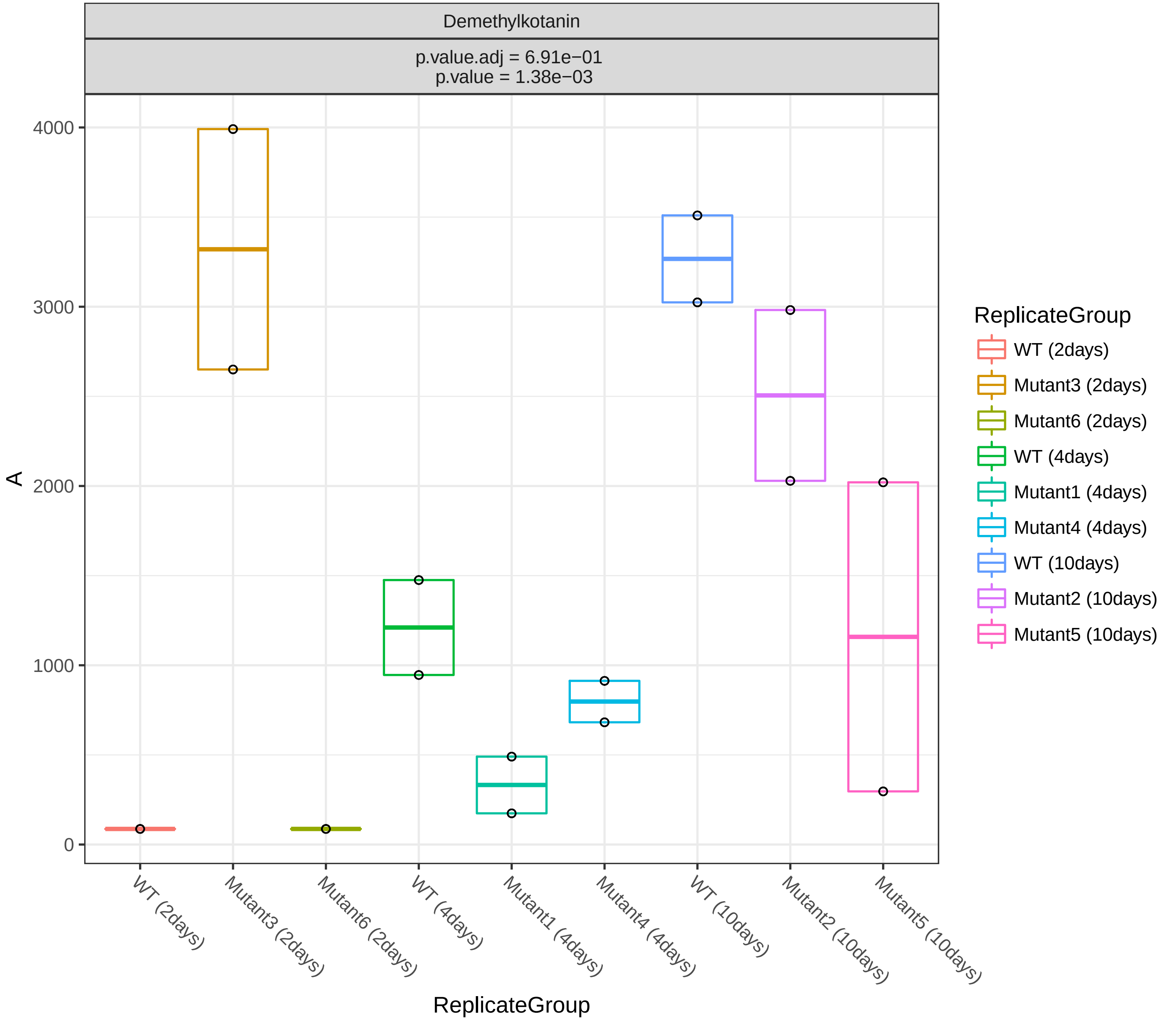
Demethylkotanin
p.value.adj = 6.91e−01
p.value = 1.38e−03
A
4000
3000
2000
1000
0
WT (2days)
Mutant3 (2days)
Mutant6 (2days)
WT (4days)
Mutant1 (4days)
Mutant4 (4days)
WT (10days)
Mutant2 (10days)
Mutant5 (10days)
ReplicateGroup
ReplicateGroup
WT (2days)
Mutant3 (2days)
Mutant6 (2days)
WT (4days)
Mutant1 (4days)
Mutant4 (4days)
WT (10days)
Mutant2 (10days)
Mutant5 (10days)

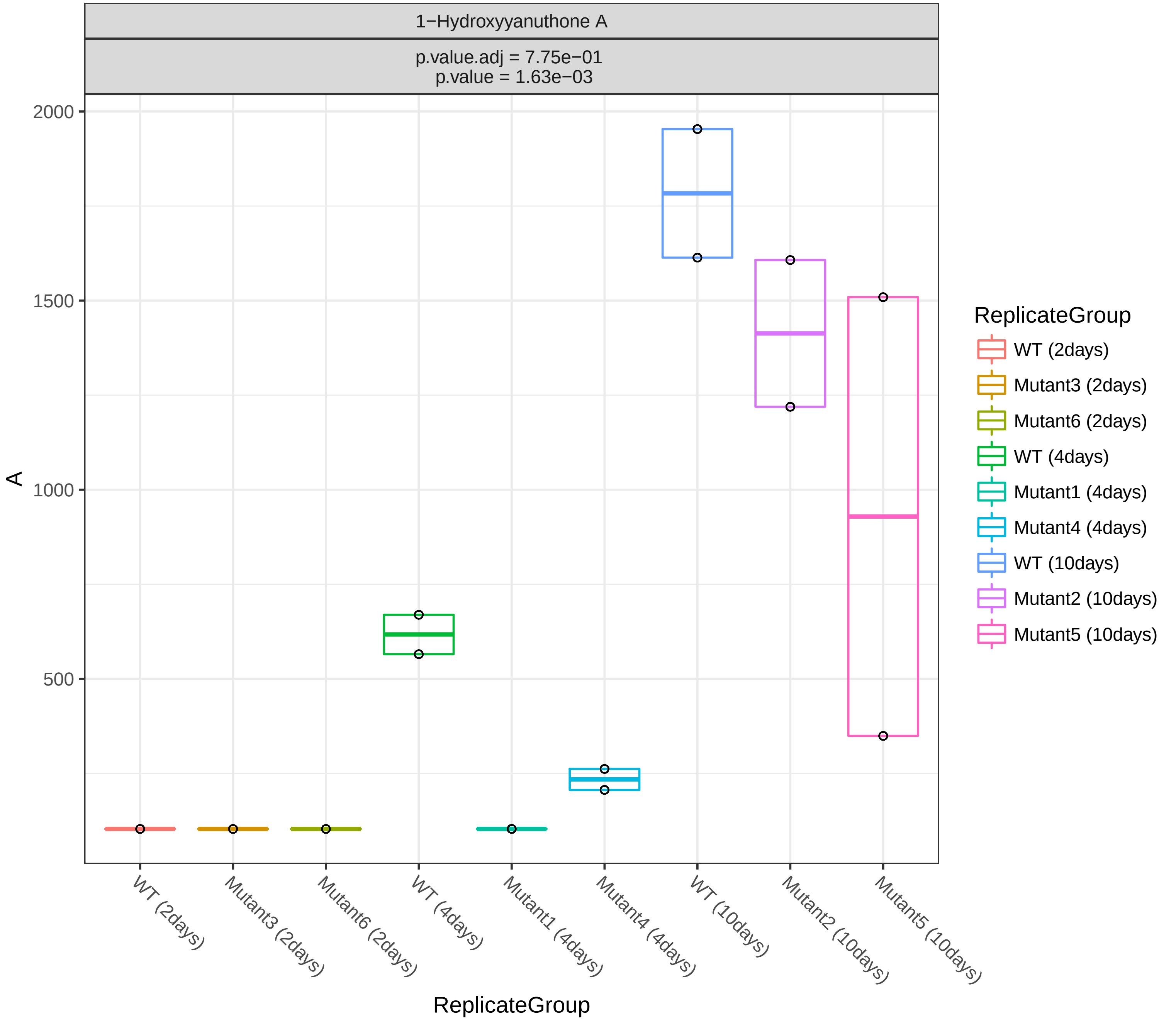

1−Hydroxyyanuthone A
p.value.adj = 7.75e−01
p.value = 1.63e−03
2000
1500
1000
500
A
WT (2days)
Mutant3 (2days)
Mutant6 (2days)
WT (4days)
Mutant1 (4days)
Mutant4 (4days)
WT (10days)
Mutant2 (10days)
Mutant5 (10days)
ReplicateGroup
ReplicateGroup
WT (2days)
Mutant3 (2days)
Mutant6 (2days)
WT (4days)
Mutant1 (4days)
Mutant4 (4days)
WT (10days)
Mutant2 (10days)
Mutant5 (10days)

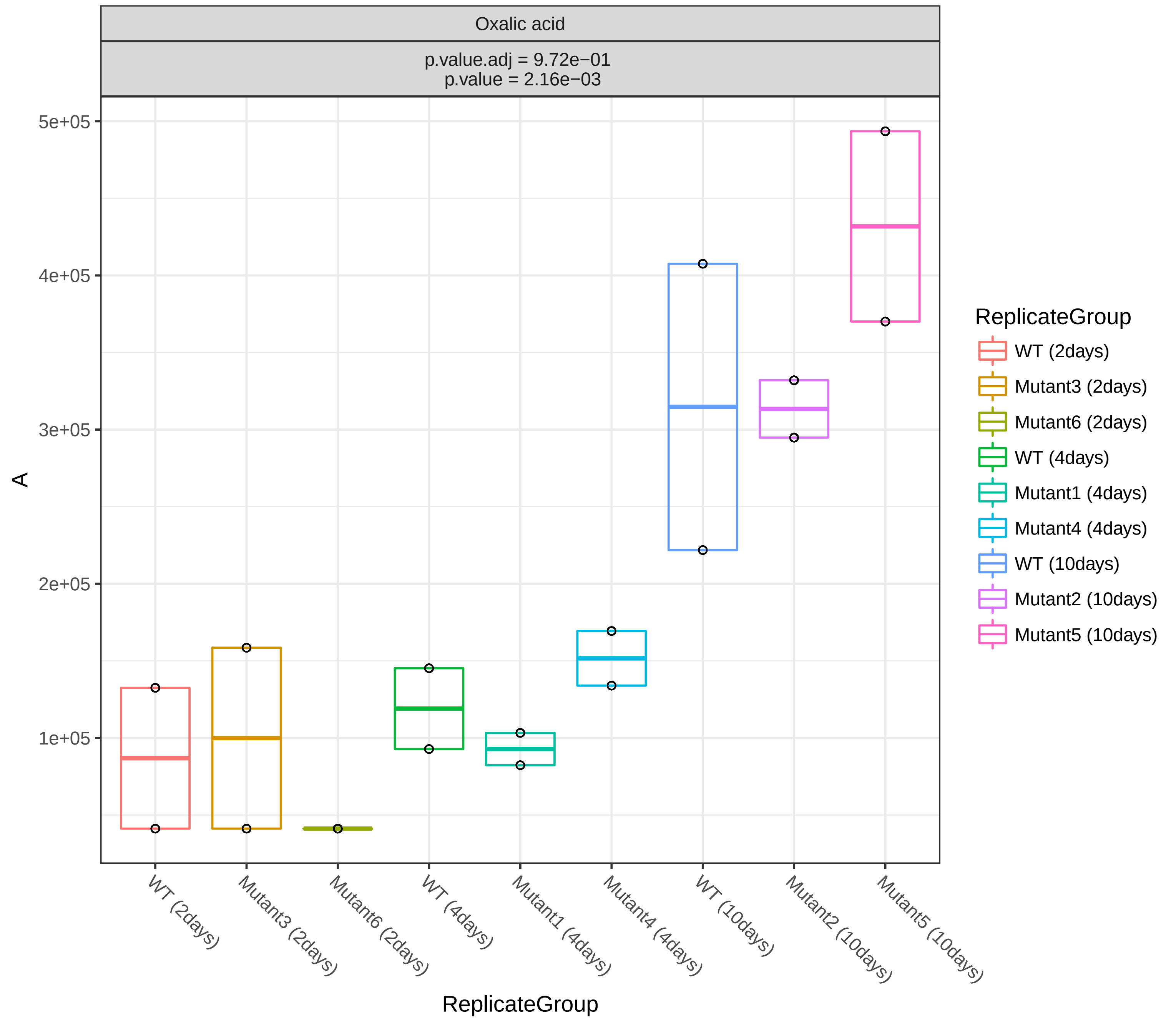

Oxalic acid
p.value.adj = 9.72e−01
p.value = 2.16e−03
5e+05
4e+05
3e+05
2e+05
1e+05
A
WT (2days)
Mutant3 (2days)
Mutant6 (2days)
WT (4days)
Mutant1 (4days)
Mutant4 (4days)
WT (10days)
Mutant2 (10days)
Mutant5 (10days)
ReplicateGroup
ReplicateGroup
WT (2days)
Mutant3 (2days)
Mutant6 (2days)
WT (4days)
Mutant1 (4days)
Mutant4 (4days)
WT (10days)
Mutant2 (10days)
Mutant5 (10days)

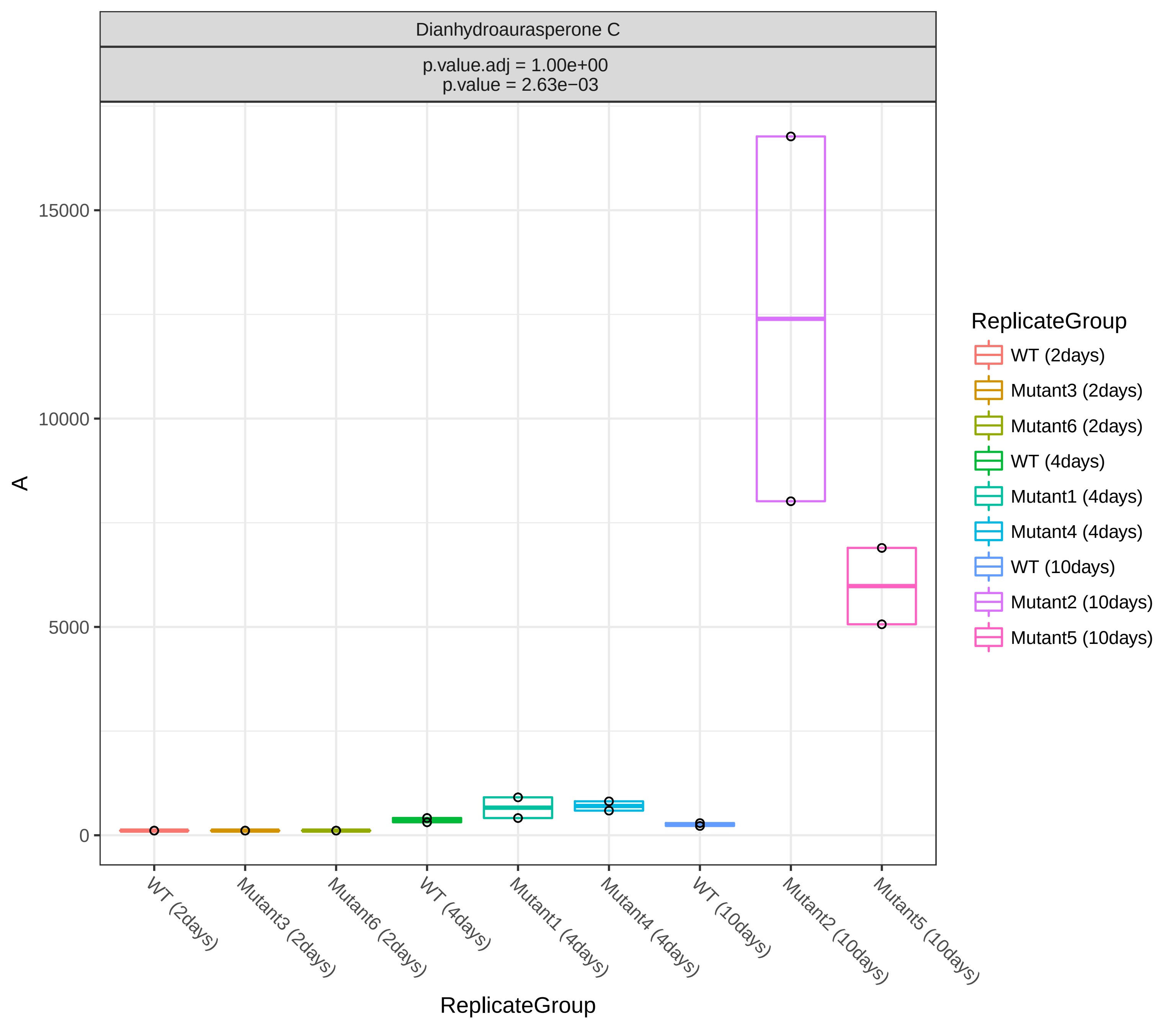
Dianhydroaurasperone C
p.value.adj = 1.00e+00
p.value = 2.63e−03
15000
10000
5000
0
A
WT (2days)
Mutant3 (2days)
Mutant6 (2days)
WT (4days)
Mutant1 (4days)
Mutant4 (4days)
WT (10days)
Mutant2 (10days)
Mutant5 (10days)
ReplicateGroup
ReplicateGroup
WT (2days)
Mutant3 (2days)
Mutant6 (2days)
WT (4days)
Mutant1 (4days)
Mutant4 (4days)
WT (10days)
Mutant2 (10days)
Mutant5 (10days)

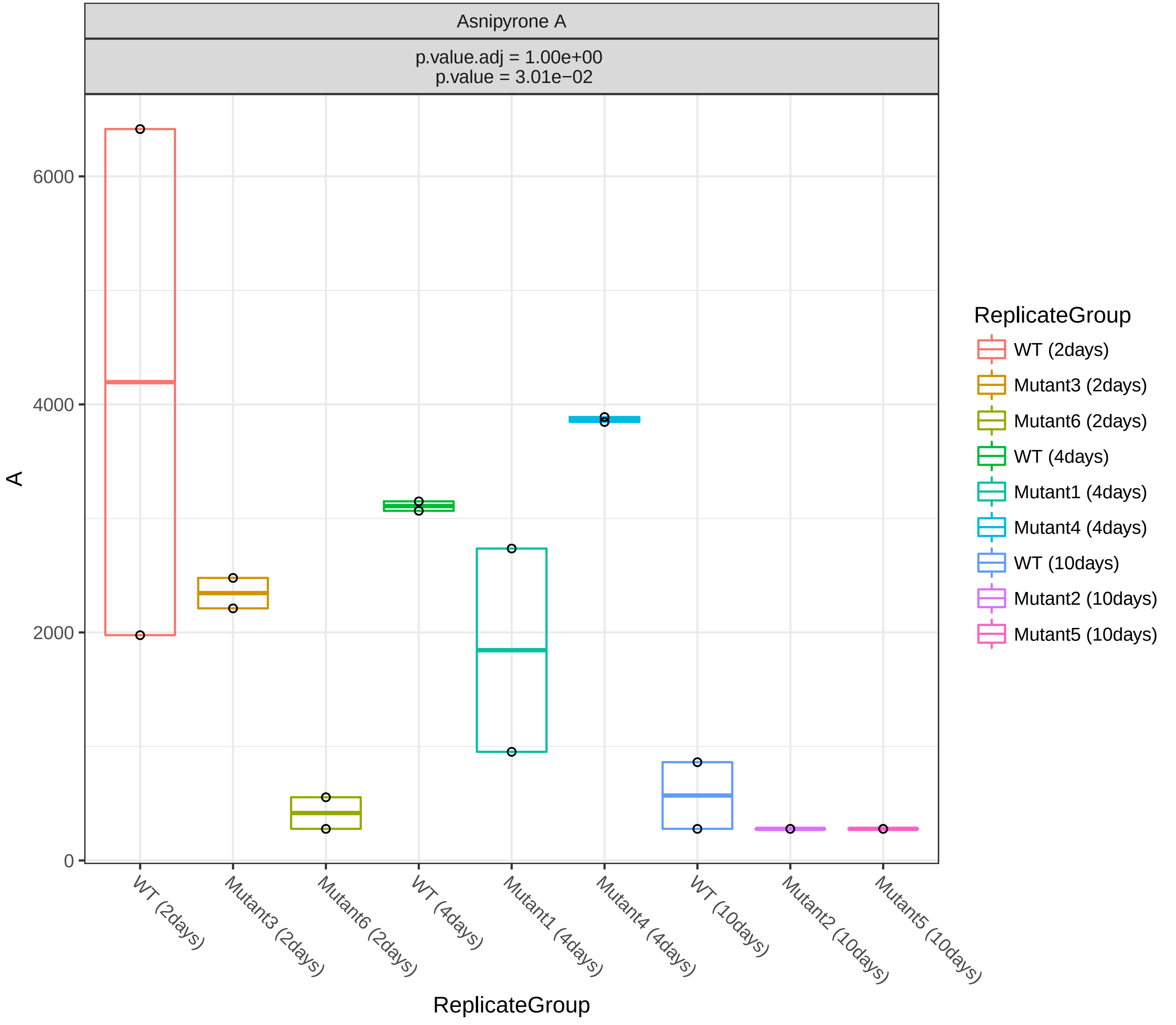
Asnipyrone A
p.value.adj = 1.00e+00
p.value = 3.01e−02
6000
4000
2000
0
A
WT (2days)
Mutant3 (2days)
Mutant6 (2days)
WT (4days)
Mutant1 (4days)
Mutant4 (4days)
WT (10days)
Mutant2 (10days)
Mutant5 (10days)
ReplicateGroup
ReplicateGroup
WT (2days)
Mutant3 (2days)
Mutant6 (2days)
WT (4days)
Mutant1 (4days)
Mutant4 (4days)
WT (10days)
Mutant2 (10days)
Mutant5 (10days)

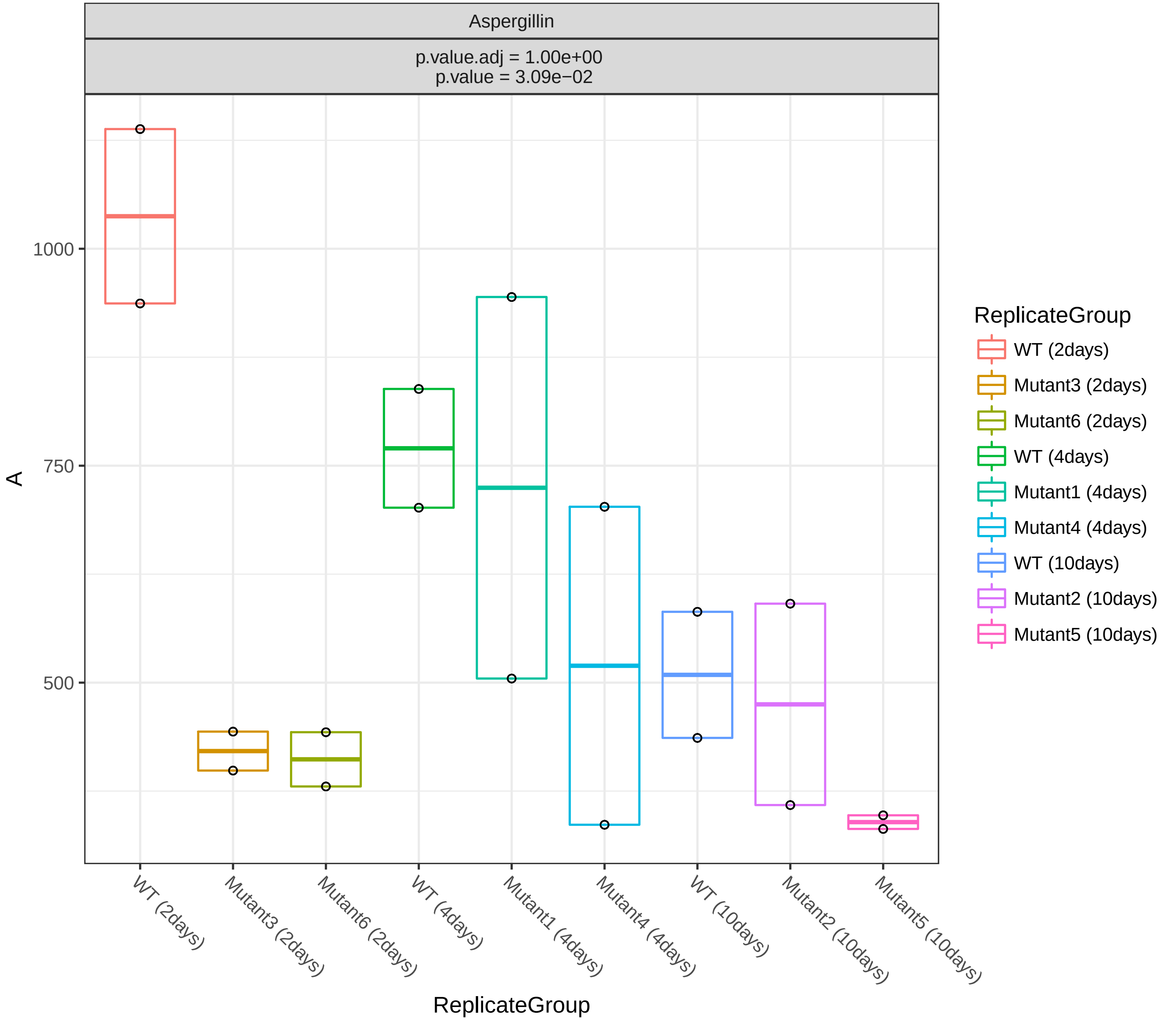
Aspergillin
p.value.adj = 1.00e+00
p.value = 3.09e−02
A
1000
750
500
ReplicateGroup
WT (2days)
Mutant3 (2days)
Mutant6 (2days)
WT (4days)
Mutant1 (4days)
Mutant4 (4days)
WT (10days)
Mutant2 (10days)
Mutant5 (10days)
ReplicateGroup
WT (2days)
Mutant3 (2days)
Mutant6 (2days)
WT (4days)
Mutant1 (4days)
Mutant4 (4days)
WT (10days)
Mutant2 (10days)
Mutant5 (10days)

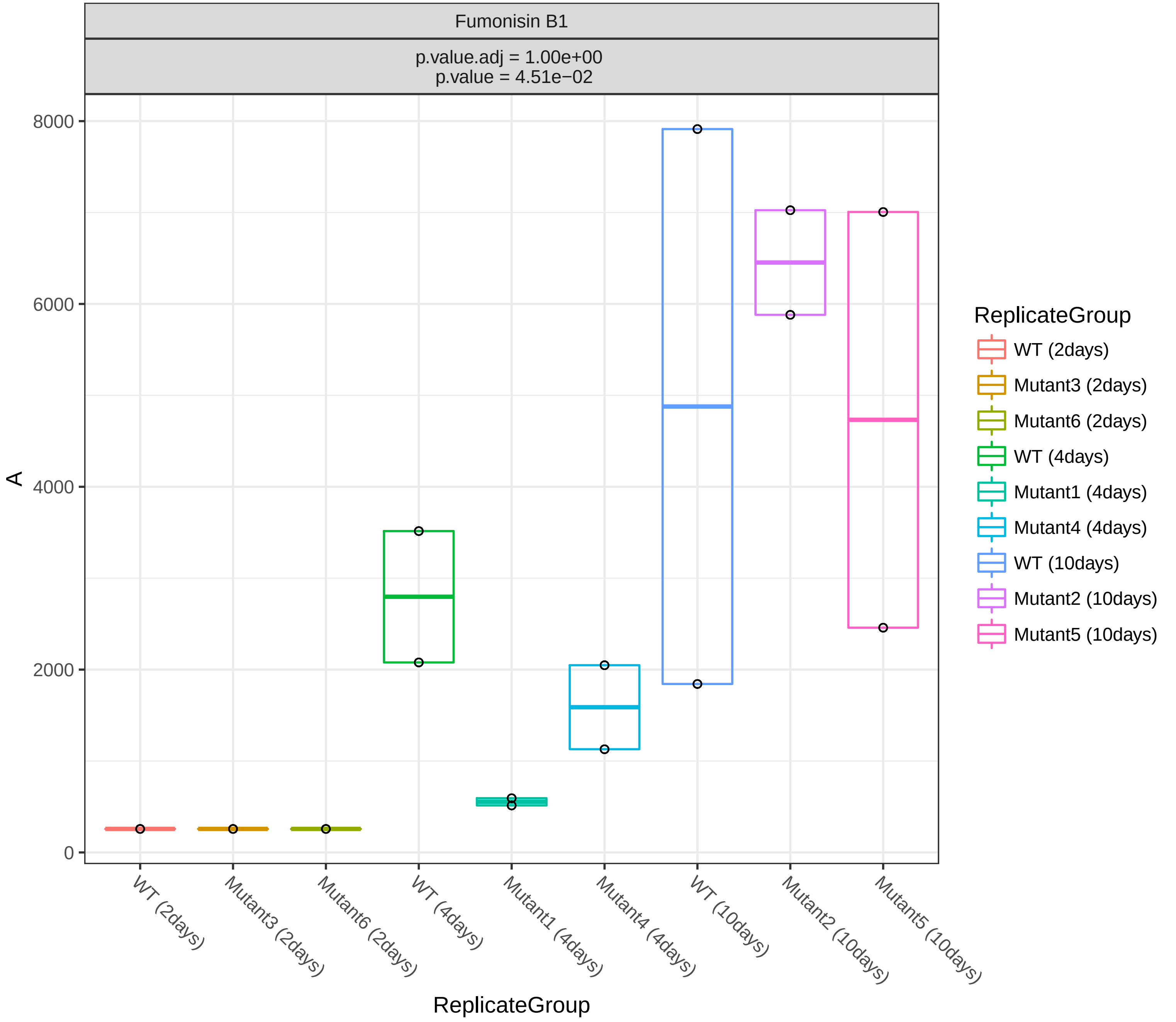
Fumonisin B1
p.value.adj = 1.00e+00
p.value = 4.51e−02
A
0
2000
4000
6000
8000
WT (2days)
Mutant3 (2days)
Mutant6 (2days)
WT (4days)
Mutant1 (4days)
Mutant4 (4days)
WT (10days)
Mutant2 (10days)
Mutant5 (10days)
ReplicateGroup
ReplicateGroup
WT (2days)
Mutant3 (2days)
Mutant6 (2days)
WT (4days)
Mutant1 (4days)
Mutant4 (4days)
WT (10days)
Mutant2 (10days)
Mutant5 (10days)

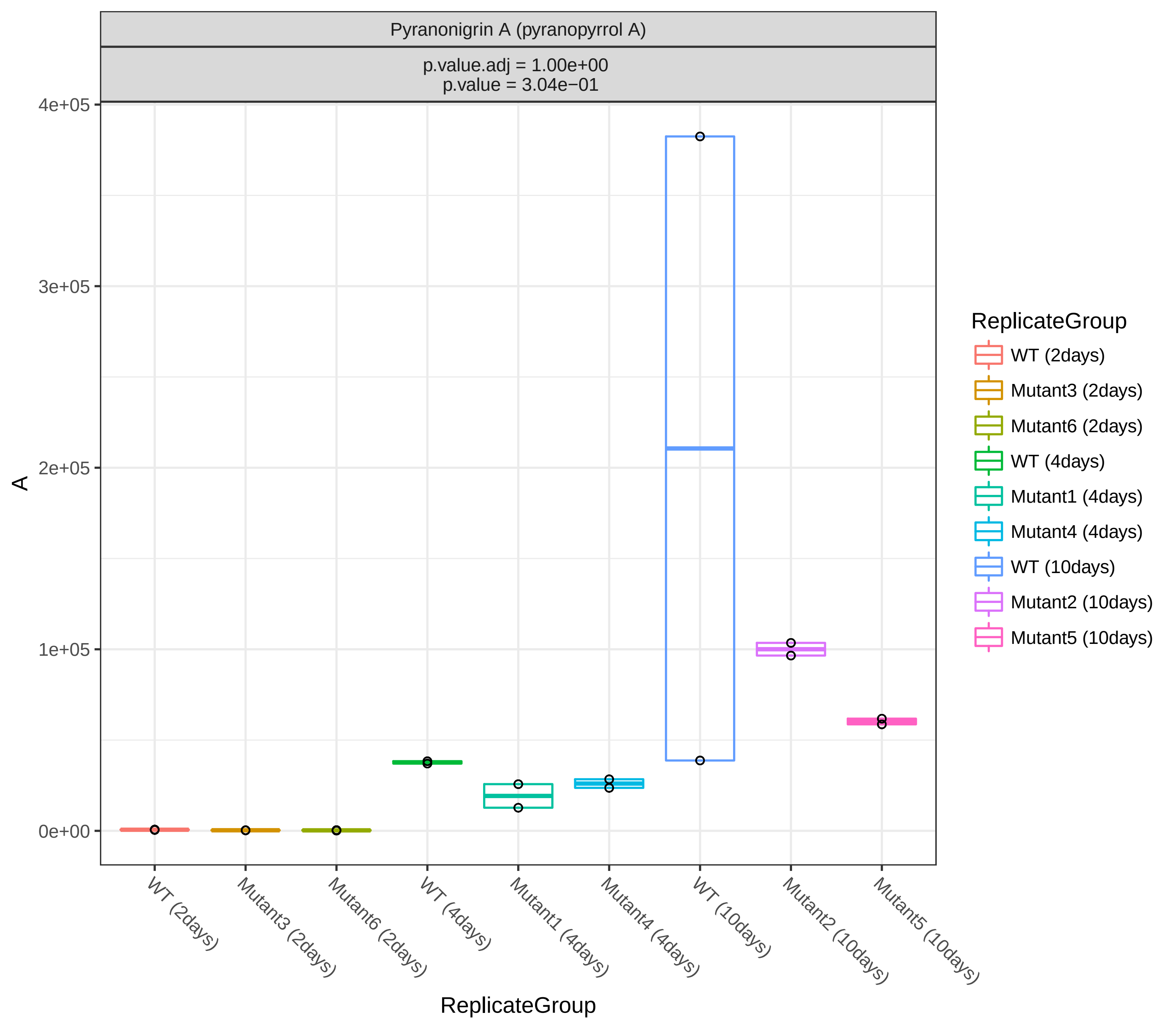

Pyranonigrin A (pyranopyrrol A)
p.value.adj = 1.00e+00
p.value = 3.04e−01
4e+05
3e+05
2e+05
1e+05
0e+00
A
WT (2days)
Mutant3 (2days)
Mutant6 (2days)
WT (4days)
Mutant1 (4days)
Mutant4 (4days)
WT (10days)
Mutant2 (10days)
Mutant5 (10days)
ReplicateGroup
ReplicateGroup
WT (2days)
Mutant3 (2days)
Mutant6 (2days)
WT (4days)
Mutant1 (4days)
Mutant4 (4days)
WT (10days)
Mutant2 (10days)
Mutant5 (10days)

Bibliography for Supplementary Materials

Bos, C. J., Debets, A. J., Swart, K., Huybers, A., Kobus, G., & Slakhorst, S. M. (1988). Genetic analysis and the construction of master strains for assignment of genes to six linkage groups in *Aspergillus niger*. *Current Genetics*, *14*(5), 437–443.

Carvalho, N. D. S. P., Arentshorst, M., Jin Kwon, M., Meyer, V., & Ram, A. F. J. (2010). Expanding the *ku70* toolbox for filamentous fungi: establishment of complementation vectors and recipient strains for advanced gene analyses. *Applied Microbiology and Biotechnology*. https://doi.org/10.1007/s00253-010-2588-1

Fiedler, M. R. M., Gensheimer, T., Kubisch, C., & Meyer, V. (2017). HisB as novel selection marker for gene targeting approaches in *Aspergillus niger*. *BMC Microbiology*, *17*(1), 57. https://doi.org/10.1186/s12866-017-0960-3

Schäpe, P., Kwon, M. J., Baumann, B., Gutschmann, B., Jung, S., Lenz, S., … Meyer, V. (2019). Updating genome annotation for the microbial cell factory *Aspergillus niger* using gene co-expression networks. *Nucleic Acids Research*. https://doi.org/10.1093/nar/gky1183

van Hartingsveldt, W., Mattern, I. E., van Zeijl, C. M. J., Pouwels, P. H., & van den Hondel, C. A. M. J. J. (1987). Development of a homologous transformation system for *Aspergillus niger* based on the *pyrG* gene. *MGG Molecular & General Genetics*, *206*(1), 71–75. https://doi.org/10.1007/BF00326538
